# Wendan decoction modulates *Parasutterella* to influence fatty acid metabolism in MAFLD via the FXR/PPARα/CYP4A12A axis

**DOI:** 10.1186/s13020-026-01474-1

**Published:** 2026-07-20

**Authors:** Yiheng Zhang, Jiahao Wang, Weihao Zhu, Yi Zhang, Tianle Ma, Huihua Fang, Jicong Du, Zhipeng Chen, Weidong Li, Xiao Zhang, Li Wu, Xin Chen, Haibing Hua

**Affiliations:** 1 Jiangyin Hospital of Chinese Medicine Affiliated to Nanjing University of Chinese Medicine, Nanjing University of Chinese Medicine, Jiangyin, 214400 China; 2 Jiangsu Key Laboratory for Pharmacology and Safety Research of Chinese Materia Medica, School of Pharmacy, Nanjing University of Chinese Medicine, Nanjing, 210023 China; 3 School of Pharmacy, Nanjing University of Chinese Medicine, Nanjing, 210023 China; 4 Institute of Literature in Nanjing University of Chinese Medicine, Nanjing, 210023 China; 5 College of Pharmaceutical Sciences, Soochow University, Suzhou, 215123 Jiangsu China; 6 Jiangsu Province Hospital of Chinese Medicine, Affiliated Hospital of Nanjing University of Chinese Medicine, Nanjing, 210004 China; 7 Wuxi Xishan District Hospital of Traditional Chinese Medicine, Wuxi, 214194 China

**Keywords:** Wendan decoction, MAFLD, *Parasutterella*, 7α-OH-T, FXR/PPARα/CYP4A12A axis

## Abstract

**Background:**

The host microbiota and hepatic drug-metabolizing enzymes are important mediators of the metabolism and biological effects of herbal components. Through bidirectional interactions, herbal medicines can also reshape the host microbial community. The clinical efficacy of Wendan Decoction (WDD) in treating metabolic dysfunction-associated fatty liver disease (MAFLD) has been well established. However, its interactions with the host microbiota through the gut-liver axis remain unclear.

**Purpose:**

This study aimed to investigate the mechanism by which WDD modulates host microbial activity through the gut-liver axis to ameliorate MAFLD.

**Methods:**

MAFLD models were established by high-fat diet (HFD) feeding and subsequently treated with WDD, *Parasutterella excrementihominis* (*P. excrementihominis*), or 7α-OH-T. The ABX group underwent antibiotic-mediated microbiota depletion before treatment. Multi-omics analyses were used to characterize the dynamic trajectories of microbiota-derived metabolites. These analyses included targeted bile acid (BA) profiling of serum, 16S rRNA gene sequencing and untargeted metabolomics of cecal contents, and proteomics and untargeted metabolomics of liver tissue. Hematoxylin and eosin, Oil Red O, and Alcian blue-periodic acid-Schiff staining were used to assess pathological changes in the liver and intestinal tissues during MAFLD. ELISA, Western blotting, and other assays were performed to quantify markers of inflammation and lipid metabolism. Following UPLC/UV detection of 7α-OH-T in portal vein serum, molecular docking and molecular dynamics simulations, together with cellular thermal shift assays (CETSA) and microscale thermophoresis (MST), were used to validate FXR as a target of 7α-OH-T.

**Results:**

WDD alleviated hepatic steatosis, intestinal inflammation, and barrier dysfunction in MAFLD, but these effects depended on the integrity of the host microbiota. 16S rRNA gene sequencing showed that WDD promoted the growth of beneficial bacteria, including *Bacteroides* and *Parasutterella*. Combined analysis of targeted serum BA metabolomics and untargeted metabolomics of cecal contents indicated that WDD-mediated modulation of the host microbiota reduced the total serum BA load, increased alternative-pathway metabolites, including CDCA and TCDCA, in the liver and intestine, and decreased toxic secondary BAs, including DCA and LCA. Steroid and fatty acid metabolites, such as 7α-OH-T, were also increased. *Pearson* correlation analysis and *P. excrementihominis* transplantation experiments suggested that the increase in 7α-OH-T was closely associated with *P. excrementihominis*. Untargeted liver metabolomics and serological analyses confirmed that gut-derived 7α-OH-T entered the liver through the portal vein and acted on hepatic targets via the gut-liver axis. In animal experiments involving exogenous 7α-OH-T supplementation and in MAFLD THLE-2 cell models treated with 7α-OH-T, 7α-OH-T ameliorated hepatic lipid accumulation and promoted lipid utilization in THLE-2 cells. A series of interaction assays, including CETSA and MST, identified FXR as a target of 7α-OH-T. Furthermore, 7α-OH-T markedly activated the FXR/PPARα/CYP4A12A axis and served as a key messenger through which WDD-mediated regulation of *Parasutterella* alleviated MAFLD via the gut-liver axis.

**Conclusions:**

WDD increased the abundance of *P. excrementihominis* and the level of the potentially associated metabolite 7α-OH-T. Through the portal circulation, 7α-OH-T promoted gut-liver crosstalk and targeted the FXR/PPARα/CYP4A12A axis, thereby ameliorating MAFLD.

**Graphical Abstract:**

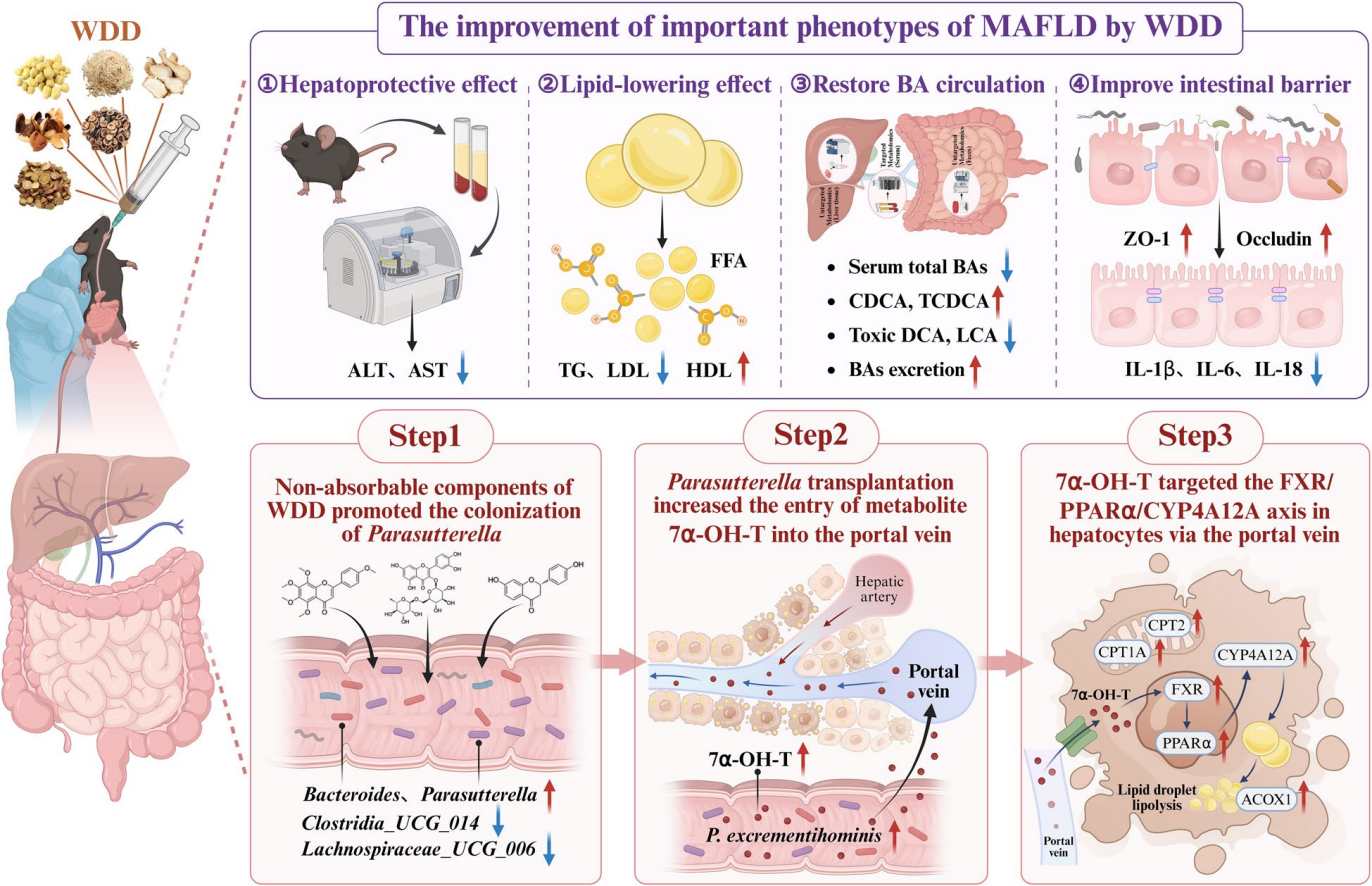

**Supplementary Information:**

The online version contains supplementary material available at 10.1186/s13020-026-01474-1.

## Highlights

- WDD improved lipid deposition in MAFLD mice partially dependent on host microbial integrity.
- WDD reshaped BA circulation and fatty acid metabolism in MAFLD mice.
- *P. excrementihominis* (the representative of *Parasutterella*), regulated by WDD, colonized the intestine and had the potential to modulate BA and fatty acid metabolism.
- Elevated 7α-OH-T after *P. excrementihominis* transplantation alleviated MAFLD by targeting the FXR/PPARα/CYP4A12A axis.

**Supplementary Information:**

The online version contains supplementary material available at 10.1186/s13020-026-01474-1.

## Introduction

Metabolic dysfunction-associated fatty liver disease (MAFLD) is a major form of chronic liver disease [1]. Recent epidemiological evidence indicates that the global prevalence of MAFLD among adults has reached 33.6% [2] and continues to rise. China has the largest population of patients with MAFLD worldwide, and this number is projected to exceed 310 million by 2030 [3]. The disease spectrum of MAFLD ranges from simple hepatic steatosis to metabolic dysfunction-associated steatohepatitis, liver fibrosis, and ultimately hepatocellular carcinoma (HCC). MAFLD has gradually surpassed viral hepatitis as the most prevalent chronic liver disease in China. Given this growing disease burden, elucidating the pathogenesis of MAFLD and developing multi-target, low-toxicity therapeutic strategies have become major priorities in hepatology.

Common predisposing factors for MAFLD include a high-calorie diet, genetic susceptibility, and insulin resistance [4, 5]. Its pathogenesis involves multiple processes, including impaired fatty acid oxidation (FAO), dysregulated bile acid (BA) metabolism, and inflammatory responses. Recently, the gut microbiota has attracted considerable attention as a key regulator of host metabolism and MAFLD progression. Patients with MAFLD commonly exhibit gut dysbiosis, characterized by reduced microbial diversity, depletion of beneficial bacteria, and enrichment of harmful bacteria. The severity of this imbalance is positively correlated with the degree of hepatic steatosis [6]. Notably, gut dysbiosis is closely associated with disrupted enterohepatic circulation and abnormal BA metabolism. Specific gut microorganisms convert primary BAs into highly toxic secondary metabolites, which enter the liver through the portal vein and further exacerbate hepatic steatosis and inflammation [7]. Therefore, therapeutic strategies for MAFLD should focus not only on hepatic metabolism but also on modulating the gut microbiota and reshaping the microbiota-BA-FAO metabolic axis. In this context, traditional Chinese medicine (TCM), with its multicomponent, multitarget, and holistic regulatory properties, may offer new therapeutic approaches for MAFLD.

According to TCM theory, MAFLD is closely associated with “phlegm-dampness,” a pathological product of spleen-stomach disharmony and disrupted qi dynamics. Wendan Decoction (WDD), a classical formula for treating phlegm-related disorders, was first documented in Ji Yan Fang during the Southern and Northern Dynasties. It contains Pinelliae Rhizoma Praeparatum as the sovereign herb to resolve phlegm, dry dampness, and regulate gastrointestinal function; Bambusae Caulis in Taenias as the minister herb to clear heat and relieve nausea; Aurantii Fructus Immaturus as an assistant herb to promote qi movement and resolve food stagnation; Citri Reticulatae Pericarpium to regulate qi and strengthen the spleen; Glycyrrhizae Radix et Rhizoma as the envoy herb to harmonize the formula; and Zingiberis Rhizoma Recens to resolve phlegm and reduce the toxicity of Pinelliae Rhizoma Praeparatum. Previous studies have validated the efficacy of WDD in MAFLD and other metabolic diseases and have supported the standardization of its constituent components [8, 9].

An increasing number of studies have shown that TCM components can interact with the gut microbiota in addition to being absorbed into the bloodstream. The resulting metabolites may regulate hepatic function through the gut-liver axis. We therefore hypothesized that WDD alleviates MAFLD by remodeling the host microbiota, restoring BA and fatty acid metabolic homeostasis, and improving gut-liver function. To test this hypothesis, we integrated untargeted metabolomics across multiple sample types with 16S rRNA gene sequencing and proteomic analysis. Single-bacterium transplantation and metabolite supplementation experiments were further conducted to elucidate the molecular mechanisms underlying the therapeutic effects of WDD on MAFLD at the microbial and metabolic levels.

## Materials and methods

### Reagents

The chemicals were listed as follows: WDD (Formula was listed in Table. S1) was provided by Jiangyin Tianjiang Pharmaceutical Co., Ltd. (No. ZYPFKL202401); Rosiglitazone Hydrochloride Capsules (Orohua; Lot: 24120182); Choline and L-amino acid-sufficient purified diet (XTMRCD10-C) and Choline-deficient, L-amino acid-defined high-fat diet (XTMRCD60) were purchased from Jiangsu Xietong Pharmaceutical Bio-Engineering Co., Ltd.; Vancomycin hydrochloride (HY-17362R), Metronidazole (HY-B0318R), Ampicillin sodium (HY-B0522A) and Neomycin sulfate (HY-B0470R) were purchased from MedChemExpress; Glycyrrhetic acid (RS00711120), Luteolin-7-O-*β*-D-glucoside (RS00331120), Naringin (ST00210120), Hesperidin (RS01301120), 6-Gingerol (RS05741100) and Glycyrrhizic acid (RS00661020) were purchased from Shanghai Standard Technology Co., Ltd; Columbia blood agar base (HZB875031), Defibrinated sheep blood (HZB875645), Chopped meat carbohydrate broth (DSMZ Medium 110) (HZB875586), Chopped (Beef) meat particles (HZB875648), Ferriheme chloride (HZB875647), Vitamin K1 (HZB875646) and L-cysteine hydrochloride solution (HZB875650) were purchased from HZBio, Microbial Conservation; One Step Mouse Genotyping Kit V2 (PD111-01) was purchased from Vazyme BioTECH; IL-6 ELISA kit (JX-2899A1), IL-1β ELISA kit (JX-2776A1) and IL-18 ELISA kit (JX-2905A1) were purchased from Yancheng Junxing Biotechnology Co., Ltd; Cell Counting Kit-8 (ZYCD002-0500) was purchased from ZUNYAN; 7α-OH-T (RM550027T) was purchased from Hubei Yangxin Pharmaceutical Technology Co., Ltd; Simulated gastric fluid (pH = 3.0) (R28478-500 ml) and Simulated intestinal fluid (pH = 6.8) (R22156-100 ml) were purchased from Shanghai yuanye Bio-Technology Co., Ltd; Enhanced WB antibody diluent (KGC4309-500) was purchased from KeyGEN BioTECH; GoldBand 3-color High Range Protein Marker (20352ES76) was purchased from Yeasen Biotechnology (Shanghai) Co., Ltd; FXR recombinant protein was synthesized by Novoprotein Scientific Inc (Shanghai, China).

The antibodies were listed as follows: ActivAbTMAnti-TJP1 Polyclonal Antibody (K008065P; Solarbio^®^, Beijing, China); Caspase-1 Polyclonal Antibody (BS65859; Bioworld, Nanjing, China); Rabbit Monoclonal Antibody to Cleaved N-terminal GSDMD (50891; Promab); Rabbit Monoclonal Antibody to GSDMD (P23652; Promab); ZO1 rabbit pAb (A28491; ABclonal Technology, Wuhan, China); Anti-Occludin Antibody (A01246-4; Bosterbio); CYP7B1 Rabbit pAb (A17872; ABclonal Technology, Wuhan, China); CYP8B1 Rabbit Polyclonal Antibody (BD-PT1242; Biodragon); BSEP Monoclonal antibody (67512-1-Ig; proteintech^®^); PPAR alpha Rabbit mAb (db16114; Diagbio); FXR/NR1H4 Rabbit mAb (A24015; ABclonal Technology, Wuhan, China); Cyp4a12a Polyclonal antibody (51157-1-AP; proteintech^®^); Anti-CPT1A antibody (ab128568; Abcam, UK); Anti-ACOX1 antibody (ab184032; Abcam, UK); CPT2 Recombinant Rabbit mAb (KU) (BS48303; Bioworld, Nanjing, China); CYP7A1 Polyclonal antibody (18054-1-AP; proteintech^®^).

### Preparation of WDD

WDD formula granules were prepared as follows. The medicinal slices were decocted twice with 15- and 12-fold volumes of purified water, respectively. The combined filtrates were concentrated under reduced pressure to a specific gravity of 1.08–1.10. The concentrate was then spray-dried, passed through an 80-mesh sieve, and subjected to dry granulation.

According to the requirements of the 2025 edition of the Chinese Pharmacopoeia, six representative components of WDD were analyzed, with one component selected from each medicinal herb. Separation was performed on a C_18_ column (2 cm × 75 μm, 3 μm) at a flow rate of 0.4 mL/min. The detection conditions for glycyrrhetic acid, luteolin-7-O-*β*-D-glucoside, naringin, hesperidin, 6-gingerol, and glycyrrhizic acid were as follows: mobile phase A consisted of 0.1% formic acid in water, and mobile phase B consisted of acetonitrile. The gradient was 5% B at 0.0–10.0 min, 35–65% B at 10.0–15.0 min, 65–100% B at 15.0–19.0 min, and 100–5% B at 19.0–21.0 min. All six representative components were successfully detected (Fig. S1). The non-absorbable components of WDD are listed in Table S2.

The clinical dose of WDD formula granules in humans was 6.4 g/day, equivalent to 0.1 g/kg. According to the standard conversion method for clinically equivalent doses, the equivalent dose in mice was 1.2 g/kg, which was set as the low-dose WDD group (WDD-L). The high-dose WDD group (WDD-H) received 2.4 g/kg. Based on a mouse gavage volume of 0.2 mL/10 g, the concentrations for the WDD-L and WDD-H groups were 0.06 and 0.12 g/mL, respectively. Therefore, before gavage, one packet of WDD formula granules (3.2 g) was dissolved in 53 mL of purified water for the WDD-L group, and another packet was dissolved in 26 mL of purified water for the WDD-H group.

### Preparation of rosiglitazone

The human dose of rosiglitazone capsules was 30 mg/day, equivalent to 0.5 mg/kg. After conversion to the clinically equivalent dose in mice, this corresponded to 6.2 mg/kg. Using the same gavage volume of 0.2 mL/10 g, the contents of the rosiglitazone capsules were diluted to a final concentration of 0.31 mg/mL. Specifically, the contents of one rosiglitazone hydrochloride capsule (15 mg) were dissolved in 48 mL of purified water.

### Construction of the growth curve for *Parasutterella excrementihominis*

*Parasutterella excrementihominis* was purchased from HZBio, Microbial Conservation (JCM#15078), and cultured in DSMZ Medium 110 containing chopped beef particles, ferriheme chloride, vitamin K1, and L-cysteine hydrochloride solution. The absorbance at OD_600_ was measured at different time points during bacterial growth. After bacterial growth reached its peak, the spread plate method was used for colony counting to facilitate subsequent quantification during monoclonal transfer.

### Tolerance test of *P. excrementihominis* in simulated gastrointestinal fluid

First, the bacterial count at the logarithmic growth phase was determined using the spread plate method. The bacterial suspension was then centrifuged at 4 °C and 8000 rpm for 15 min. After the culture medium was discarded, simulated gastric fluid (pH 3.0) was added, followed by incubation for 2 h. Colony-forming units (CFU) were then determined using the spread plate method. After the simulated gastric fluid was discarded, centrifugation was again performed at 4 °C and 8000 rpm for 15 min. Simulated intestinal fluid containing trypsin and phosphate (pH 6.8) was then added, and bacterial CFU were measured after 2 and 6 h of incubation.

### Establishment of the MAFLD mouse model and treatment with WDD/7α-OH-T

Specific pathogen-free male C57BL/6 J mice aged 6 weeks were provided by Jiangsu Qinglongshan Biological Technology Co., Ltd. [certificate no. SCXK (SU) 2024–0001]. The mice were housed in the Animal Experiment Center of Nanjing University of Chinese Medicine. All animal procedures were conducted in accordance with the relevant ethical regulations (ethical approval no. 202502A001). Fifty mice were randomly divided into the control, model, WDD-L, WDD-H, and rosiglitazone groups. Similarly, 30 mice were randomly divided into the control, model, 7α-OH-T (5 mg/kg), 7α-OH-T (10 mg/kg) [10], and rosiglitazone groups. The control group was fed a methionine- and choline-sufficient purified diet, whereas the other groups were fed a choline-deficient, L-amino acid-defined HFD for 5 weeks. From the start of model establishment, the WDD-L, WDD-H, 7α-OH-T, and rosiglitazone groups were administered the corresponding treatments until the end of the experiment.

### Transplantation experiment of *P. excrementihominis* in MAFLD mice

Specific pathogen-free male C57BL/6 J mice aged 6 weeks were provided by Jiangsu Qinglongshan Biological Technology Co., Ltd. [certificate no. SCXK (SU) 2024–0001]. The mice were housed in the Animal Experiment Center of Nanjing University of Chinese Medicine. All animal procedures were conducted in accordance with the relevant ethical regulations (ethical approval no. 202507A001). Thirty-two mice were randomly divided into the MAFLD group, antibiotic-pretreated MAFLD group (ABX&MAFLD), *P. excrementihominis* group (PE), and antibiotic-pretreated *P. excrementihominis* group (ABX&PE). The ABX&MAFLD and ABX&PE groups first received a quadruple antibiotic cocktail in drinking water for 4 weeks to deplete the existing host gut microbiota. The cocktail contained 1 g/L metronidazole, 1 g/L ampicillin sodium, 1 g/L neomycin sulfate, and 0.5 g/L vancomycin hydrochloride. Subsequently, all groups were fed a choline-deficient, L-amino acid-defined HFD for 4 weeks to establish the MAFLD model. The PE and ABX&PE groups concurrently received daily gavage with *P. excrementihominis* (5 × 10^8^ CFU).

#### Fecal DNA extraction and bacterial quantification by qPCR [11]

Cecal contents (0.1 g) were weighed, and total DNA was extracted using a DNA extraction kit (DC301-0, Vazyme). DNA concentration and purity were determined using a NanoDrop spectrophotometer. Absolute quantification of bacterial DNA in cecal contents was then performed using the Femto™ Bacterial DNA Quantification Kit (Zymo Research, Cat. No. E2006). The kit contained a bacteria-specific qPCR master mix, including 16S rRNA gene primers and SYTO9 fluorescent dye, and eight vials of tenfold serially diluted *Escherichia coli* JM109 genomic DNA standards, with DNA inputs per reaction of 20 ng, 2 ng, 0.2 ng, 0.02 ng, 0.002 ng, 0.0002 ng, and 0.00002 ng, respectively. The qPCR reaction system consisted of 18 μL of Femto™ Bacterial qPCR Premix and 2 μL of DNA in a total volume of 20 μL. Cecal content samples were appropriately diluted to ensure that the Ct values fell within the range of the standard curve. Six replicate wells were used for each sample.

For standard curve construction and quantification, a linear regression curve (R^2^ = 0.998) was generated using the common logarithm (log_10_) of the DNA input per reaction (ng) for the standards as the x-axis and the corresponding Ct values as the y-axis. The Ct values of the test samples were substituted into the standard curve to calculate the amount of bacterial DNA in each sample well. The bacterial DNA content per gram of cecal content (μg/g feces) was then calculated according to the dilution factor of the sample DNA and the mass of cecal content used for extraction.

### Untargeted metabolomics of mouse cecal contents and liver

#### Sample extraction

Cecal contents or liver tissue (30 mg) were homogenized with 400 μL of methanol–water (4:1, v/v) using a tissue grinder. The mixture was subjected to ultrasonic extraction in an ice-water bath for 10 min, followed by overnight incubation at −40 °C. After centrifugation at 12,000 rpm and 4 °C for 20 min, the supernatant was collected and filtered through a 0.22-μm membrane. Metabolomic analysis was performed using a Waters ACQUITY UPLC I-Class Plus system coupled with a Thermo Q Exactive HF mass spectrometer. Separation was achieved on an ACQUITY UPLC HSS T3 column (100 mm × 2.1 mm, 1.8 μm) at a flow rate of 0.35 mL/min, with an injection volume of 3 μL. Mobile phase A was water containing 0.1% (v/v) formic acid, and mobile phase B was acetonitrile. The gradient elution profile was as follows: 5% B at 0.0–2.0 min, 5–30% B at 2.0–4.0 min, 30–50% B at 4.0–8.0 min, 50–80% B at 8.0–10.0 min, 80–100% B at 10.0–15.0 min, and 100–5% B at 15.0–16.0 min. Mass spectra were acquired by electrospray ionization (ESI) in both positive and negative modes using full MS/ddMS2 mode. The MS parameters were as follows: spray voltage, 3.8 kV in positive mode and 3.2 kV in negative mode; sheath gas, 35 Arb; auxiliary gas, 8 Arb; ion transfer tube temperature, 350 °C; resolution, 15,000; microscans, 1; AGC target, 2e5; and normalized collision energy, 20–60.

#### Data preprocessing and metabolite identification

Data preprocessing was performed before pattern recognition. Raw data were processed using XCMS v4.5.1 for baseline filtering, peak detection, integration, and retention time correction. Metabolites were identified by matching against the LuMet-Animal 3.0 LC–MS/MS database.

#### Data analysis

Univariate analysis using the t-test was performed to calculate statistical significance. Metabolites with VIP > 1, *P* < *0.05*, and fold change ≥ 2 or ≤ 0.5 were considered differentially abundant. Volcano plots were generated using ggplot2 in R to screen metabolites of interest based on log_2_(fold change) and −log_10_(*P* value). For clustering heatmaps, the intensity areas of differentially abundant metabolites were normalized using z scores and plotted using the pheatmap package in R. Correlations between differentially abundant metabolites were analyzed in R using Pearson correlation. The statistical significance of these correlations was calculated using cor.mtest in R. *P* < *0.05* was considered statistically significant. Correlation plots were generated using the corrplot package in R. The functions and metabolic pathways of these metabolites were analyzed using the KEGG database, and pathway enrichment analysis of differentially abundant metabolites was performed.

### 16S rRNA gene sequencing of mouse cecal contents

Fecal samples were collected from six randomly selected mice in each group, and the effects of WDD on intestinal microbial communities were assessed by 16S rRNA gene analysis. Sequencing was performed by OE Biotech Co., Ltd. (Shanghai, China). Total bacterial DNA was extracted using the E.Z.N.A.^®^ Soil DNA Kit (Omega Bio-Tek, Norcross, USA). The V3-V4 region of the 16S rRNA gene was amplified using the primers 343F (5′-TACGGRAGGCAGCAG-3′) and 798R (5′-AGGGTATCTAATCCT-3′). PCR was performed in a total volume of 20 μL containing 15 μL of ABI GeneAmp^®^ 9700 PCR Master Mix (ABI, Carlsbad, USA), 5 μmol/L forward and reverse primers, 0.2 μL of BSA, and 10 ng of template DNA. The PCR conditions were as follows: initial denaturation at 95 °C for 3 min; 27 cycles of 95 °C for 30 s, 55 °C for 30 s, and 72 °C for 45 s; and final extension at 72 °C for 10 min. PCR products were purified using the AxyPrep DNA Gel Extraction Kit (Axygen Biosciences, Union City, USA) and quantified using QuantiFluor™-ST (Promega Corporation, Madison, USA).

The sequencing data were submitted to the NCBI SRA database under accession number PRJNA1380366.

### Targeted BA metabolomic analysis

BA composition was analyzed using an AB Sciex QTRAP 5500 system coupled with a Waters I-Class system operating in positive and negative ion modes. The ion spray voltage was set at −4500 V and 5500 V. Gas 1 and Gas 2, both nitrogen, were maintained at 50 psi, and the collision gas pressure was 55 psi. The ion source temperature was 450 °C. Chromatographic separation was performed at 45 °C using a Phenomenex Kinetex C18 column (2.1 mm × 100 mm, 2.6 μm) at a flow rate of 0.45 mL/min. The mobile phases consisted of water with 0.1% formic acid (A) and acetonitrile with 0.1% formic acid (B). The gradient elution program was as follows: 0 min, A/B = 80:20 (v/v); 0.5 min, A/B = 80:20 (v/v); 1.5 min, A/B = 62:38 (v/v); 12 min, A/B = 50:50 (v/v); 17.5 min, A/B = 5:95 (v/v); 19 min, A/B = 5:95 (v/v); 19.01 min, A/B = 80:20 (v/v); and 20 min, A/B = 80:20 (v/v).

Principal component analysis (PCA) of BA profiles was performed using the MetaboAnalyst online platform. All statistical analyses and data visualization were performed using GraphPad Prism 8.0.

### Proteomics of mouse liver

After the experiment, liver tissue samples were placed on dry ice and sent to OE Biotech Co., Ltd. (Shanghai, China) for proteomic sequencing and analysis.

Liver tissue was ground in liquid nitrogen. After tissue lysis buffer was added, the samples were further ground using a frozen grinding instrument at −35 °C and 60 Hz for 120 s. The supernatant was collected after centrifugation, and protein concentration was determined using a BCA assay. Processed SP3 magnetic beads were added to the protein solution, followed by the addition of the corresponding volume of 100% acetonitrile. The mixture was incubated at room temperature for 20 min. After centrifugation, the supernatant was removed, and the magnetic beads were washed twice with the corresponding volumes of 70% ethanol and 70% acetonitrile. The wash supernatant was then removed. The magnetic beads were resuspended in 50 mM ammonium bicarbonate solution. DTT was added, followed by incubation at 55 °C for 30 min. CAA and trypsin were then added for enzymatic digestion at 37 °C with shaking at 1500 rpm.

The digested peptides were desalted using a SOLA™ SPE 96-well plate. Peptides were eluted three times with 150 μL of 50% acetonitrile–water containing 0.1% formic acid, yielding a total eluate volume of 450 μL, which was then dried under vacuum. Separation was achieved on a C_18_ column (2 cm × 75 μm, 3 μm) at a flow rate of 0.7 mL/min. Mobile phase A was an aqueous solution containing 0.1% formic acid, and mobile phase B consisted of 0.1% formic acid, 80% acetonitrile, and 20% water. The gradient elution profile was as follows: 7.5–35% B at 0.0–8.0 min and 35–100% B at 8.0–10.0 min. All mass spectrometry data files were merged using DIA-NN software for database searching of DIA mass spectrometry data and DIA-based protein quantification. The sequencing data were submitted to the iPROX system under accession number IPX0013493000.

### MAFLD THLE-2 model

THLE-2 cells were obtained from Wuhan Procell Biotechnology Co., Ltd. (CL-0833) and cultured at 37 °C with 5% CO_2_ in DMEM (KGL1206-500, KeyGEN BioTECH) supplemented with 10% fetal bovine serum (FBS) (209,111, NEST Biotechnology) and penicillin–streptomycin solution (BC-CE-007, BioChannel Biological Technology Co., Ltd.). Culture bottles and dishes (CCB06-025, CCB06-075, and CCD06-100A) were purchased from Bioland Biotechnology. THLE-2 cells were induced to establish a MAFLD model using a mixture of oleic acid and BSA, and lipid accumulation was visualized by Oil Red O staining.

### Histological hematoxylin–eosin and Oil Red O staining

Liver specimens were preserved in 4% paraformaldehyde and dehydrated through a graded alcohol series. The samples were then embedded in paraffin blocks, cut into 5-μm-thick sections, mounted on glass slides, and stained with hematoxylin–eosin for histopathological analysis. Another portion of liver tissue was frozen and cut into 8-μm-thick sections using a microtome. The sections were air-dried on slides and fixed in 10% formalin for 10 min. The slides were then rinsed with distilled water and soaked in 60% isopropanol. Staining was performed according to the instructions provided by Servicebio (Wuhan, China). Liver tissue sections stained with HE and Oil Red O were observed and photographed under a conventional optical microscope. Images of three randomly selected fields from each section were acquired and quantified using ImageJ software.

### Detection of biochemical indicators using an automatic biochemistry analyzer

Blood samples of approximately 0.5 mL were collected from the mouse orbit, left at room temperature for 1 h, and centrifuged at 3500 rpm and 4 °C for 10 min. The supernatant was collected. Biochemical indicators, including ALT, AST, LDL, HDL, and TG, were measured using the biochemistry module of the Roche integrated biochemistry-immunoassay system (cobas 8000, cobas ISE, cobas c701, and cobas c702).

### Glucose tolerance test and insulin tolerance test

After tail snipping, the first drop of blood was removed with tissue paper, and the second drop was applied to a blood glucose test strip to obtain a reading in mmol/L. Subsequently, glucose solution at 0.18 g/mL, prepared using 1.8 g glucose and 10 mL purified water, was administered by intraperitoneal injection at a dose of 0.1 mL per 10 g body weight. Blood glucose levels were then measured at 15, 30, 60, 90, and 120 min after injection, and the area under the curve (AUC, mmol/L·min) was calculated. Similarly, one week after the glucose tolerance test, an insulin tolerance test was performed. Based on an insulin concentration of 100 U/mL, the injection dose was set at 0.75 U/kg. The exact dose for a mouse weighing approximately 20 g was calculated as 0.015 U insulin. Blood glucose levels were measured at 15, 30, 60, 90, and 120 min after injection, and the AUC (mmol/L·min) was calculate [12].

### Western blot

Total protein was extracted using RIPA reagent and quantified with BCA assay kits (WB6501; New Cell & Molecular Biotech). Appropriate amounts of SDS loading buffer were added, and the samples were boiled. Proteins were separated by electrophoresis and electrotransferred onto PVDF membranes (SW120; Seven/Abcells, Beijing, China). The membranes were washed with TBST and blocked for 1 h, followed by incubation with primary antibodies. The secondary antibody was then added and incubated for 2 h. The bands were scanned using a Bio-Rad system, and relative protein levels were quantitatively analyzed.

### Molecular dynamics simulation

The docked complex was subjected to 100-ns all-atom molecular dynamics simulations using GROMACS (version 2023.2). The CHARMM36 force field was used for topology generation, energy minimization, and simulation of protein structures. The topology file for 7α-OH-T was generated using the CGenFF website. Energy minimization was performed using the steepest descent method until the maximum residual force was less than 1000 kJ/mol/nm. The system was equilibrated successively using 100-ps NVT ensembles (constant number of particles, volume, and temperature; V-rescale thermostat at 300 K) and 100-ps NPT ensembles (constant number of particles, pressure, and temperature; C-rescale barostat at 1 bar). Structural stability was evaluated by root-mean-square deviation (RMSD), time-dependent distance, and solvent-accessible surface area (SASA) analyses.

### Cellular thermal shift assay (CETSA)

Cell lysate (100 μL) was mixed with 7α-OH-T to achieve a final 7α-OH-T concentration of 5 μM. Control cell lysate was incubated with DMSO. The mixtures were incubated at different temperatures (42 °C, 45 °C, 48 °C, 51 °C, 54 °C, 57 °C, 60 °C, 63 °C, and 66 °C) for 3 min. Subsequently, all mixtures were centrifuged at 12,000 rpm for 10 min to obtain the supernatants, which were then analyzed for FXR expression by SDS-PAGE.

### Microscale thermophoresis (MST) assay

This experiment was performed with assistance from Bailaibo. MST was conducted using a Monolith NT.115 system. RED-NHS dye (7 μL) was thoroughly mixed with 7 μL of NHS labeling buffer to obtain a dye solution at a final concentration of 300 μM. An aliquot of 10 μL of this solution was then combined with 90 μL of 10 μM FXR. After protein labeling, 100 μL of the dye-protein labeling reaction mixture was loaded onto a pre-equilibrated desalting column, and the flow-through containing the labeled protein was collected. A 10 μL aliquot of the fluorescently labeled protein was diluted threefold with assay buffer before instrumental analysis.

### Statistical analysis

The results are expressed as the mean ± standard deviation (mean ± SD). Comparisons between two groups were performed using Student’s t-test, whereas comparisons among multiple groups were assessed by analysis of variance (ANOVA) using GraphPad Prism 8.0. *P* values are indicated in the figures as follows: not significant (ns), ^*^P < 0.05, ^**^P < 0.01, and ^***^P < 0.001.

## Results

### Reversal of hepatic steatosis and intestinal mucosal barrier injury by WDD in MAFLD mice depended on the integrity of the host microbiota

**Fig. 1:**
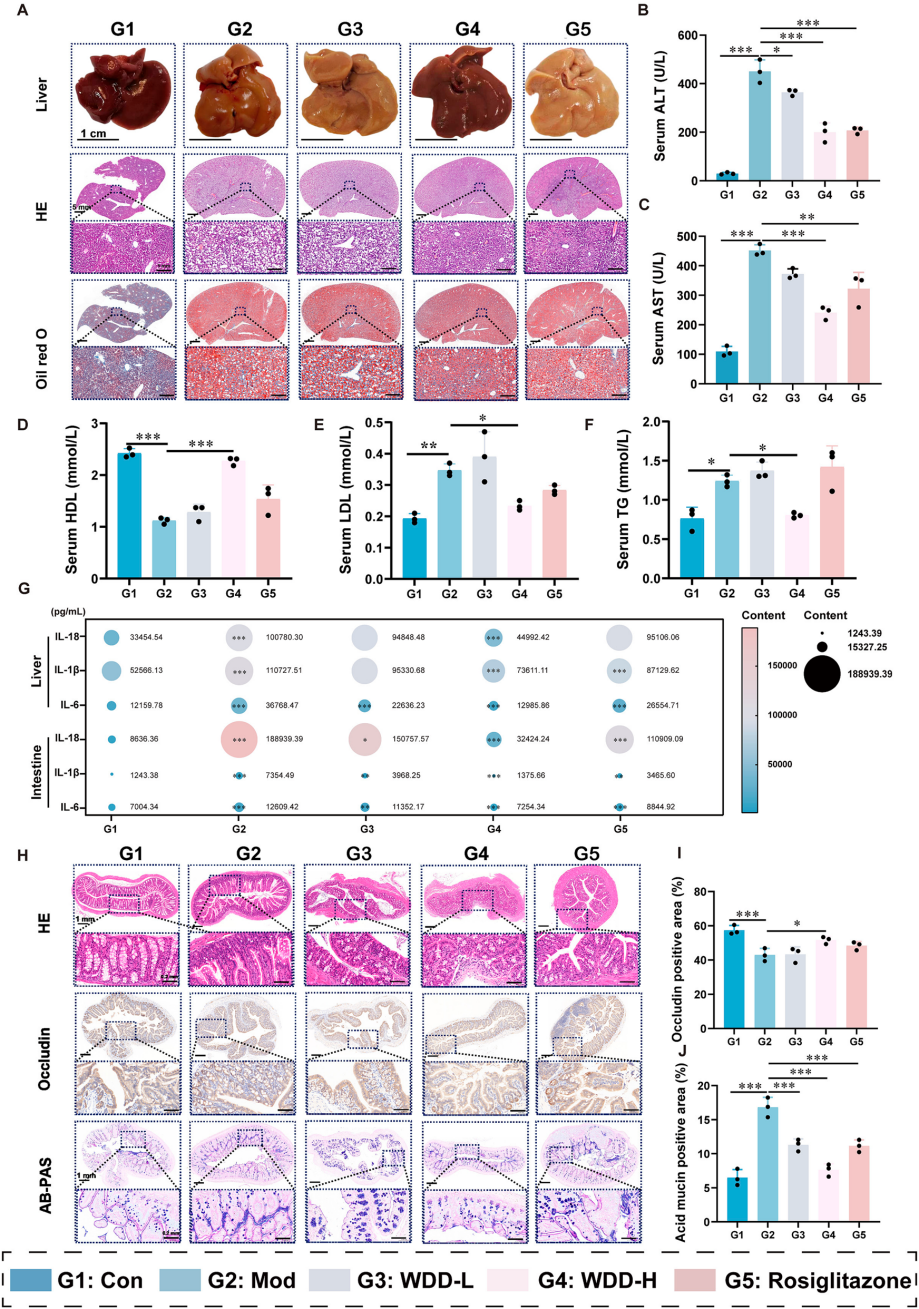
WDD alleviated hepatic steatosis and intestinal mucosal barrier injury in MAFLD mice. **A** Gross Appearance (scale bar = 1 cm), representative images of MAFLD mouse liver stained with HE and Oil Red O (scale bar = 5 mm). **B**, **C** Serological detection of liver injury indicators. **D**–**F** Serological testing of blood lipid related indicators. **G** ELISA detection of inflammation related indicators of IL-6, IL-1β and IL-18. Note: A dot represented the average value of 6 wells repeat. **H** HE staining immunohistochemistry of Occludin, and AB-PAS staining of colon tissue (scale bar = 1 mm). **I** Semi quantitative analysis of Occludin. **J** Semi quantitative analysis of Acid mucin. Data were presented as mean ± SD. ^*^P < 0.05, ^**^P < 0.01, ^***^P < *0.001* represented significance

After HFD induction, the livers of mice in the model group appeared brown and pale. In contrast, the high-dose WDD group (WDD-H) showed reddish livers that closely resembled the normal phenotype (Fig. 1A). Neither low-dose WDD (WDD-L) nor rosiglitazone markedly improved the gross appearance of the liver in MAFLD mice. H&E staining revealed pronounced inflammatory infiltration and ballooning degeneration of hepatocytes in the model group. Both WDD-H and rosiglitazone effectively alleviated tissue inflammation and hepatocellular structural damage. Oil Red O staining showed severe lipid deposition involving more than 60% of the liver tissue in model mice (Fig. 1A and Fig. S2A; *P* < *0.001*). WDD-H and rosiglitazone effectively reduced lipid deposition (*P* < *0.01*). Serum biochemical analysis showed that the liver injury markers ALT and AST were significantly elevated in model mice (Fig. 1B, C; *P* < *0.001*), whereas WDD-H and rosiglitazone significantly ameliorated liver injury (*P* < *0.01*). Lipid profile analysis showed decreased high-density lipoprotein (HDL) levels and increased low-density lipoprotein (LDL) levels in the model group (Fig. 1D, E; *P* < *0.001, P* < *0.01*). Triglyceride (TG) levels were also elevated in model mice (Fig. 1F; *P* < *0.05*). Only WDD-H significantly improved all three lipid parameters (*P* < *0.001, P* < *0.05, P* < *0.05*). Hepatic IL-6, IL-1β, and IL-18 levels were increased by at least twofold in model mice (Fig. 1G; *P* < *0.001*). WDD-H treatment generally reduced the levels of these cytokines by more than 30% (*P* < *0.001*). An antibiotic-mediated microbiota depletion (ABX) experiment was also conducted (Fig. S2C). In the absence of ABX, WDD markedly alleviated inflammation and reduced the area of lipid deposition by more than 50%. However, after ABX treatment, extensive inflammatory infiltration persisted, and the area of lipid deposition was twice that observed with WDD treatment alone (Fig. S2D-E; *P* < *0.001*).

The effects of long-term HFD feeding on the gastrointestinal tract of MAFLD mice were also evaluated. HFD significantly increased IL-6, IL-1β, and IL-18 levels in intestinal tissues (Fig. 1G; *P* < *0.001*). H&E staining confirmed severe lymphocyte aggregation in the colonic tissues of model mice (Fig. 1H), which was effectively alleviated by WDD treatment. Occludin immunohistochemistry showed that HFD caused the loss of tight junction proteins in the colon (Fig. 1H, I; *P* < *0.001*). AB-PAS staining further revealed an imbalance in acid mucin secretion in the colon (Fig. 1H and J; *P* < *0.001*). WDD-H improved tight junction integrity in the intestinal wall (*P* < *0.05*) and reduced intestinal mucus secretion by approximately 50% (*P* < *0.001*). These results suggested that WDD alleviated HFD-induced intestinal barrier injury and inflammation. Similarly, after ABX treatment, the ability of WDD to restore the occludin-positive area was reduced by 30% (Fig. S2F–G; *P* < *0.05*). Together, these findings indicated that WDD directly ameliorated hepatic lipid accumulation and intestinal mucosal barrier injury and that its efficacy also depended on its effects on the host gut microbiota.

### WDD remodeled intestinal lipid, cholesterol, and BA metabolism in MAFLD mice

**Fig. 2:**
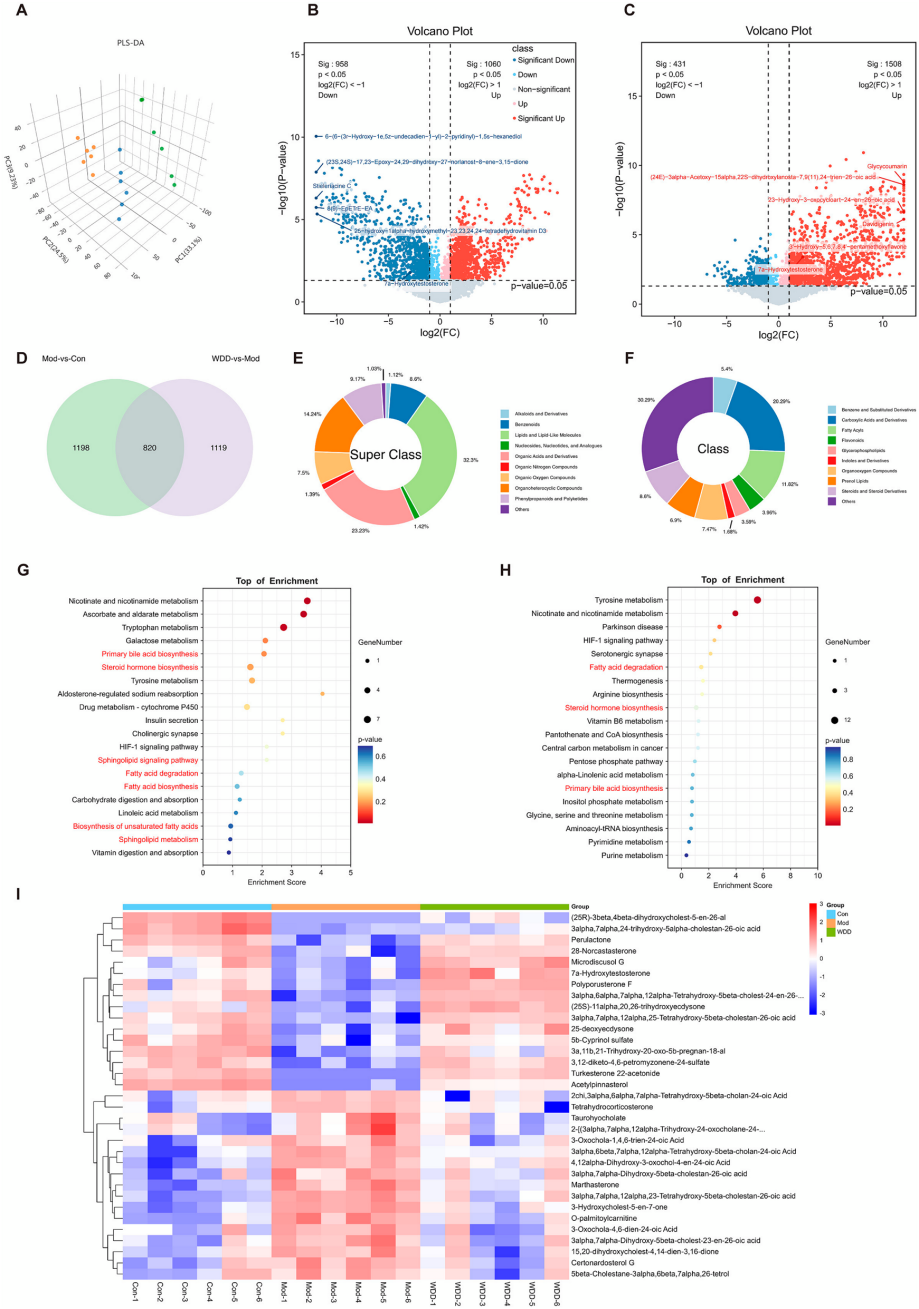
WDD remodeled intestinal lipid, cholesterol, and BA metabolism in MAFLD mice. **A** PLS-DA (3D). **B**, **C** Differential metabolites displayed in volcano maps. **D** Differential metabolites displayed in the Venn diagram. **E**, **F** Metabolites displayed by Pie Class. **G**, **H** KEGG pathway enrichment. **I** Heat map displayed the differential metabolites of interest

HFD not only directly disrupted the intestinal barrier but also altered the host microbiota and promoted the enrichment of opportunistic pathogens. These pathogens may induce metabolic inflammation, further aggravate barrier injury, and impair liver function through the gut-liver axis, thereby creating a vicious cycle. Because the cecum is a major site of microbial colonization and metabolism, the metabolic profile of cecal contents can reflect the functional status of the microbiota and its interactions with the host. Untargeted metabolomic analysis of cecal contents was therefore performed. PLS-DA revealed clear differences in intestinal metabolite profiles among the Con, Mod, and WDD groups (Fig. 2A). Compared with the Con group, the Mod group had 2,018 differential metabolites, including 1,060 upregulated and 958 downregulated metabolites. Compared with the Mod group, the WDD group had 1,939 differential metabolites, including 1,508 upregulated and 431 downregulated metabolites (Fig. 2B, C). A total of 820 differential metabolites were shared across the three groups (Fig. 2D). Hierarchical classification showed that Lipids and Lipid-like Molecules and Alkaloids and Derivatives were the predominant categories of differential metabolites (Fig. 2E). Further classification identified Fatty Acyls, Prenol Lipids, and Steroids and Steroid Derivatives as the major subclasses (Fig. 2F). KEGG enrichment analysis showed that pathways including Primary BA biosynthesis, Steroid hormone biosynthesis, and Fatty acid degradation were enriched in all group comparisons (Fig. 2G, H), suggesting that the therapeutic effects of WDD were closely associated with the regulation of these pathways. Detailed enrichment scores and other pathway parameters are provided in Table S3. Further analysis of key metabolites in these pathways revealed significant alterations in BA and fatty acid metabolites within the Steroids and Steroid Derivatives category (Fig. 2I).

**Fig. 3:**
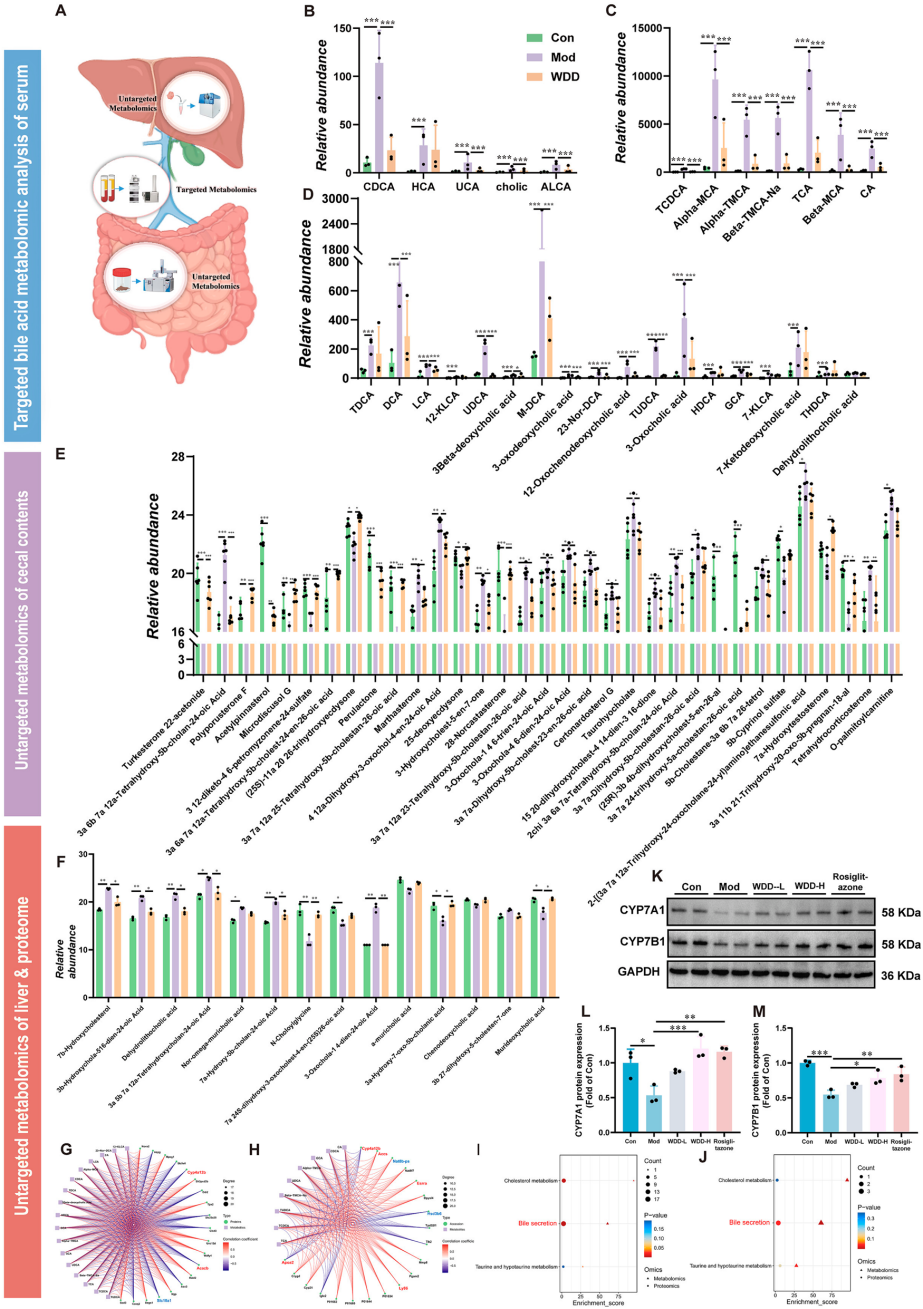
WDD reprogrammed the BA loop from liver → intent → blood. **A** Experimental flowchart. **B**, **C** Quantitative analysis of primary BAs in serum. **D** Quantitative analysis of secondary BAs in serum. **E** Relative quantitative analysis of BA-related metabolites in cecal contents. **F** Relative quantitative analysis of BA-related metabolites in the liver. **G**, **H** Combined analysis of liver proteomics and non-targeted metabolomics. **I**, **J** KEGG enrichment analysis. **K**–**M** WB detection of CYP7A1 and CYP7B1 expression. Data were presented as mean ± SD. ^*^P < 0.05, ^**^P < 0.01, ^***^P < *0.001* represented significance

Because BAs are key signaling molecules involved in lipid metabolism and BA-related pathways were enriched in the cecal metabolome, the effects of WDD on BA synthesis, metabolism, and circulation were further evaluated along the liver-intestine-blood axis (Fig. 3A). Targeted serum metabolomics showed that MAFLD mice had a significantly increased total serum BA burden, accompanied by markedly elevated levels of several toxic secondary BAs, including LCA and DCA (Fig. 3B–D). WDD significantly reduced both the total serum BA burden and toxic BA levels, with reductions exceeding 80% for eight BAs. Untargeted metabolomic analyses of cecal contents and liver tissue further showed that WDD increased the levels of beneficial alternative-pathway metabolites, including CDCA and TCDCA (Fig. 3E, F). In the liver, the restoration of several primary and secondary BAs toward normal levels was associated with key enzymes involved in fatty acid metabolism and BA synthesis or metabolism, including Cyp4a12b, Acacb, Slc10a1, Esrra, Ly86, and Hsd3b6 (Fig. 3G, H). KEGG enrichment analysis further confirmed that WDD modulated bile secretion (Fig. 3I, J). Given the marked enrichment of the bile secretion pathway, the expression of CYP7A1, a key enzyme in the classical BA synthesis pathway, and CYP7B1, a key enzyme in the alternative pathway, was examined. In the livers of Mod mice, the expression levels of both enzymes were approximately half those observed in normal mice (Fig. 3K–M; *P* < *0.05, P* < *0.001*), suggesting impaired BA synthesis. WDD-H partially restored the expression of these enzymes, thereby improving BA circulation (*P* < *0.001, P* < *0.05*).

These results suggested that WDD modulated the microbial metabolism of toxic secondary BAs and exerted systemic regulatory effects along the host microbiota-BA metabolism-lipid homeostasis axis by remodeling lipid and sterol metabolism. This finding may partly explain why the therapeutic efficacy of WDD was significantly attenuated after depletion of the host microbiota.

### WDD regulated gut microbiota diversity and composition in MAFLD mice

**Fig. 4:**
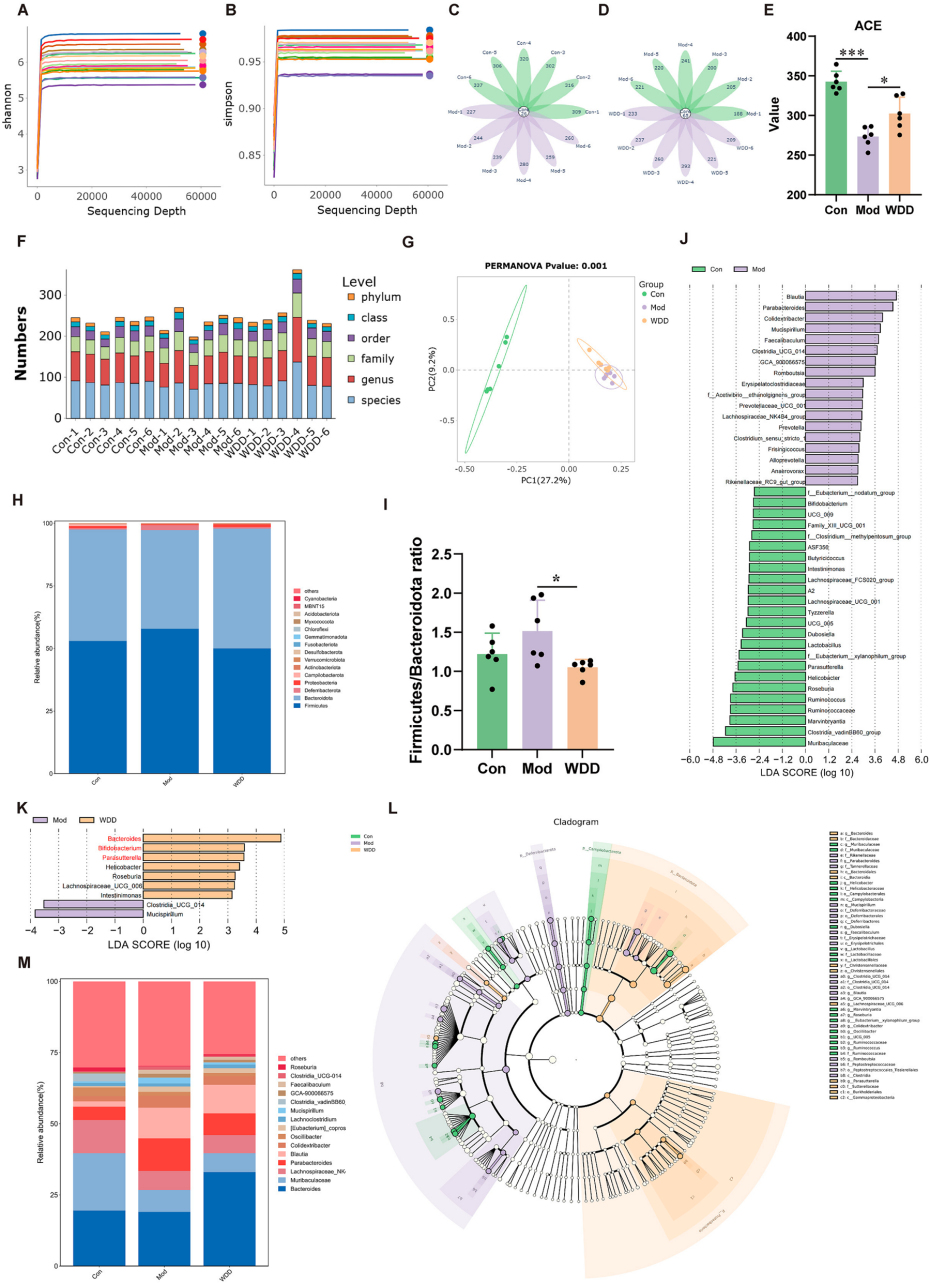
WDD regulated the diversity and proportion of gut microbiota in MAFLD mice. **A**, **B** Shannon and Simpson indices rarefaction curves. **C**, **D** ASVs displayed in petal chart. **E** Barplot of alpha diversity ACE values. **F** Community levelbarplot. **G** PCoA (2D). **H** Barplot of sample community structure. **I** *Firmicutes/Bacteroidota* ratio. **J**, **K** Differential species score chart. **L** Example diagram of differential species annotation branch. **M** Barplot of sample community structure. Data were presented as mean ± SD (6 mice per group). ^*^P < 0.05, ^***^P < *0.001* represented significance

The observed remodeling of BA and lipid metabolism suggested that WDD exerted its therapeutic effects by modulating the host microbiota. To identify the key gut microorganisms involved in this process, 16S rRNA gene sequencing was performed. Rarefaction curves based on the Shannon and Simpson indices indicated that the sequencing depth was sufficient (Fig. 4A, B). Analysis of amplicon sequence variants (ASVs) showed that the number of ASVs unique to the Mod group was significantly lower than that in the Con group, although the two groups shared 26 ASVs (Fig. 4C). After WDD treatment, the numbers of both shared and unique ASVs increased significantly (Fig. 4D). Alpha diversity analysis based on the abundance-based coverage estimator (ACE) showed a reduction of more than 20% in MAFLD mice compared with the Con group. WDD treatment increased the ACE value, indicating improved alpha diversity (Fig. 4E). These differences were also evident in the community composition bar plots (Fig. 4F). Principal coordinate analysis (PCoA) based on Jaccard distance showed that WDD partially corrected the altered gut microbiota structure in the Mod group (Fig. 4G). At the phylum level, this shift was reflected by changes in the relative abundances of *Firmicutes*, *Bacteroidota*, *Deferribacterota*, and *Proteobacteria* following WDD treatment (Fig. 4H). A high *Firmicutes/Bacteroidota* ratio is commonly associated with chronic inflammation and obesity. WDD significantly reduced this ratio (Fig. 4I), consistent with its observed anti-inflammatory and lipid-lowering effects. Microbial alterations at the genus level were further examined. Linear discriminant analysis effect size (LEfSe) analysis revealed enrichment of genera such as *Blautia* and *Parabacteroides* in the model group. After WDD treatment, *Bacteroides*, a genus within *Bacteroidota*, and *Parasutterella*, a genus within *Proteobacteria*, became predominant (Fig. 4J–L), suggesting that they may contribute to disease amelioration. Quantitative analysis at the genus level further confirmed that *Bacteroides* and *Parasutterella* were the predominant taxa responsive to WDD treatment (Fig. 4M).

In summary, 16S rRNA gene sequencing showed that WDD improved gut microbiota diversity in MAFLD mice and regulated the balance between beneficial and pathogenic bacteria.

### Integrative multi-omics analysis identified *Parasutterella* and 7α-OH-T as key gut-derived candidates associated with WDD-induced MAFLD improvement

**Fig. 5:**
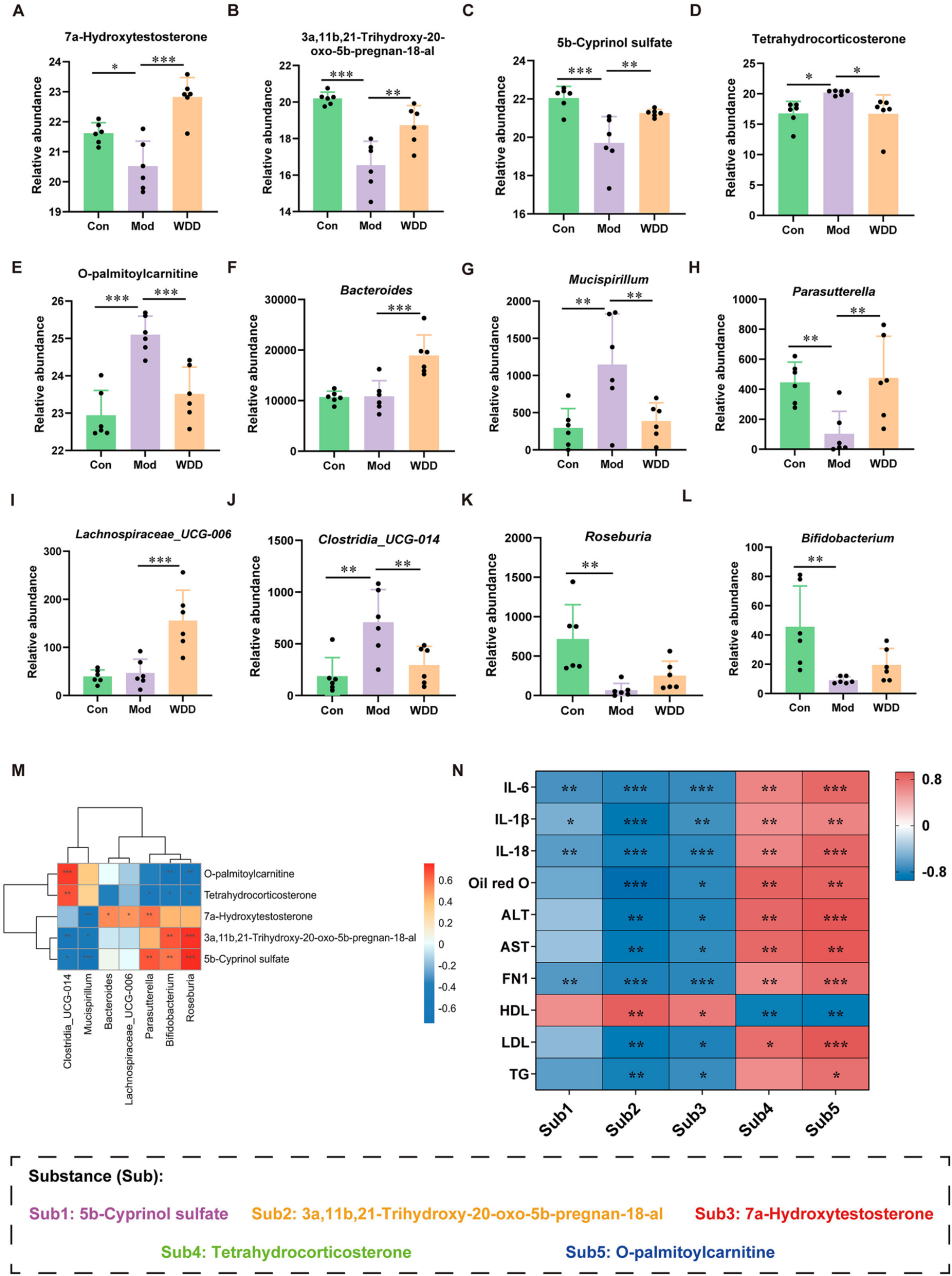
Integrative multi-omics analysis identified *Parasutterella* and 7α-OH-T as key gut-derived candidates associated with WDD-induced MAFLD improvement. **A**–**E** Relative quantitative analysis of differential metabolites. **F**–**L** Relative quantitative analysis of gut microbiota regulating differential metabolites. **M** Correlation analysis between gut microbiota and differential metabolites. **N** *Pearson* correlation analysis between differential metabolites and pharmacological indicators. Data were presented as mean ± SD (6 mice per group). ^*^P < 0.05, ^**^P < 0.01, ^***^P < *0.001* represented significance

To further characterize the relationships between the host microbiota and microbial metabolites, an integrative correlation analysis of untargeted metabolomic and 16S rRNA gene sequencing data from cecal contents was performed. First, the top five fatty acid- or sterol-related metabolites that differed significantly in both the Mod and WDD groups were identified, including 7α-hydroxytestosterone (7α-OH-T), 3α,11β,21-trihydroxy-20-oxo-5β-pregnan-18-al, and 5β-cyprinol (Fig. 5A–E). Seven bacterial genera strongly correlated with these metabolites were subsequently identified, including *Bacteroides*, Mucispirillum, and *Parasutterella* (Fig. 5F–L). Based on its decrease in the model group and increase after WDD treatment, *Parasutterella* was identified as a candidate genus with potential hepatoprotective and lipid-lowering effects. Correlation analysis showed that the abundance of *Parasutterella* was positively correlated with the levels of 7α-OH-T and 5β-cyprinol (Fig. 5M; *P* < *0.05*). Further *Pearson* correlation analysis between these metabolites and indicators of hepatoprotective and lipid-lowering efficacy showed that, of 7α-OH-T and 5β-cyprinol, only 7α-OH-T was negatively correlated with inflammatory, fibrotic, and blood lipid indices (Fig. 5N). These findings suggested that 7α-OH-T was closely associated with *Parasutterella* and had potential hepatoprotective and lipid-lowering effects.

Based on these results, three lines of evidence were identified: (1) 7α-OH-T was consistently identified as a differential metabolite in the steroid hormone biosynthesis pathway in both the Mod and WDD comparisons, and its abundance pattern suggested a potentially beneficial role; (2) 7α-OH-T was strongly and positively correlated with the abundance of the beneficial genus *Parasutterella*; and (3) Pearson correlation analysis showed that 7α-OH-T was negatively correlated with multiple adverse disease indicators. Together, these findings supported 7α-OH-T as a promising candidate metabolite, and it was therefore selected for further study.

### WDD restored hepatic metabolic homeostasis by coordinating steroid catabolism, BA synthesis, and FAO in MAFLD

**Fig. 6:**
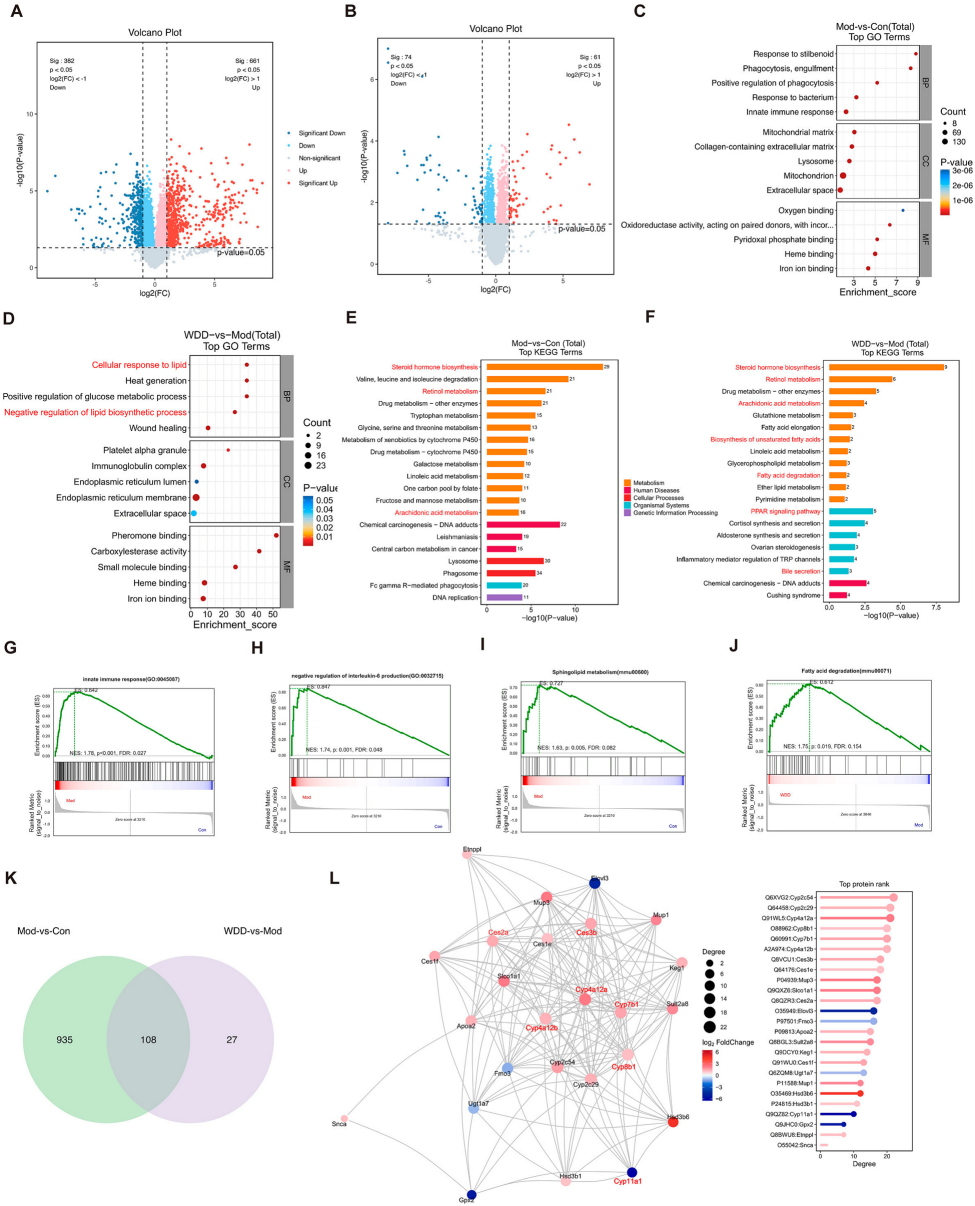
WDD restored hepatic metabolic homeostasis by coordinating steroid catabolism, BA synthesis, and FAO in MAFLD. **A**, **B** Differentially expressed proteins displayed in volcano maps. **C**, **D** GO enrichment. **E**, **F** KEGG enrichment. **G**–**J** GSEA analysis of key pathways. **K** Differentially expressed proteins displayed in the Venn diagram. **L** PPI analysis of differentially expressed proteins

Proteomic analysis of liver tissue was performed to investigate the intrahepatic mechanisms by which WDD ameliorated MAFLD. A total of 1,043 and 135 differentially expressed proteins were identified in the Mod versus Con and WDD versus Mod comparisons, respectively (Fig. 6A, B). GO enrichment analysis showed that “Cellular response to lipid” and “Negative regulation of lipid biosynthetic process” were key biological processes associated with the WDD-mediated reduction in lipid deposition (Fig. 6C, D). KEGG pathway analysis further showed that steroid hormone biosynthesis, arachidonic acid metabolism, and retinol metabolism were prominent pathways in the model group (Fig. 6E). In the WDD group, lipid metabolism-related pathways, including steroid hormone biosynthesis, retinol metabolism, arachidonic acid metabolism, fatty acid degradation, and the PPAR signaling pathway, as well as the BA metabolism-related bile secretion pathway, were significantly enriched (Fig. 6F). These findings further supported the multifaceted mechanism by which WDD ameliorated MAFLD. GSEA showed that the pathways significantly upregulated in the model group were primarily associated with the innate immune response, negative regulation of interleukin-6 production, and sphingolipid metabolism (Fig. 6G–I). By contrast, the fatty acid degradation pathway was significantly upregulated after WDD treatment (Fig. 6J).

A PPI network constructed from 108 differentially expressed proteins further illustrated the interactions within the BA-lipid metabolism regulatory network (Fig. 6K). CYP4A12A and CYP4A12B, which regulate fatty acid metabolism, occupied central positions in the network (Fig. 6L) and were suppressed in model mice (Fig. S3A-B; *P* < *0.001*). CYP7B1 and CYP8B1, key enzymes in the alternative and classical BA synthesis pathways, respectively, which normally function in a complementary manner, were also downregulated in model mice (Fig. 6L and Fig. S3C-D; *P* < *0.001*). These results indicated that the conventional BA synthesis-secretion-lipolysis process was disrupted in MAFLD mice. In addition to increasing the expression of CYP4A12A/CYP4A12B and CYP7B1/CYP8B1 (*P* < *0.01*), WDD downregulated CYP11A1, the rate-limiting enzyme in steroid synthesis, and upregulated CES2A and CEA3B expression (Fig. S3E-G; *P* < *0.001*). These findings suggested that WDD restored BA synthesis-secretion-lipolysis homeostasis by inhibiting steroid synthesis and promoting cholesterol hydrolysis, thereby providing precursors for BA synthesis.

In summary, the intrahepatic mechanism by which WDD ameliorated MAFLD was closely associated with enhanced FAO, regulation of BA synthesis and transport, and suppression of inflammatory responses.

### *Parasutterella* transplantation confirmed its causal role in remodeling hepatic lipid metabolism via the gut-liver axis

**Fig. 7:**
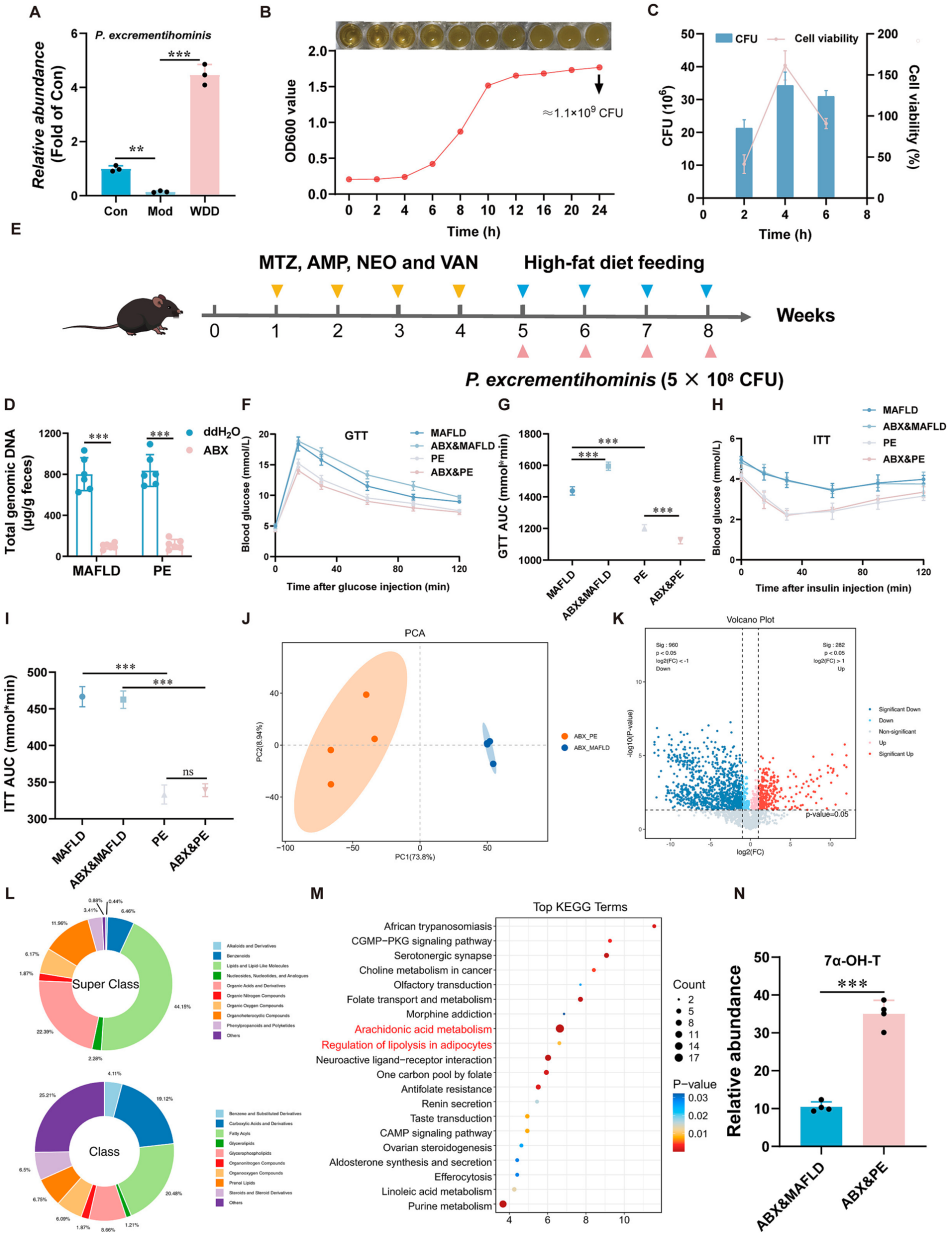
*Parasutterella* transplantation confirmed its causal role in remodeling hepatic lipid metabolism via the gut-liver axis. **A** Relative quantitative analysis of *P. excrementihominis* abundance after oral gavage of WDD. **B** Growth curve of *P. excrementihominis*. **C** Stability test in artificial gastrointestinal fluid. **D** qPCR detection of host microbiota depletion. **E** Flowchart for the treatment of MAFLD mice with *P. excrementihominis*. **F**, **G** Glucose tolerance test. **H**, **I** Insulin tolerance test. **J** PCA. **K** Differential metabolites displayed in volcano maps. **L** Metabolites displayed by Pie Class. **M** KEGG pathway enrichment. **N** Relative quantitative analysis of 7α-OH-T. Data were presented as mean ± SD. ^**^P < *0.01*, ^***^P < *0.001* represented significance

The results presented in Sects.  3.4 and 3.5 showed that WDD concurrently reshaped the gut microbial community, most notably by enriching *Parasutterella*, and modulated hepatic BA synthesis and fatty acid metabolism pathways. However, whether gut-derived metabolites acted through the gut-liver axis to coordinate with intrahepatic mechanisms and exert hepatoprotective and lipid-lowering effects remained unclear. Single-bacterium transplantation was therefore performed to investigate the causal relationships among *Parasutterella*, the metabolite 7α-OH-T, and these intrahepatic pathways. The representative species *P. excrementihominis* (JCM#15078) was selected for transplantation because it has previously been reported to have lipid-lowering and anti-obesity potential [13]. The relative abundance of *P. excrementihominis* in cecal contents was initially measured, confirming that WDD promoted its growth (Fig. 7A; *P* < *0.001*). A growth curve was then established for *P. excrementihominis*, showing that the bacterium approached the stationary phase after 12 h in broth medium (Fig. 7B) and reached a concentration of 1.1 × 10^9^ CFU after 24 h, meeting the threshold required for single-bacterium transplantation. Because tolerance to gastrointestinal fluids is essential for bacterial survival in the gastrointestinal tract, *P. excrementihominis* was tested in artificial gastrointestinal fluids. Its survival rate was 41% after 2 h in artificial gastric fluid, whereas the survival rates in artificial intestinal fluid were 162% after 2 h and 91% after 4 h (Fig. 7C). Before transplantation, the gut microbiota of mice in the ABX&MAFLD and ABX&PE groups was depleted using a four-antibiotic cocktail to minimize interference with *P. excrementihominis* colonization. Total bacterial DNA content was quantified after qPCR amplification and decreased by more than 85% after ABX treatment (Fig. 7D; *P* < *0.001*). Subsequently, mice in all remaining groups, including the ABX-treated groups, were fed an HFD for 4 weeks (Fig. 7E). After 4 weeks, glucose and insulin tolerance tests were performed to evaluate the effects of *P. excrementihominis* on MAFLD. The PE group exhibited better glucose tolerance and lower AUC values than the MAFLD group, whereas the ABX&PE group exhibited better glucose tolerance and lower AUC values than the ABX&MAFLD group (Fig. 7F–G; *P* < *0.001*). Similarly, after intraperitoneal insulin injection, mice in both the PE and ABX&PE groups showed greater insulin sensitivity and lower AUC values (Fig. 7H, I; *P* < *0.001*).

Untargeted metabolomic analysis was performed on liver tissues from mice treated with *P. excrementihominis*. A distinct metabolic shift was observed between *P. excrementihominis*-treated and MAFLD mice (Fig. 7J). Among the 1,242 differential metabolites, Lipids and Lipid-like Molecules accounted for more than 40%, with Fatty Acyls representing the predominant lipid subclass (Fig. 7K, L). Enrichment analysis of differential metabolites showed that Arachidonic acid metabolism and Regulation of lipolysis in adipocytes were key pathways through which *P. excrementihominis* ameliorated MAFLD (Fig. 7M). Notably, hepatic 7α-OH-T levels increased after *P. excrementihominis* transplantation (Fig. 7N; *P* < *0.001*).

These results indicated that *Parasottella,* represented by *P. excrementihominis,* is a probiotic genus capable of stable intestinal colonization. Its transplantation increased beneficial compounds such as 7α-OH-T, which entered the liver through the gut-liver axis and reshaped hepatic lipid metabolism.

### *Parasutterella* ameliorated MAFLD by regulating hepatic lipid metabolism and intestinal barrier function

**Fig. 8:**
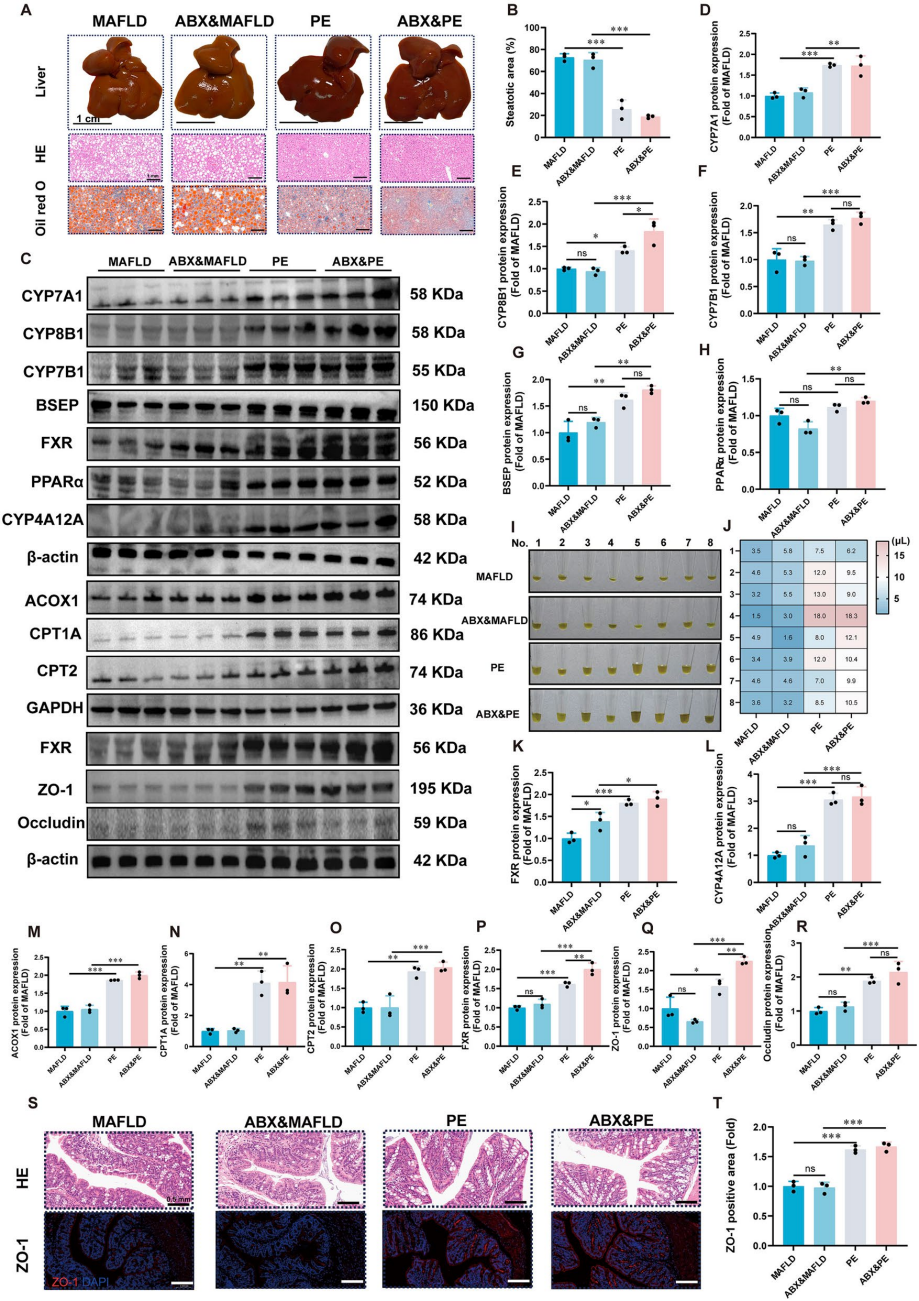
*Parasutterella* ameliorated MAFLD by regulating hepatic lipid metabolism and intestinal barrier function. **A** Gross Appearance (scale bar = 1 cm), representative images of MAFLD mouse liver stained with HE and Oil Red O (scale bar = 1 mm). **B** Semi quantitative analysis of Oil Red O staining. **C**–**H** and **K**–**R** WB detection of protein levels of CYP7A1/CYP8B1, CYP7B1/BSEP, FXR/PPARα/CYP4A12A, ACOX1, CPT1A, CPT2 and FXR/ZO-1/Occludin. **I**, **J** Gallbladder bile storage volume and heatmap. **S** HE staining and immunofluorescence of ZO-1 (scale bar = 0.5 mm). **T** Semi quantitative analysis of ZO-1. Data were presented as mean ± SD. ^*^P < 0.05, ^**^P < 0.01, ^***^P < *0.001* represented significance

To determine whether *Parasutterella* exerted hepatoprotective and lipid-lowering effects through the pathways identified by proteomic analysis, a series of pathological and molecular assays was performed. After *P. excrementihominis* transplantation, H&E and Oil Red O staining showed that the PE and ABX&PE groups exhibited less hepatocyte ballooning, reduced inflammatory infiltration, and a greater than 50% reduction in lipid accumulation (Fig. 8A, B; *P* < *0.001*). To elucidate the potential mechanisms underlying the therapeutic effects of *P. excrementihominis*, the expression of CYP7A1 and CYP8B1, which participate in the classical BA synthesis pathway; CYP7B1, which participates in the alternative pathway; and the bile salt export pump (BSEP) was examined to comprehensively assess the effects of *P. excrementihominis* on BA circulation. CYP7A1/CYP8B1 and CYP7B1/BSEP expression was suppressed in both the MAFLD and ABX&MAFLD groups (Fig. 8C–G), consistent with reduced BA reserves in the gallbladder (Fig. 8I, J). In contrast, *P. excrementihominis* enhanced both the classical and alternative BA synthesis pathways (Fig. 8C–G; *P* < *0.05*) and increased BA reserves (Fig. 8I, J). In parallel, the FXR/PPARα/CYP4A12A axis and the representative FAO-related proteins ACOX1, CPT1A, and CPT2 were examined to evaluate the effects of *P. excrementihominis* on FAO. The PE and ABX&PE groups exhibited approximately twofold increases in the activity of the FXR/PPARα/CYP4A12A axis and in the expression of ACOX1, CPT1A, and CPT2 (Fig. 8C, H–O; *P* < *0.05*).

Similarly, *P. excrementihominis* activated FXR and increased the abundance of ZO-1 and Occludin, thereby improving mucosal barrier function (Fig. 8C, P–R; *P* < *0.05*). H&E staining of the colon showed reduced inflammation in the PE and ABX&PE groups (Fig. 8S). Furthermore, ZO-1 expression was restored and increased by approximately 50% after *P. excrementihominis* treatment (Fig. 8S, T; *P* < *0.001*).

These results demonstrated that *P. excrementihominis* promoted BA metabolic homeostasis and fatty acid degradation by activating the hepatic FXR/PPARα/CYP4A12A axis and the classical and alternative BA synthesis pathways. In addition, by acting on colonic FXR, *P. excrementihominis* regulated ZO-1 and occludin expression and thereby improved intestinal barrier integrity. These findings suggest that *Parasutterella* has therapeutic potential for MAFLD.

### 7α-OH-T improved lipid deposition during MAFLD through FXR/PPARα/CYP4A12A

**Fig. 9:**
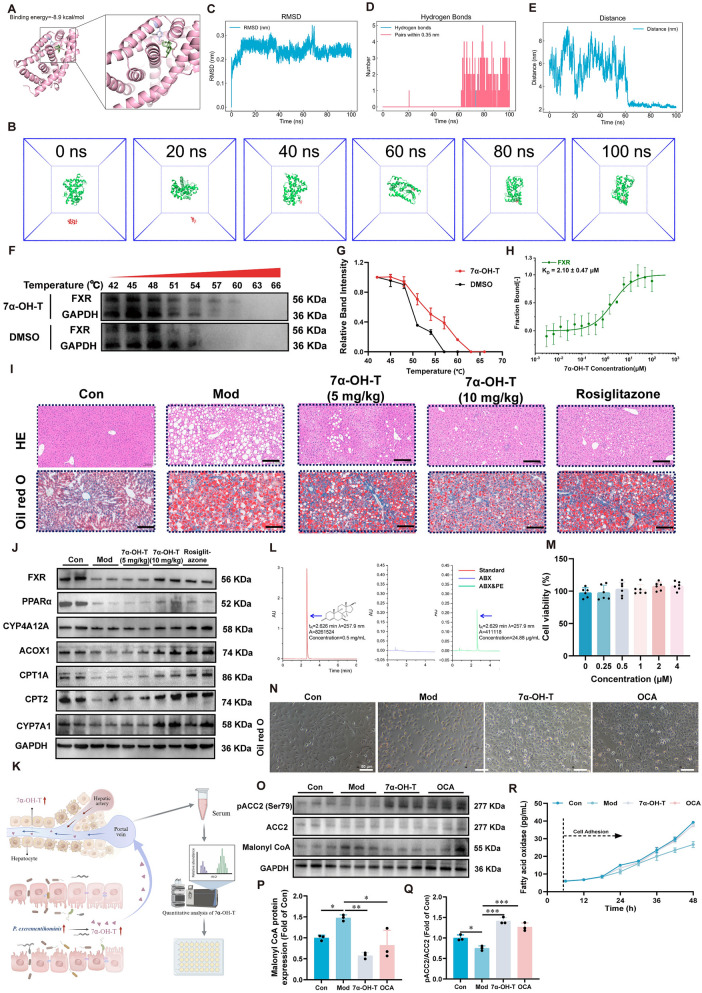
7α-OH-T improved lipid deposition during MAFLD through FXR/PPARα/CYP4A12A. **A** Display image of molecular docking. **B** Display diagram of molecular dynamics simulation. **C** RMSD. **D** Hydrogen bonds. **E** Time evolution of distance. **F**, **G** CETSA detection and protein degradation curve. **H** Detection of the binding ability between 7α-OH-T and FXR by MST. **I** Representative images of MAFLD mouse liver stained with HE and Oil Red O (scale bar = 1 mm). **J** WB detection of protein levels of FXR/PPARα/CYP4A12A, ACOX1, CPT1A and CPT2. **K** Schematic diagram of detecting portal vein 7α-OH-T. **L** UPLC/UV detecting portal vein 7α-OH-T. **M** CCK-8 detection of the effect of 7α-OH-T on cell viability. **N** Representative images of MAFLD THLE-2 stained with Oil Red O (scale bar = 50 μm). **O**–**Q** WB detection of ACC2 phosphorylation and Malonyl CoA expression. **R** ELISA detection of FAO. Data were presented as mean ± SD. ^*^P < 0.05, ^**^P < 0.01, ^***^P < *0.001* represented significance

After establishing that 7α-OH-T production was closely associated with *Parasutterella*, its hepatic lipid-lowering effects were further investigated. Because *P. excrementihominis* transplantation strongly regulated FXR-related pathways in both the liver and intestine, the potential targeting of FXR by 7α-OH-T was examined. Molecular docking showed that 7α-OH-T bound to FXR with a binding energy of −8.9 kcal/mol (Fig. 9A). Subsequent molecular dynamics simulations showed that 7α-OH-T formed a stable complex with FXR within 60 ns (Fig. 9B). RMSD fluctuations remained within 0.05 nm (Fig. 9C), and hydrogen bonds indicative of strong binding affinity were observed (Fig. 9D). After stable binding was achieved, the distance between 7α-OH-T and FXR remained within 3 nm (Fig. 9E). CETSA showed that 7α-OH-T binding increased the thermal stability of FXR from 51 °C to 60 °C (Fig. 9F–G). MST also showed that the *K*_*D*_ for binding was only 2.10 μM (Fig. 9H), indicating strong binding between 7α-OH-T and FXR.

To further evaluate its pharmacological activity, H&E and Oil Red O staining showed that 7α-OH-T administered at 10 mg/kg significantly ameliorated hepatic lipid accumulation (Fig. 9I). Examination of the FAO signaling axis and the classical BA synthesis pathway further showed that 7α-OH-T increased the expression of FXR/PPARα/CYP4A12A, ACOX1, CPT1A, CPT2, and CYP7A1 (Fig. 9J–M and Fig. S4A-G; *P* < *0.05*). Detection of 7α-OH-T in portal venous serum further supported its gut-derived identity and direct involvement in gut-liver transport (Fig. 9K). After *P. excrementihominis* transplantation following ABX treatment, the concentration of 7α-OH-T in portal venous serum reached approximately 24.88 μg/mL (Fig. 9L). To evaluate the activity of 7α-OH-T at the cellular level, previous evidence indicating an effective concentration of approximately 4 μM for testosterone-related compounds was considered [14]. This concentration was lower than the effective concentration converted from 24.88 μg/mL. The CCK-8 assay confirmed that 0.25–4 μM was a noncytotoxic concentration range (Fig. 9M). Within this range, 7α-OH-T reduced oleic acid-induced lipid droplet accumulation in THLE-2 cells, with an effect comparable to that of the FXR agonist obeticholic acid (Fig. 9N).

Mitochondrial proteins were extracted from THLE-2 cells, and the ACC2/malonyl-CoA axis was examined. Malonyl-CoA, the most potent physiological inhibitor of CPT1, is produced by mitochondria-localized active acetyl-CoA carboxylase 2 (ACC2) and strongly suppresses FAO. Following 7α-OH-T treatment, ACC2 phosphorylation at Ser79 increased, whereas malonyl-CoA levels decreased (Fig. 9O–Q; *P* < *0.01*), indicating that 7α-OH-T supplementation inhibited the synthesis of certain lipids. ELISA further showed that 7α-OH-T accelerated FAO (Fig. 9R), consistent with reduced malonyl-CoA synthesis and the consequent relief of CPT1A inhibition. Collectively, these results indicated that 7α-OH-T directly targeted hepatic FXR, promoted FAO through PPARα/CYP4A12A, ACOX1, CPT1A, and CPT2, and served as a key effector molecule mediating the lipid-lowering effects of this bacterium.

## Discussion

Disorders of lipid metabolism, aberrant BA signaling, inflammatory activation, and insulin resistance interact to form the complex metabolic pathophysiological network underlying MAFLD. However, current therapies largely target individual components of this network and are limited by interindividual variation in hepatic drug-metabolizing enzymes and adverse effects [15], which complicate effective intervention and long-term disease management. As an upstream regulator of host metabolism, the gut microbiota modulates hepatic lipid metabolism and inflammatory responses through the gut-liver axis [16]. Numerous studies have shown that probiotics, including *Lactobacillus delbrueckii subsp.* lactis LL001 [17] and *Bifidobacterium breve* CKDB002 [18], effectively alleviate MAFLD. Therefore, targeting the host microbiota and its metabolites represents a potential therapeutic strategy for MAFLD [19].

In this study, WDD ameliorated hepatic lipid deposition, inflammatory responses, and intestinal mucosal barrier injury in MAFLD mice when the host microbiota remained intact. Integrated metabolomic analysis of cecal contents and 16S rRNA gene sequencing showed that WDD significantly increased the levels of multiple lipid and sterol metabolites. Among these metabolites, 7α-OH-T showed the strongest inverse correlations with indicators of lipid accumulation and inflammation. WDD also enriched beneficial bacteria, including *Parasutterella*, whose abundance was significantly and positively correlated with 7α-OH-T levels. Based on these findings, we proposed that WDD ameliorates MAFLD by promoting the intestinal colonization of *Parasutterella* and increasing 7α-OH-T abundance.

To support this hypothesis, we reviewed previous studies on *Parasutterella*. This genus is a core member of the gut microbiota shared by humans and rodents and is involved in the regulation of host cholesterol metabolism. Animal studies have shown that HFD feeding significantly reduces *Parasutterella* abundance and that its abundance is inversely correlated with metabolic dysfunction phenotypes [20]. Human clinical trials have also demonstrated that increased intestinal *Parasutterella* abundance is positively associated with reduced serum LDL levels [21]. These findings are consistent with those of the present study and collectively suggest that *Parasutterella* contributes to improved host lipid metabolism.

To determine whether 7α-OH-T exerted therapeutic effects through the gut-liver axis, systematic mechanistic validation was performed. First, single-bacterium transplantation experiments confirmed that *P*. *excrementihominis*, a representative species of *Parasutterella*, modulated the classical and alternative BA synthesis pathways and the FXR/PPARα/CYP4A12A axis, thereby improving BA metabolism and lipid catabolism. Exogenous 7α-OH-T similarly alleviated hepatic lipid accumulation in MAFLD mice. We further demonstrated that 7α-OH-T bound strongly to FXR. Subsequent cellular experiments confirmed that 7α-OH-T activated the PPARα/CYP4A12A pathway and thereby promoted mitochondrial FAO. During our investigation of the effects of WDD on the classical and alternative BA synthesis pathways, an apparent paradox emerged: both CYP7A1 and CYP7B1 were downregulated in MAFLD mice rather than exhibiting the expected reciprocal expression pattern. In addition, elevated total serum BA levels contrasted with suppressed intrahepatic BA synthesis. This discrepancy may have resulted from severe hepatocyte injury, impaired BA synthesis, and leakage of intracellularly stored BAs. These observations warrant further mechanistic investigation.

Notably, the interaction between the steroid metabolite 7α-OH-T and FXR was not unprecedented. Previous studies have reported that androsterone, a testosterone metabolite, can bind directly to the ligand-binding domain of FXR and recruit the coactivator SRC-1 [22]. As a 7α-hydroxylated testosterone derivative, 7α-OH-T shares a common steroidal backbone with androsterone, and our molecular interaction findings were consistent with this previous observation. Moreover, FXR and PPARα, two key nuclear receptors governing lipid metabolism, exhibit complex cross-regulatory interactions and jointly integrate metabolic, immune, and gut microbial signals in MAFLD pathogenesis [23]. In this study, 7α-OH-T-mediated FXR activation was accompanied by enhanced activity of the PPARα/CYP4A12A pathway, providing a molecular basis for the coordinated effects of WDD through the microbiota-metabolite-nuclear receptor axis.

In summary, multi-omics analysis, 16S rRNA gene sequencing, single-bacterium transplantation, and exogenous supplementation demonstrated that WDD-regulated *Parasutterella* activated the FXR/PPARα/CYP4A12A axis through the putative *Parasutterella*-associated metabolite 7α-OH-T, thereby improving hepatic lipid metabolism in MAFLD mice. Despite the systematic design of this study, several limitations remain. First, only male C57BL/6 J mice were used; therefore, the generalizability of the findings across sexes remains uncertain. Sex differences may influence the development and progression of MAFLD and microbe-metabolite interactions through pathways involving sex hormones, immune responses, and gut microbiota composition. Future studies involving both sexes and different age groups may improve the robustness of the MAFLD model and better reflect real-world conditions. Second, microbial screening was conducted at the genus level. Although *P. excrementihominis* was selected as a representative species based on the literature and experimental evaluation, further species-level validation and refinement using metagenomic approaches are required. Third, although 7α-OH-T was detected in portal venous serum and its level depended on *P. excrementihominis* supplementation, the in vivo hydroxylation process leading to 7α-OH-T production remains unclear. Addressing this issue will require functional metagenomic screening and the establishment of a genetic manipulation system for *P. excrementihominis*.

Furthermore, clinically relevant factors, including viral infection and genetic background, should be considered in future in vivo and in vitro studies of MAFLD. The mechanisms underlying the multitarget effects of a single drug under multifactorial conditions also require further investigation. Intrahepatic cells are exposed to an environment in which biochemical and mechanical cues are closely integrated [24]. Recent studies have shown that macrophages, cholangiocytes [25], and hepatic stellate cells [26] interact with hepatocytes and contribute to the progression of MAFLD to MASH and ultimately HCC. Therefore, extending the focus from parenchymal cells to selected nonparenchymal cell populations is challenging but necessary.

## Conclusion

WDD remodeled the host microbiota, particularly by promoting the intestinal colonization of *Parasutterella*, which in turn regulated the closely associated metabolite 7α-OH-T. This metabolite was transported to the liver through the portal vein via the gut-liver axis, where it activated the FXR/PPARα/CYP4A12A axis, promoted FAO in hepatocytes, and alleviated MAFLD. This study identified a molecular basis for WDD-mediated cross-organ regulation through the microbiota-metabolite-host target axis and identified *P. excrementihominis* as a bacterium with BA- and fatty acid-modulating activity, together with 7α-OH-T as a lipid-lowering metabolite.

## Supplementary Information

Supplementary Material 1.

Supplementary Material 2.

## Data Availability

No datasets were generated or analysed during the current study.

## Abbreviations

ASVs: Amplicon sequence variants
Bas: Bile acids
CES2A: Carboxylesterase 2A
FAO: Fatty acid oxidation
HCC: Hepatocellular carcinoma
HDL: High density lipoprotein
HFD: High-fat diet
LDL: Low density lipoprotein
LEfSe: LDA effect size
MAFLD: Metabolic dysfunction-associated fatty liver disease
PCoA: Principal coordinate analysis
*P. excrementihominis*: *Parasutterella excrementihominis*
TCM: Traditional Chinese Medicine
TG: Triglyceride
WDD: Wendan Decoction
