# Supplementary material for: Wendan decoction modulates Parasutterella to influence fatty acid metabolism in MAFLD via the FXR/PPARα/CYP4A12A axis
Source: Chin Med. 2026 Jul 20;21:200. doi: 10.1186/s13020-026-01474-1 (PMC13383561; doi:10.1186/s13020-026-01474-1)
Supplement: Supplementary file 1 — Supplementary Material 1. [file 13020_2026_1474_MOESM1_ESM.docx]

**Supplementary data**

Table. S1 Formula compatibility of WDD

| No. | Chinese name | Latin name | Vegetal product | Family | Part used | Dose (g) |
| --- | --- | --- | --- | --- | --- | --- |
| 1 | Fabanxia | Pinelliae Rhizoma Praeparatum | *Pinellia ternate* (Thunb.) Breit. | Araceae | Rhizome | 9 |
| 2 | Zhuru | Bambusae Caulis in Taenias | *Phyllostachys nigra* (Lodd.) Munro var. *henonis* (Mitf.) Stapf ex Rendle | Poaceae | Caulis | 6 |
| 3 | Zhishi | Aurantii Fructus Immaturus | *Citrus aurantium* L. | Rutaceae | Fructus | 6 |
| 4 | Chenpi | Citri Reticulatae Pericarpium | *Citrus reticulata* Blanco | Rutaceae | Pericarpium | 6 |
| 5 | Gancao | Glycyrrhizae Radix et Rhizoma | *Glycyrrhiza uralensis* Fisch | Fabaceae | Radix | 3 |
| 6 | Shengjiang | Zingiberis Rhizoma Recens | *Zingiber officinale* Rosc. | Zingiberaceae | Fruit | 3 |

Table. S2 Non-absorbable components of WDD (detected in the contents of the cecum)

| No. | t_R_/min | Compound name | Molecular formula | M(Da) | Ion type | Error (ppm) | Characteristic Fragment | Source |
| --- | --- | --- | --- | --- | --- | --- | --- | --- |
| 1 | 36.21 | Oleic acid | C_18_H_34_O_2_ | 282.46 | [M+H]^+^ | -0.7 | 57.0312, 81.0775, 89.0596, 283.2641 | A |
| 2 | 32.02 | Linoleic acid | C_18_H_32_O_2_ | 280.45 | [M+H]^+^ | -0.8 | 57.0720, 69.0672, 141.1259, 149.0285 | A |
| 3 | 39.75 | Palmitic acid | C_16_H_32_O_2_ | 256.42 | [M-H]^-^ | 3.7 | 142.9913, 151.0158, 166.8678, 255.2331 | A |
| 4 | 15.41 | Azelaic acid | C_9_H_16_O_4_ | 188.22 | [M-H]^-^ | 0.4 | 43.0223, 57.0349, 123.0837, 187.0979 | A |
| 5 | 26.2 | HYDROXYCINNAMIC ACID | C_9_H_8_O_3_ | 164.16 | [M+H]^+^ | 1 | 79.0527, 165.0525, 165.1109, 165.1264 | A |
| 6 | 5.89 | Guanosine | C_10_H_13_N_5_O_5_ | 283.24 | [M+H]^+^ | -2.6 | 284.0814, 284.0932, 284.1145, 284.1464 | A, B |
| 7 | 1.8 | Uridine | C_9_H_12_N_2_O_6_ | 244.2 | [M-H]^-^ | 1.1 | 41.9986, 66.0345 | A |
| 8 | 1.09 | Adenine | C_5_H_5_N_5_ | 135.13 | [M+H]^+^ | 0.4 | 109.0107, 119.0412, 136.0412, 136.0601 | A |
| 9 | 37.62 | HEXADECANAMIDE | C_16_H_33_NO | 255.44 | [M+H]^+^ | -1.1 | 256.2633 | A |
| 10 | 0.59 | L(+)-Arginine | C_6_H_14_N_4_O_2_ | 174.2 | [M+H]^+^ | -1.1 | 60.0553, 70.0652, 158.0925, 175.1196 | A |
| 11 | 0.63 | Valine | C_5_H_11_NO_2_ | 117.15 | [M+H]^+^ | -1 | 31.0178, 43.0184, 55.0546, 58.0654 | A, B |
| 12 | 12.21 | LEUCINE | C_14_H_19_NO_4_ | 265.3 | [M+H]^+^ | 1.2 | 100.0741, 100.0789, 118.0837 | A |
| 13 | 0.61 | L-Glutamic acid | C_5_H_9_NO_4_ | 147.13 | [M+H]^+^ | -0.5 | 41.0390, 84.0446, 102.0655, 130.0491 | A, B |
| 14 | 1.63 | L-Isoleucine | C_6_H_13_NO_2_ | 131.17 | [M+H]^+^ | -0.9 | 30.0340, 41.0386, 57.0579, 86.0970 | A |
| 15 | 33.72 | glycine linoleamide | C_20_H_35_NO_3_ | 337.5 | [M+H]^+^ | -0.7 | 46.0655 | A |
| 16 | 2.6 | N-ISOVALERYLGLYCINE | C_7_H_13_NO_3_ | 159.18 | [M+H]^+^ | 1.8 | 41.0383, 46.0621, 56.9425, 69.0353 | A |
| 17 | 38.51 | MONOPALMITIN | C_19_H_38_O_4_ | 330.5 | [M+H]^+^ | -0.1 | 41.0371, 174.9921, 239.2363, 331.2097 | A |
| 18 | 37.48 | 1-MONOLINOLEIN | C_21_H_38_O_4_ | 354.52 | [M+H]^+^ | -2.8 | 73.0474, 245.2236, 337.2788, 355.2862 | A |
| 19 | 18.06 | LIQUIRITIGENIN | C_15_H_12_O_4_ | 256.25 | [M-H]^-^ | -3 | 77.0412, 91.0185, 104.9956, 131.0094 | A, F |
| 20 | 36.16 | 18β-Glycyrrhetinic Acid | C_30_H_46_O_4_ | 470.69 | [M+H]^+^ | -1.1 | 453.2868, 471.3452, 471.3680 | A, F |
| 21 | 40.12 | ORIENTIN | C_21_H_20_O_11_ | 448.38 | [M+H]^+^ | -0.5 | 73.0449, 342.9924, 343.0277, 449.0955 | B |
| 22 | 40.12 | HOMOORIENTIN | C_21_H_20_O_11_ | 448.38 | [M+H]^+^ | -0.5 | 73.0449, 342.9924, 343.0277, 449.0955 | B |
| 23 | 0.67 | p-Coumaric acid | C_9_H_8_O_3_ | 164.16 | [M+H]^+^ | -1.6 | 77.0379, 95.0496, 103.0575, 147.0455 | B |
| 24 | 0.65 | Sucrose | C12H22O11 | 342.3 | [M-H]^-^ | 0.6 | 43.0192, 59.0136, 75.0081, 131.0350 | B |
| 25 | 17.14 | Hesperetin | C16H14O6 | 302.28 | [M-H]^-^ | -1.5 | 41.0028, 57.0342, 91.0556, 109.0293 | C |
| 26 | 25.16 | Diosmin | C28H32O15 | 608.54 | [M+H]^+^ | -3.4 | 133.0864, 371.2286, 503.3057, 547.3319 | C, D |
| 27 | 10.5 | 6-Demethoxylnobiletin | C_20_H_20_O_7_ | 372.37 | [M-H]^-^ | 7.7 | 371.1151 | C, D |
| 28 | 39.52 | PONCIRIN | C_28_H_34_O_14_ | 594.57 | [M+H]^+^ | 0.9 | 59.0605, 594.9043, 595.4217 | C, D |
| 29 | 41.27 | Rutin | C_27_H_30_O_16_ | 610.52 | [M+H]^+^ | 10.8 | 73.0467, 223.0638, 341.0177, 445.1206 | C, F |
| 30 | 39.52 | DIDYMIN | C_28_H_34_O_14_ | 594.56 | [M+H]^+^ | 0.9 | 59.0605, 594.9043, 595.4217 | C |
| 31 | 10.8 | Prunin | C_21_H_22_O_10_ | 434.4 | [M-H]^-^ | 0 | 313.0721, 315.0824 | C |
| 32 | 18.78 | 5,7,4'-Trimethylapigenin | C_18_H_16_O_5_ | 312.32 | [M+H]^+^ | -0.8 | 149.0204, 253.0866 | C |
| 33 | 10.5 | QUERCETIN-3,5,7,3',4'-PENTAMETHYL ETHER | C_20_H_20_O_7_ | 372.37 | [M-H]^-^ | 7.7 | 371.1151 | C |
| 34 | 12.22 | Thymol | C_10_H_14_O | 150.22 | [M+H]^+^ | -1 | 45.0338, 91.0500, 109.0645, 133.1016 | C |
| 35 | 14.51 | MERANZIN | C_15_H_16_O_4_ | 260.29 | [M+H]^+^ | -0.7 | 215.1054, 261.1095, 261.1263 | C |
| 36 | 17.51 | 7-Hydroxycoumarin | C_9_H_6_O_3_ | 162.14 | [M+H]^+^ | -0.9 | 41.0369, 43.0177, 55.0550, 95.0337 | C |
| 37 | 21.59 | Osthole | C_15_H_16_O_3_ | 244.29 | [M+H]^+^ | 1.1 | 43.0545, 157.0267, 175.0348, 245.1156 | C |
| 38 | 10.42 | Tangeretin | C_20_H_20_O_7_ | 372.37 | [M-H]^-^ | 7.7 | 371.1151 | D |
| 39 | 15.02 | SINENSETIN | C_20_H_20_O_7_ | 372.37 | [M-H]^-^ | 5.2 | 178.9915, 307.1523, 371.1154, 371.1988 | D |
| 40 | 10.5 | Isohemiphloin | C_21_H_22_O_10_ | 434.39338 | [M-H]^-^ | 0 | 313.0721, 315.0824 | D, E |
| 41 | 18.01 | 2-(4-Methoxyphenyl)-3,5,7-trimethoxy-4H-1-benzopyran-4-one | C_19_H_18_O_6_ | 342.34 | [M-H]^-^ | 5.4 | 57.0337, 80.9657, 83.0507, 180.9885 | D |
| 42 | 13.32 | CARVEOL | C_10_H_16_O | 152.23 | [M-H]^-^ | -1.1 | 93.0435, 135.0814, 135.0966, 151.1172 | D |
| 43 | 12.35 | O-CYMENE | C_10_H_14_ | 134.22 | [M+H]^+^ | -0.7 | 41.0388, 67.0535, 79.0562, 107.0856 | D |
| 45 | 3.81 | Methyl 2-(methylamino)benzoate | C_9_H_11_NO_2_ | 165.19 | [M+H]^+^ | -0.7 | 77.0390, 103.0548, 120.0823, 166.0858 | D |
| 46 | 22.78 | Vanillylacetone | C_11_H_14_O_3_ | 194.23 | [M-H]^-^ | 1 | 177.0556, 193.0876 | E |
| 47 | 27.5 | 6-Gingerol | C_17_H_26_O_4_ | 294.39 | [M+H]^+^ | -2.3 | 83.0837, 137.0571, 175.1118, 193.1235 | E |
| 48 | 32.78 | 8-Gingerol | C_19_H_30_O_4_ | 322.4 | [M+H]^+^ | -4.9 | 149.0256, 323.2467 | E |
| 49 | 20.92 | 10-Gingerol | C_21_H_34_O_4_ | 350.49 | [M+H]^+^ | -0.9 | 93.0668, 105.0712, 133.0995, 159.1131 | E |
| 50 | 31.01 | 6-Shogaol | C_17_H_24_O_3_ | 276.37 | [M+H]^+^ | -1.9 | 43.0191, 203.0878, 235.1663, 277.1847 | E |
| 51 | 18.55 | 8-SHOGAOL | C_19_H_28_O_3_ | 304.42 | [M+H]^+^ | -2.9 | 69.0731, 111.1133, 132.9061, 305.2042 | E |
| 52 | 25.54 | Gingerenone A | C_21_H_24_O_5_ | 356.41 | [M+H]^+^ | -2.2 | 357.1680 | E |
| 53 | 19.14 | 10-Shogaol | C_21_H_32_O_3_ | 332.48 | [M+H]^+^ | 0.9 | 183.0998, 209.1196, 333.2439 | E |
| 54 | 18.41 | Diacetoxy-6-gingerdiol | C_21_H_32_O_6_ | 380.48 | [M+H]^+^ | -2 | 349.1698, 349.1886, 363.2147, 381.2376 | E |
| 55 | 34.89 | Methyl diacetoxy-6-gingerdiol | C_22_H_34_O_6_ | 394.5 | [M+H]^+^ | -9.3 | 395.2053, 395.2448, 395.3052 | E |
| 56 | 19.98 | Dihydrocapsaicin | C_18_H_29_NO_3_ | 307.43 | [M+H]^+^ | -3.8 | 46.0651, 101.0585, 290.2056, 308.2199 | E |
| 57 | 14.99 | DEMETHOXYCURCUMIN | C_20_H_18_O_5_ | 338.35 | [M+H]^+^ | -8 | 339.1204, 339.1442 | E |
| 58 | 18.01 | Isoliquiritigenin | C_15_H_12_O_4_ | 256.25 | [M-H]^-^ | -3 | 77.0412, 104.9956, 121.0322, 131.0094 | F |
| 60 | 12.47 | APIGENIN-6-GLUCOSIDE-8-ARABINOSIDE | C_26_H_28_O_14_ | 564.5 | [M-H]^-^ | -0.5 | 335.0529, 383.0789, 443.0982, 563.1820 | F |
| 61 | 11.8 | APIGENIN-6-ARABINOSIDE-8-GLUCOSIDE | C_26_H_28_O_14_ | 564.49 | [M-H]^-^ | -0.9 | 297.0803, 353.0655, 353.0784, 443.0971 | F |
| 62 | 18.54 | Formononetin | C_16_H_12_O_4_ | 268.26 | [M-H]^-^ | 1 | 104.9946, 156.9883, 176.9976, 182.9896 | F |
| 63 | 18.02 | CALYCOSIN | C_16_H_12_O_5_ | 284.26 | [M+H]^+^ | -2.8 | 229.0848, 285.0739 | F |
| 64 | 11.41 | LICOISOFLAVONE A | C_20_H_18_O_6_ | 354.35 | [M+H]^+^ | -8.7 | 355.1175, 355.2217 | F |
| 65 | 18.78 | 5,7-Dihydroxy-3-(5-hydroxy-2,2-dimethyl-2H-1-benzopyran-6-yl)-4H-1-benzopyran-4-one | C_20_H_16_O_6_ | 352.34 | [M+H]^+^ | -7 | 353.1017, 353.1866, 353.2028, 353.2457 | F |
| 66 | 11.41 | Glyasperin F | C_20_H_18_O_6_ | 354.35 | [M+H]^+^ | -8.7 | 355.1175, 355.2217 | F |
| 67 | 18.78 | SeMilicoisoflavone B | C_20_H_16_O_6_ | 352.34 | [M+H]^+^ | -7 | 353.1017, 353.1866, 353.2028, 353.2457 | F |
| 68 | 14.99 | Licoflavone C | C_20_H_18_O_5_ | 338.35 | [M+H]^+^ | -8 | 339.1204, 339.1442 | F |
| 69 | 14.99 | 8-PRENYLGENISTEIN | C_20_H_18_O_5_ | 338.35 | [M+H]^+^ | -8 | 339.1204, 339.1442 | F |
| 70 | 26.03 | Oleanolic acid | C_30_H_48_O_3_ | 456.7 | [M+H]^+^ | -2.2 | 439.2611, 457.2569, 457.3659 | F |
| 71 | 33.65 | Cinnamic acid | C_9_H_8_O_2_ | 148.16 | [M+H]^+^ | -0.2 | 77.0401, 121.0288, 149.0240, 149.0376 | F |

**Note:** A: *Pinellia ternate* (Thunb.) Breit.; B: *Phyllostachys nigra* (Lodd.) Munro var. *henonis* (Mitf.) Stapf ex Rendle; C: *Citrus aurantium* L.; D: *Citrus reticulata* Blanco; E: *Zingiber officinale* Rosc.; F: *Glycyrrhiza uralensis* Fisch.

Table. S3 KEGG entries in the non-targeted metabolome of cecal contents

| **KEGG term** | ***P* value** | **Enrichment_score** | **Groups** |
| --- | --- | --- | --- |
| Nicotinate and nicotinamide metabolism | 0.00639905200222313 | 3.52903795233892 | Mod vs. Con |
| Ascorbate and aldarate metabolism | 0.00762152061549297 | 3.4052120592744 |  |
| Tryptophan metabolism | 0.0132908956375209 | 2.72827231255118 |  |
| Nicotine addiction | 0.0179569183718506 | 9.24271844660194 |  |
| Pentose and glucuronate interconversions | 0.034227166164138 | 2.74148428500905 |  |
| Parkinson disease | 0.0445503385175246 | 3.73263629574309 |  |
| Autophagy - other | 0.0899260620294758 | 10.7831715210356 |  |
| Phenylalanine, tyrosine and tryptophan biosynthesis | 0.0920839785446172 | 2.77281553398058 |  |
| Efferocytosis | 0.136000240164127 | 3.08090614886731 |  |
| Galactose metabolism | 0.168532582914552 | 2.10975094976783 |  |
| Primary bile acid biosynthesis | 0.176229309845757 | 2.06486263168767 |  |
| Ubiquinone and other terpenoid-quinone biosynthesis | 0.194095068460119 | 1.74862240881658 |  |
| Steroid hormone biosynthesis | 0.200734002190548 | 1.60146111698548 |  |
| Tyrosine metabolism | 0.220067539 | 1.65894946477471 |  |
| Aldosterone-regulated sodium reabsorption | 0.22234346463832 | 4.04368932038835 |  |
| Cocaine addiction | 0.22234346463832 | 4.04368932038835 |  |
| Drug metabolism - cytochrome P450 | 0.281435979792164 | 1.48733400290146 |  |
| Insulin secretion | 0.314382997594035 | 2.6957928802589 |  |
| Cholinergic synapse | 0.314382997594035 | 2.6957928802589 |  |
| HIF-1 signaling pathway | 0.376253761795196 | 2.15663430420712 |  |
| Sphingolipid signaling pathway | 0.376253761795196 | 2.15663430420712 |  |
| Salivary secretion | 0.414395313027795 | 1.90291262135922 |  |
| Retrograde endocannabinoid signaling | 0.450225807477468 | 1.7026060296372 |  |
| Fatty acid degradation | 0.461450615650053 | 1.29398058252427 |  |
| Aminoacyl-tRNA biosynthesis | 0.482028324483291 | 1.24421209858103 |  |
| Fatty acid biosynthesis | 0.521719451353244 | 1.15533980582524 |  |
| cAMP signaling pathway | 0.545197583346967 | 1.29398058252427 |  |
| Purine metabolism | 0.611063073661323 | 0.960876670191291 |  |
| D-Amino acid metabolism | 0.636235649921528 | 0.937667088785704 |  |
| Biosynthesis of unsaturated fatty acids | 0.636235649921528 | 0.937667088785704 |  |
| Sphingolipid metabolism | 0.66870449882001 | 0.924271844660194 |  |
| Vitamin digestion and absorption | 0.689083894887063 | 0.874311204408292 |  |
| Tyrosine metabolism | 9.25559448989307e-07 | 5.57190635451505 | WDD vs. Mod |
| Tryptophan metabolism | 7.26720648615335e-05 | 4.36354112100576 |  |
| Phenylalanine, tyrosine and tryptophan biosynthesis | 0.00242550540979875 | 5.17391304347826 |  |
| Nicotinate and nicotinamide metabolism | 0.00366778436650757 | 3.95098814229249 |  |
| Ascorbate and aldarate metabolism | 0.00439266961442414 | 3.81235697940503 |  |
| Dopaminergic synapse | 0.0415796765021344 | 6.03623188405797 |  |
| Arginine and proline metabolism | 0.0448117183024487 | 2.55052051439069 |  |
| Parkinson disease | 0.160190198203908 | 2.78595317725753 |  |
| Nicotine addiction | 0.17813157066251 | 5.17391304347826 |  |
| Lysine degradation | 0.200131851533613 | 1.94021739130435 |  |
| ABC transporters | 0.254231758066423 | 1.55773726040206 |  |
| Prolactin signaling pathway | 0.265424903222614 | 3.29249011857708 |  |
| HIF-1 signaling pathway | 0.343536616046949 | 2.41449275362319 |  |
| Serotonergic synapse | 0.379452163461627 | 2.1304347826087 |  |
| Fatty acid degradation | 0.404217892475156 | 1.44869565217391 |  |
| Thermogenesis | 0.475941529431003 | 1.57466918714556 |  |
| Arginine biosynthesis | 0.490511906974777 | 1.50905797101449 |  |
| Steroid hormone biosynthesis | 0.533339156427976 | 1.07576409814895 |  |
| Vitamin B6 metabolism | 0.557565338691226 | 1.24887556221889 |  |
| Pantothenate and CoA biosynthesis | 0.569888677728588 | 1.20724637681159 |  |
| Central carbon metabolism in cancer | 0.569888677728588 | 1.20724637681159 |  |
| Pentose phosphate pathway | 0.647144124600917 | 0.978848413631022 |  |
| alpha-Linolenic acid metabolism | 0.710646737808787 | 0.823122529644269 |  |
| Primary bile acid biosynthesis | 0.734269223932184 | 0.770582793709528 |  |
| Inositol phosphate metabolism | 0.734269223932184 | 0.770582793709528 |  |
| Glycine, serine and threonine metabolism | 0.741711303505469 | 0.754528985507246 |  |
| Aminoacyl-tRNA biosynthesis | 0.76947318425825 | 0.696488294314381 |  |
| Pyrimidine metabolism | 0.845333829244792 | 0.548748353096179 |  |
| Purine metabolism | 0.94340716376112 | 0.358588032716315 |  |
| Biosynthesis of various nucleotide sugars | 0.970945710025315 | 0.292075736325386 |  |
| Porphyrin metabolism | 0.983792744592585 | 0.251509661835749 |  |

Note: The blue font represented significantly enriched pathways; the red font represented pathways strongly correlated with MAFLD.

**Displays of Figures**

**Figure S1**

**
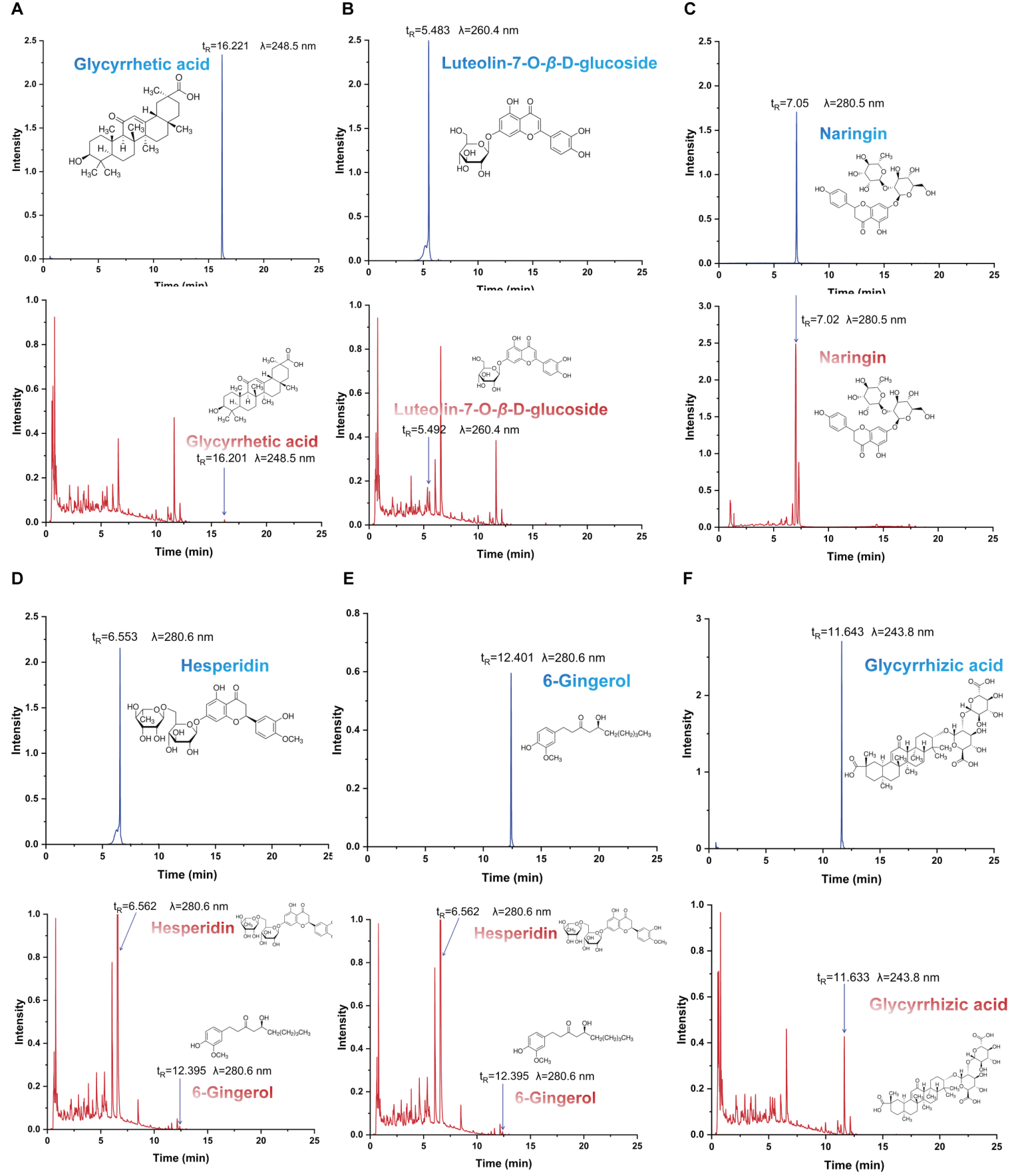
**

**Figure. S1 UPLC chromatogram of the representative components in WDD.** The blue curve denoted the reference standard, and the red curve represented WDD at a specific UV wavelength.

**
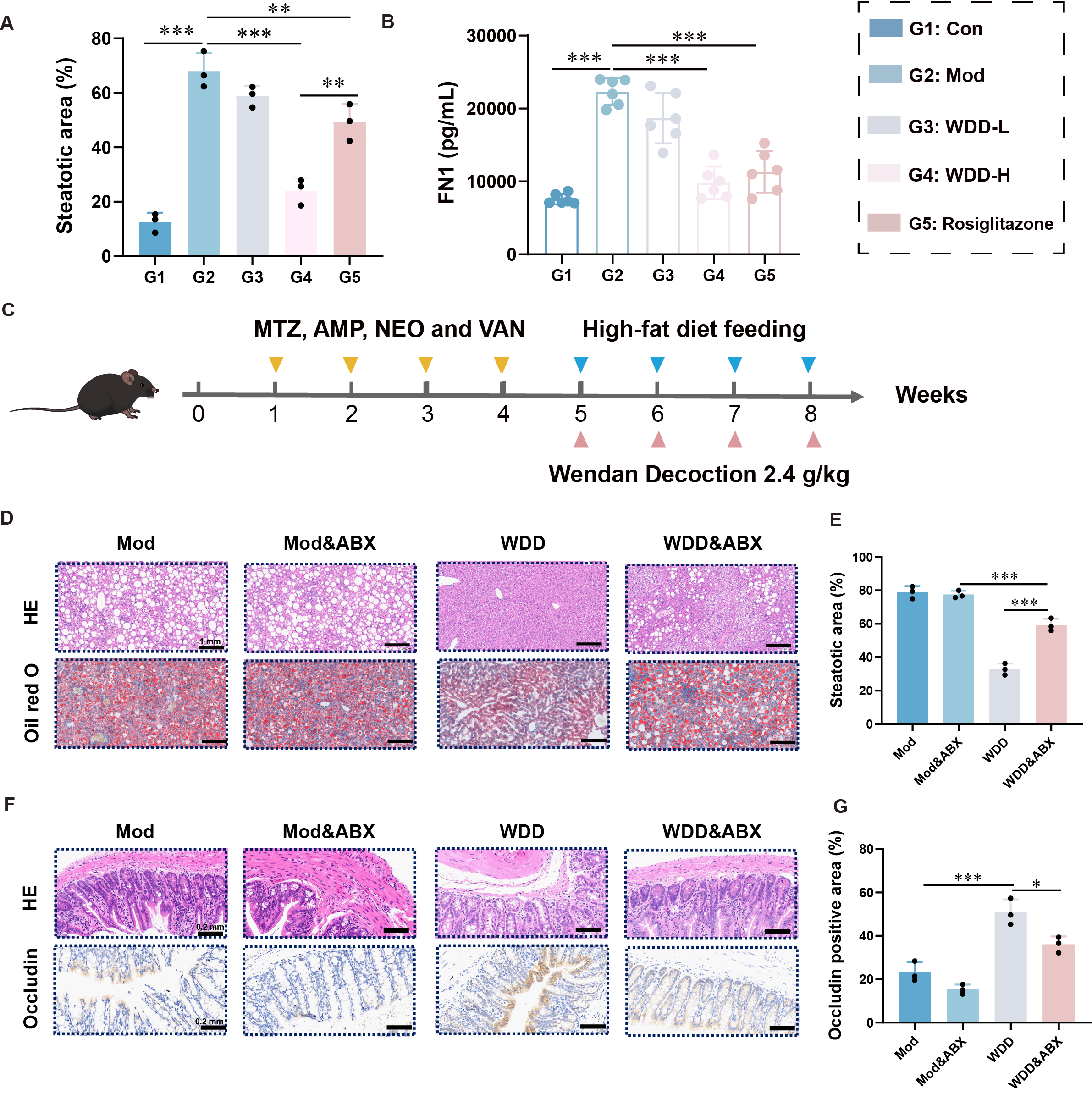
**

**Figure S2**

**Figure. S2 The impact of host microorganisms on the efficacy of WDD.** (A) Semi quantitative analysis of Oil Red O staining. (B) ELISA detection of FN1 in liver tissue. (C) Flowchart for the treatment of MAFLD mice with WDD. (D-E) Representative images of MAFLD mouse liver stained with HE and Oil Red O (scale bar=1 mm). (F-G) HE staining and Occludin immunohistochemistry of colon tissue (scale bar=0.2 mm). Data were presented as mean ± SD. *^*^P < 0.05, ^**^P < 0.01, ^***^P < 0.001* represented significance.

**Figure S3**

**
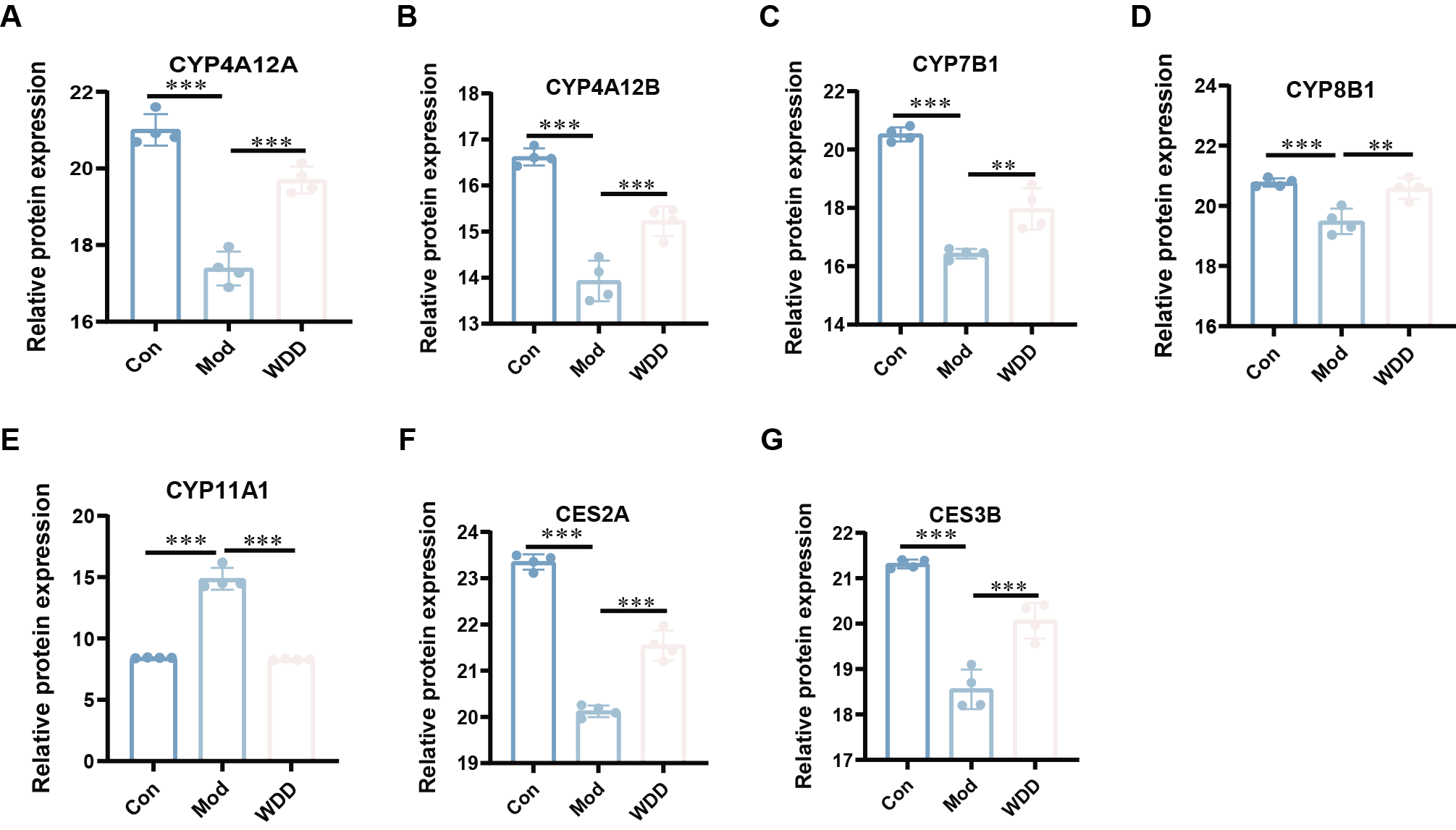
Figure. S3 WDD regulated multiple metabolic processes.** (A-G) Semi quantitative analysis of multiple key targets in proteomics. Data were presented as mean ± SD. *^**^P < 0.01, ^***^P < 0.001* represented significance.

**
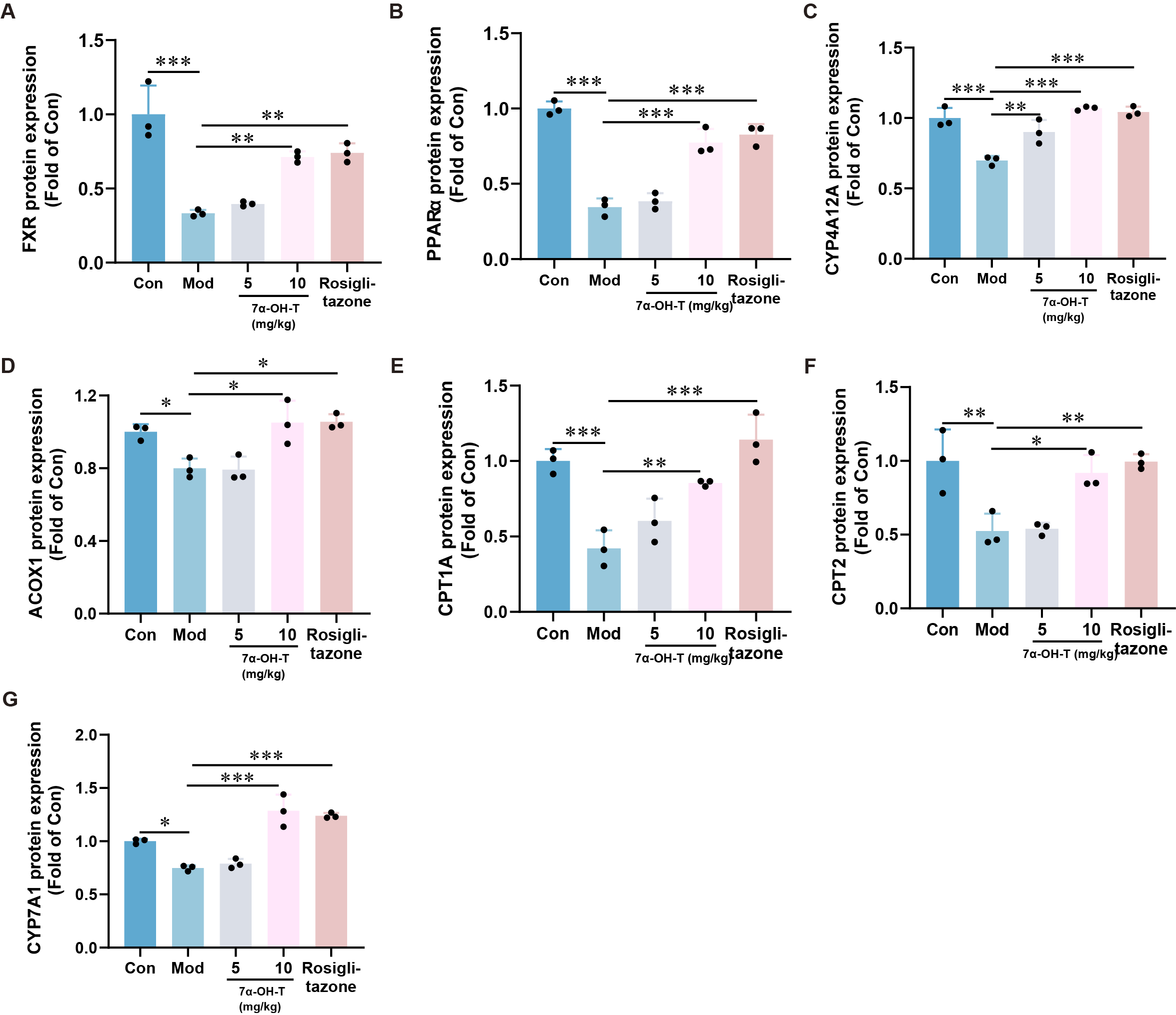
Figure S4**

**Figure. S4 7α-OH-T regulated lipid metabolism-related pathways.** (A-G) Quantitative analysis of FXR, PPARα, CYP4A12A, ACOX1, CPT1A, CPT2, CYP7A1 proteins. Data were presented as mean ± SD. *^*^P < 0.05, ^**^P < 0.01, ^***^P < 0.001* represented significance.
