# Supplementary material for: Wendan decoction modulates Parasutterella to influence fatty acid metabolism in MAFLD via the FXR/PPARα/CYP4A12A axis
Source: Chin Med. 2026 Jul 20;21:200. doi: 10.1186/s13020-026-01474-1 (PMC13383561; doi:10.1186/s13020-026-01474-1)
Supplement: Supplementary file 2 — Supplementary Material 2. [file 13020_2026_1474_MOESM2_ESM.pptx]

## Slide 1
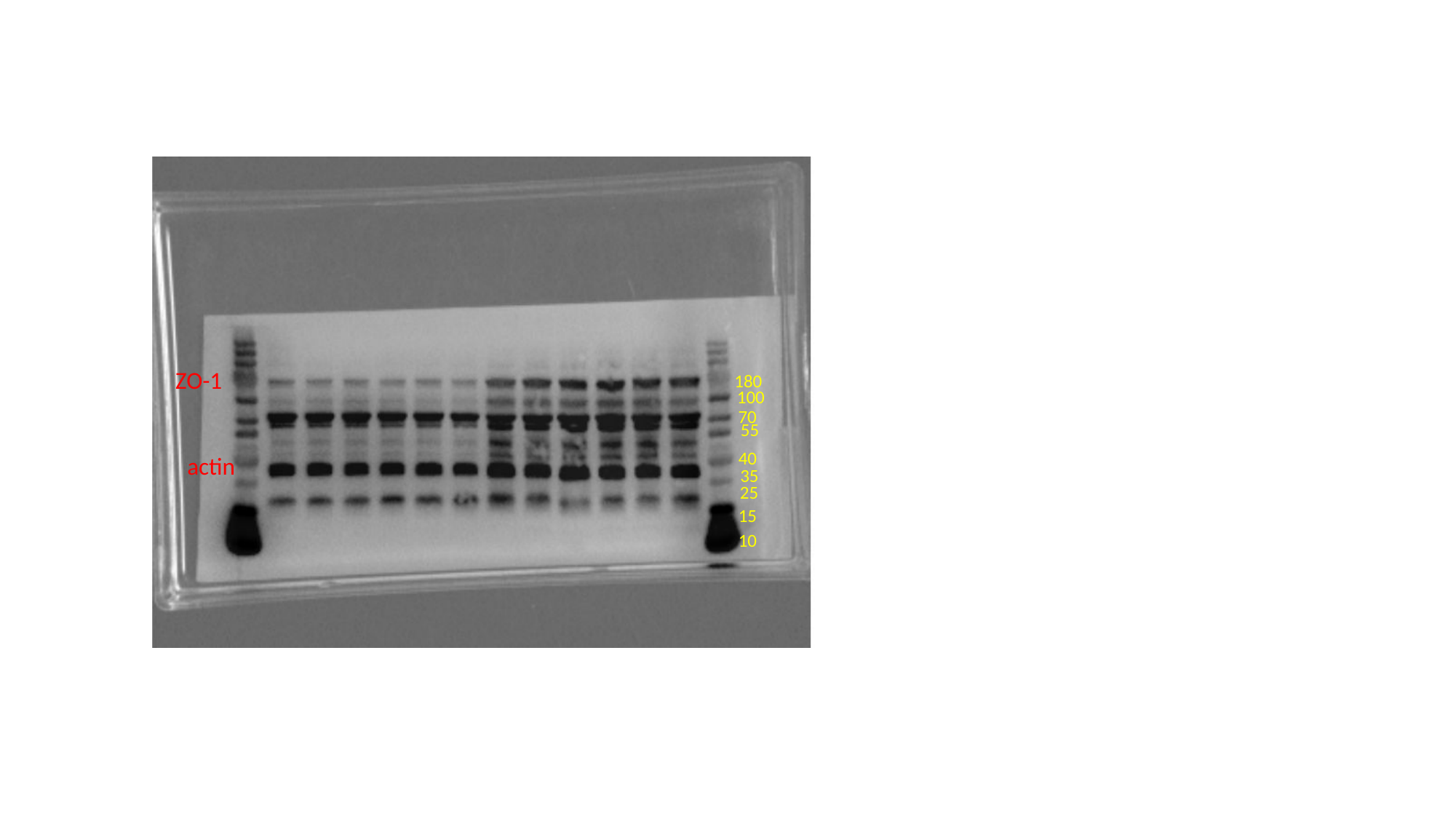

ZO-1
180
100
70
55
40
actin
35
25
15
10

## Slide 2
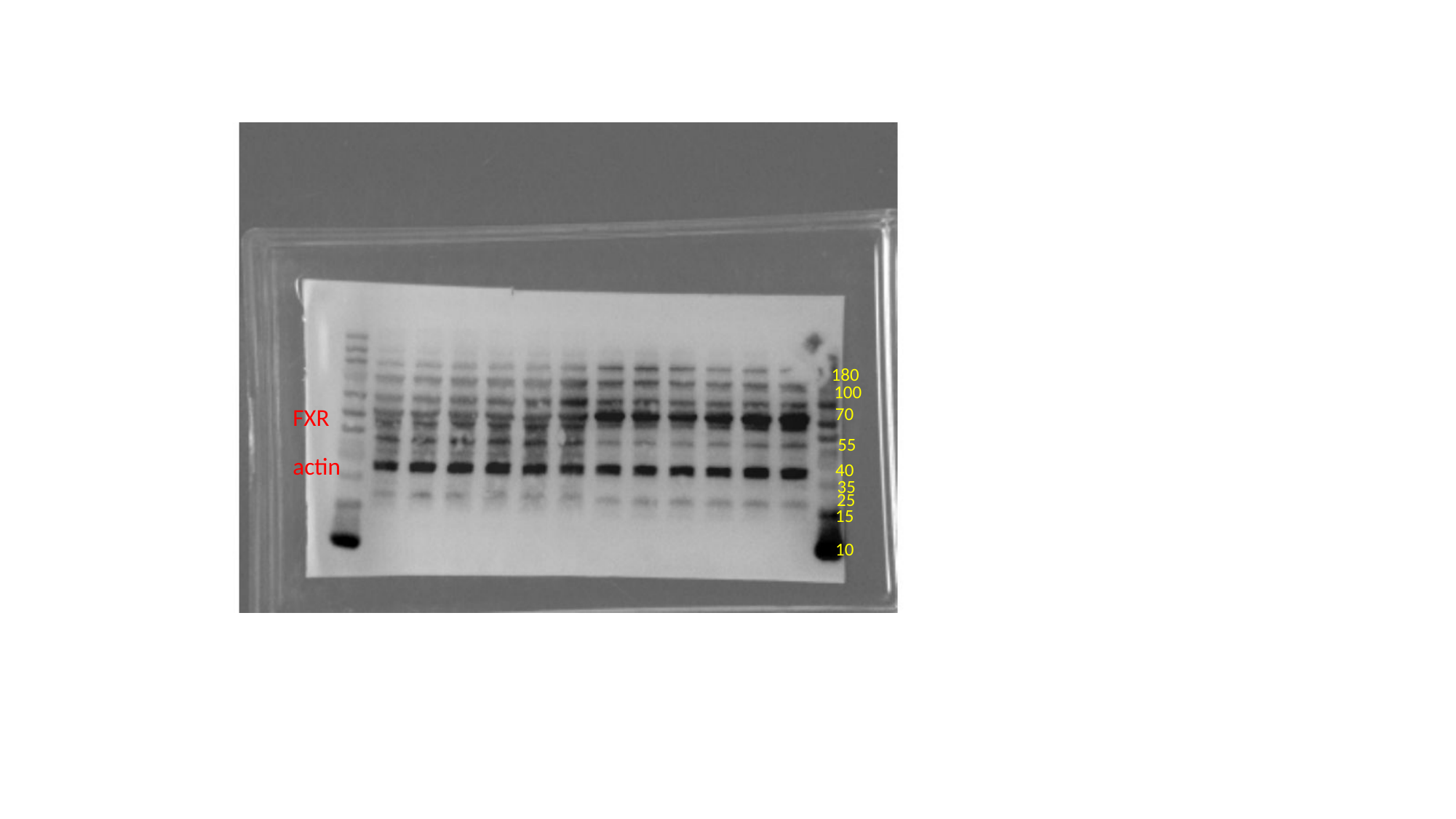

180
100
70
FXR
55
actin
40
35
25
15
10

## Slide 3
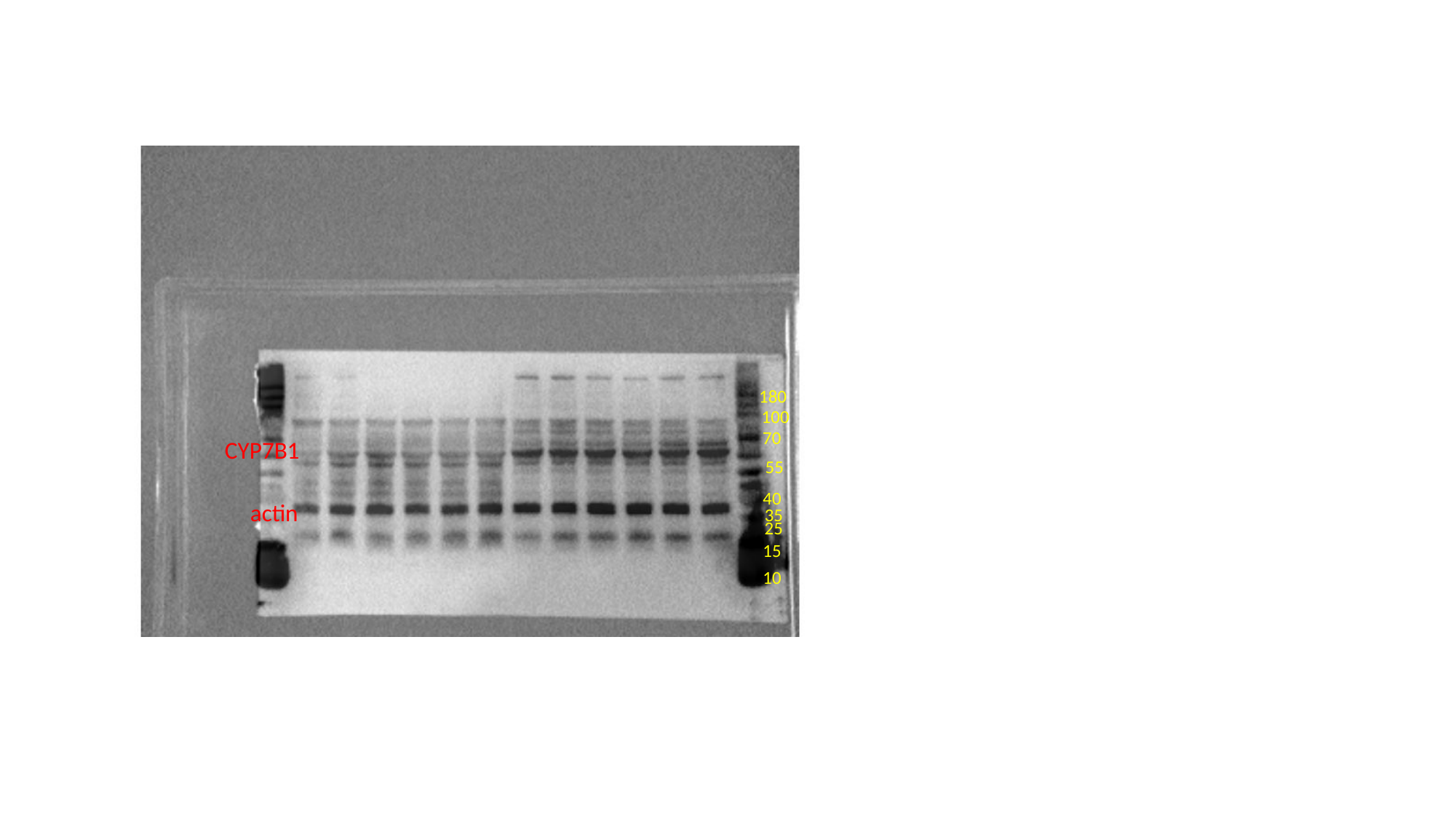

180
100
70
CYP7B1
55
40
actin
35
25
15
10

## Slide 4
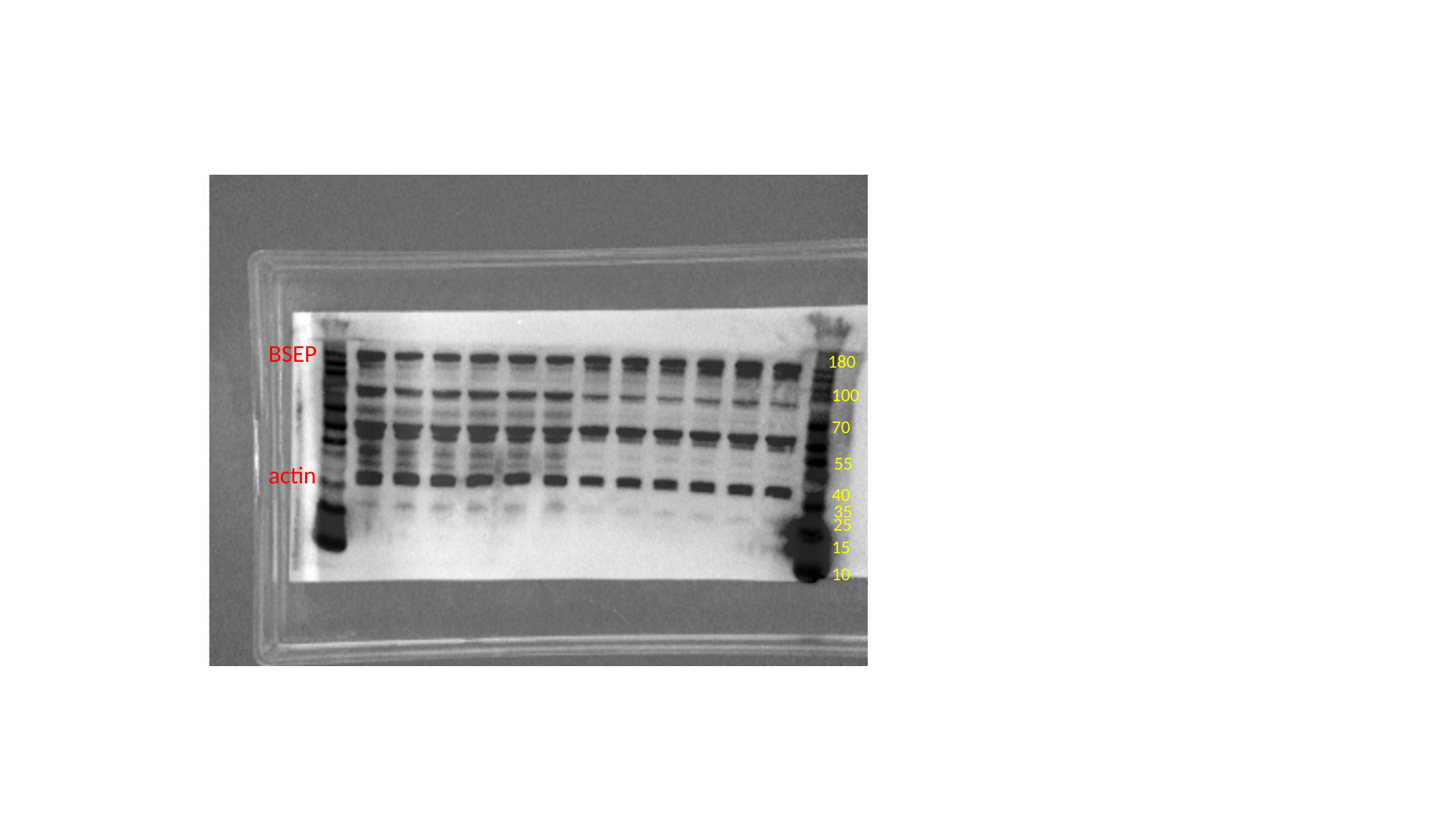

BSEP
180
100
70
55
actin
40
35
25
15
10

## Slide 5
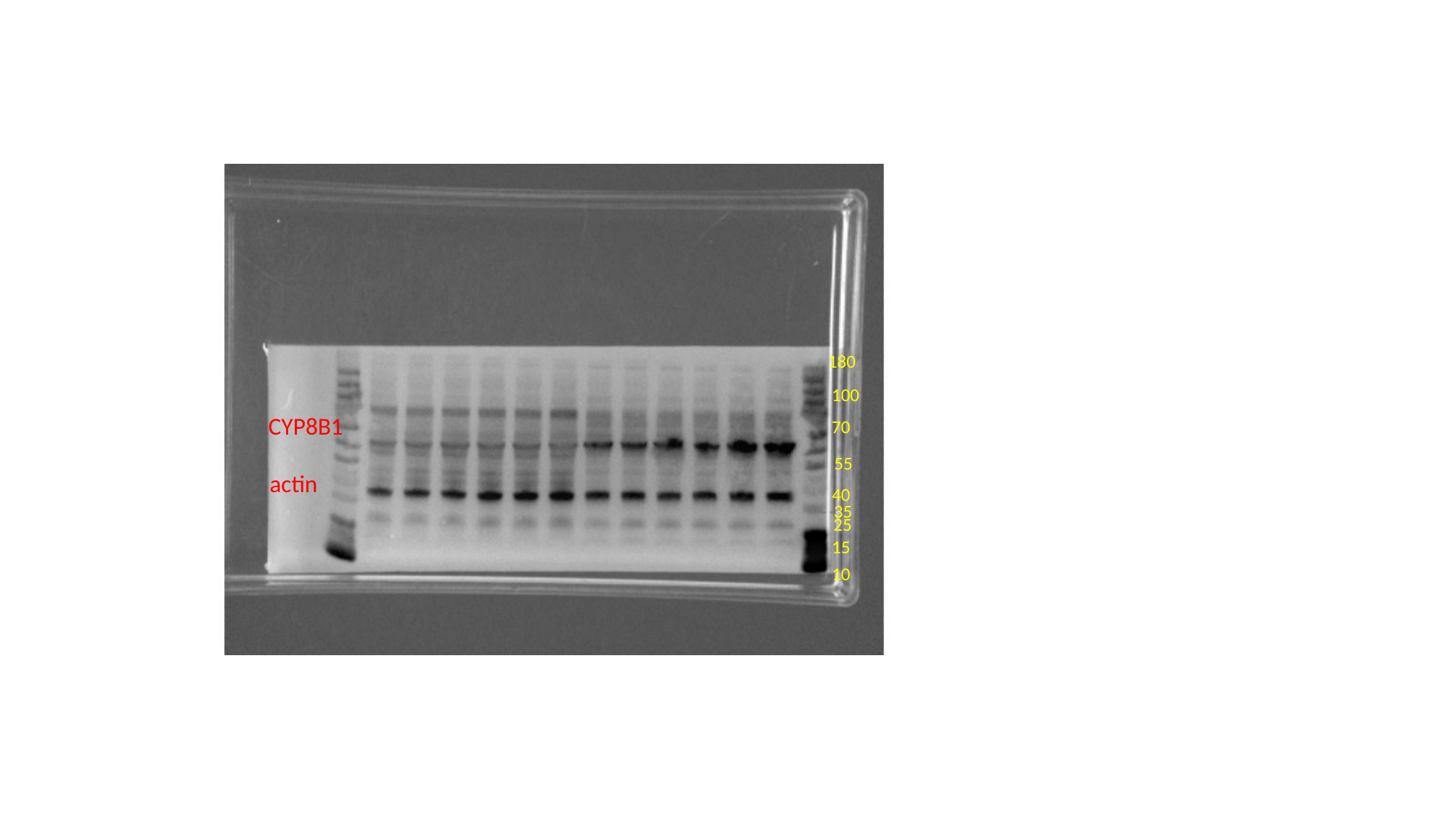

180
100
CYP8B1
70
55
actin
40
35
25
15
10

## Slide 6
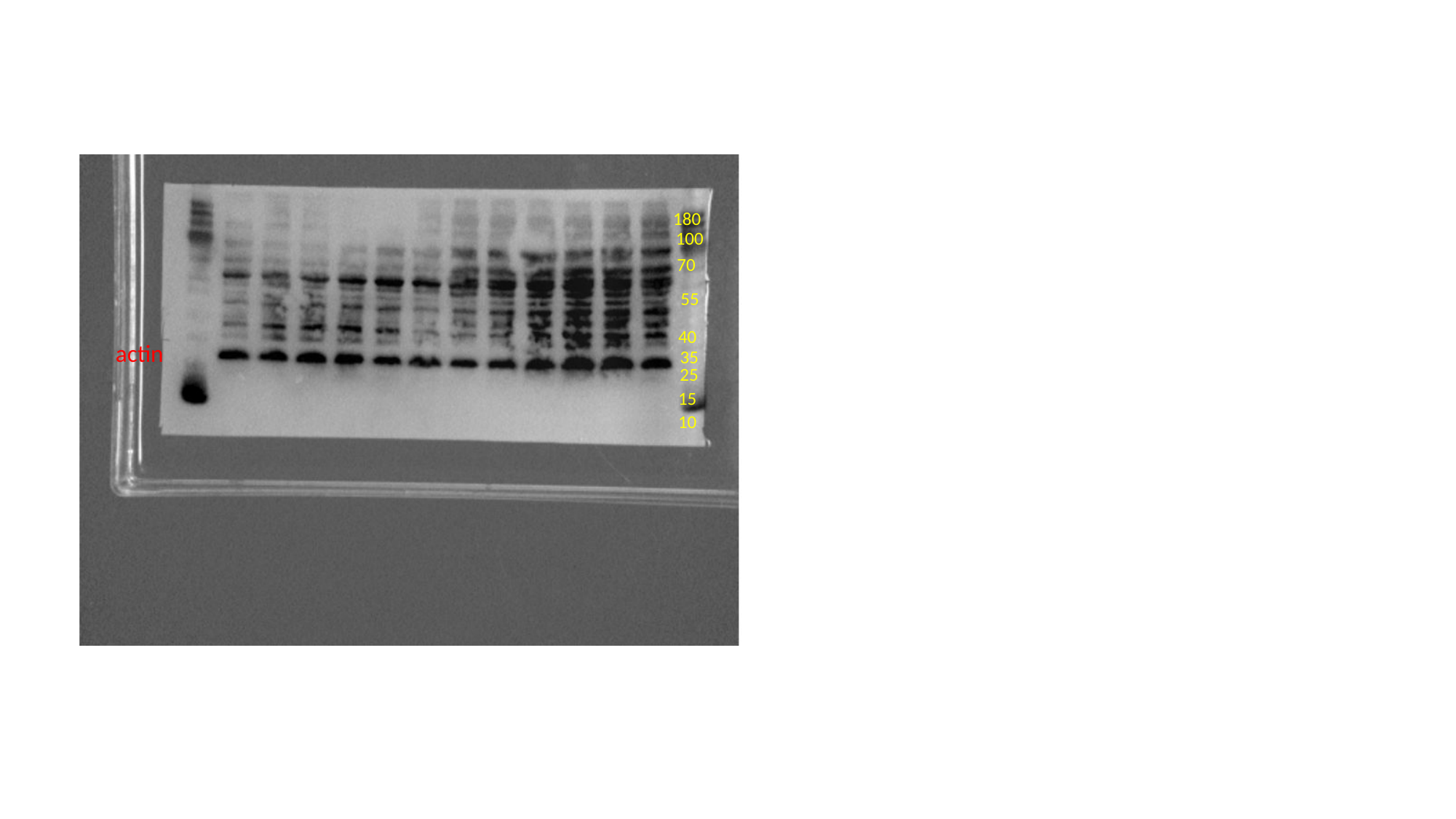

180
100
70
55
40
actin
35
25
15
10

## Slide 7
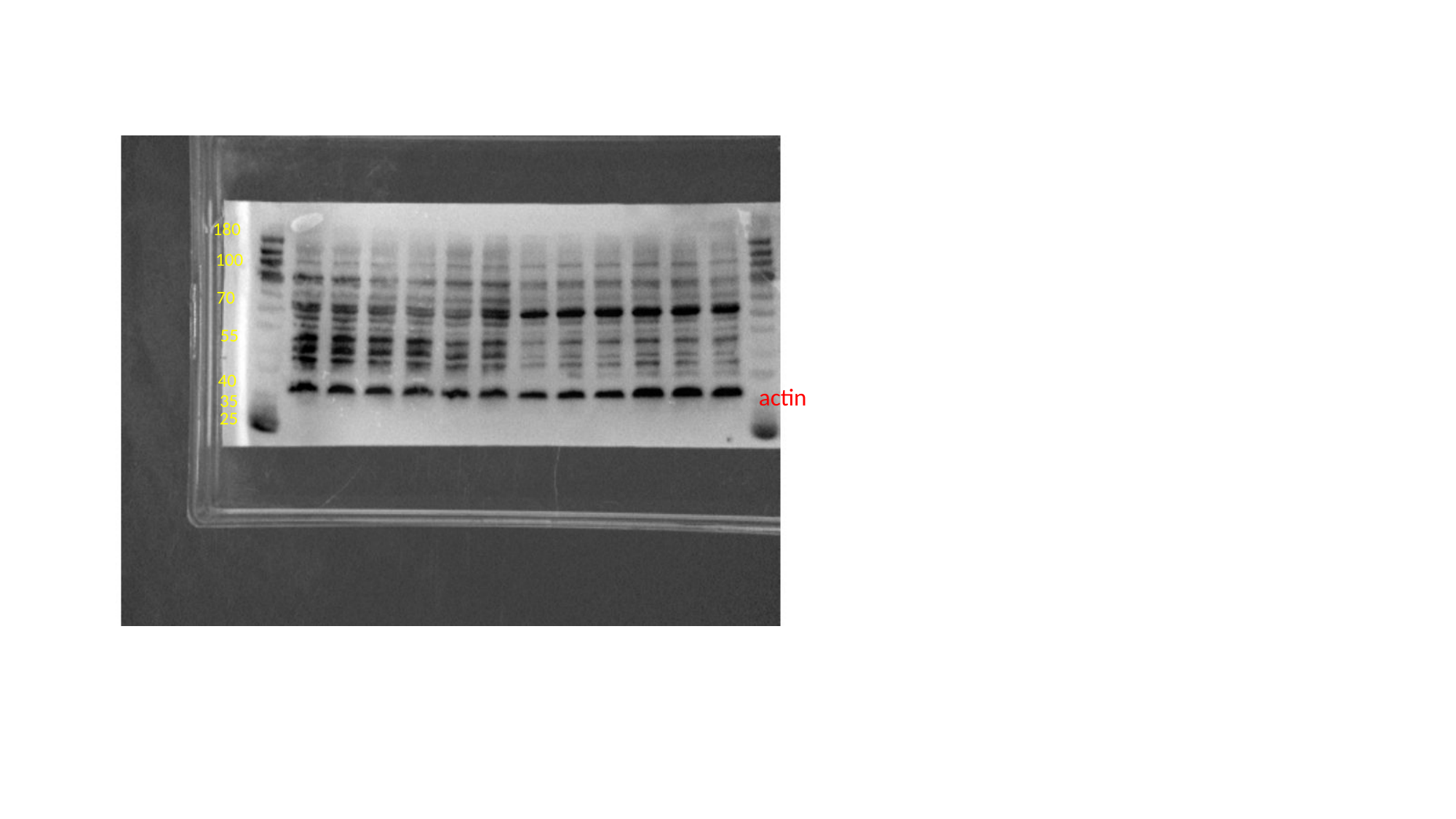

180
100
70
55
40
actin
35
25

## Slide 8
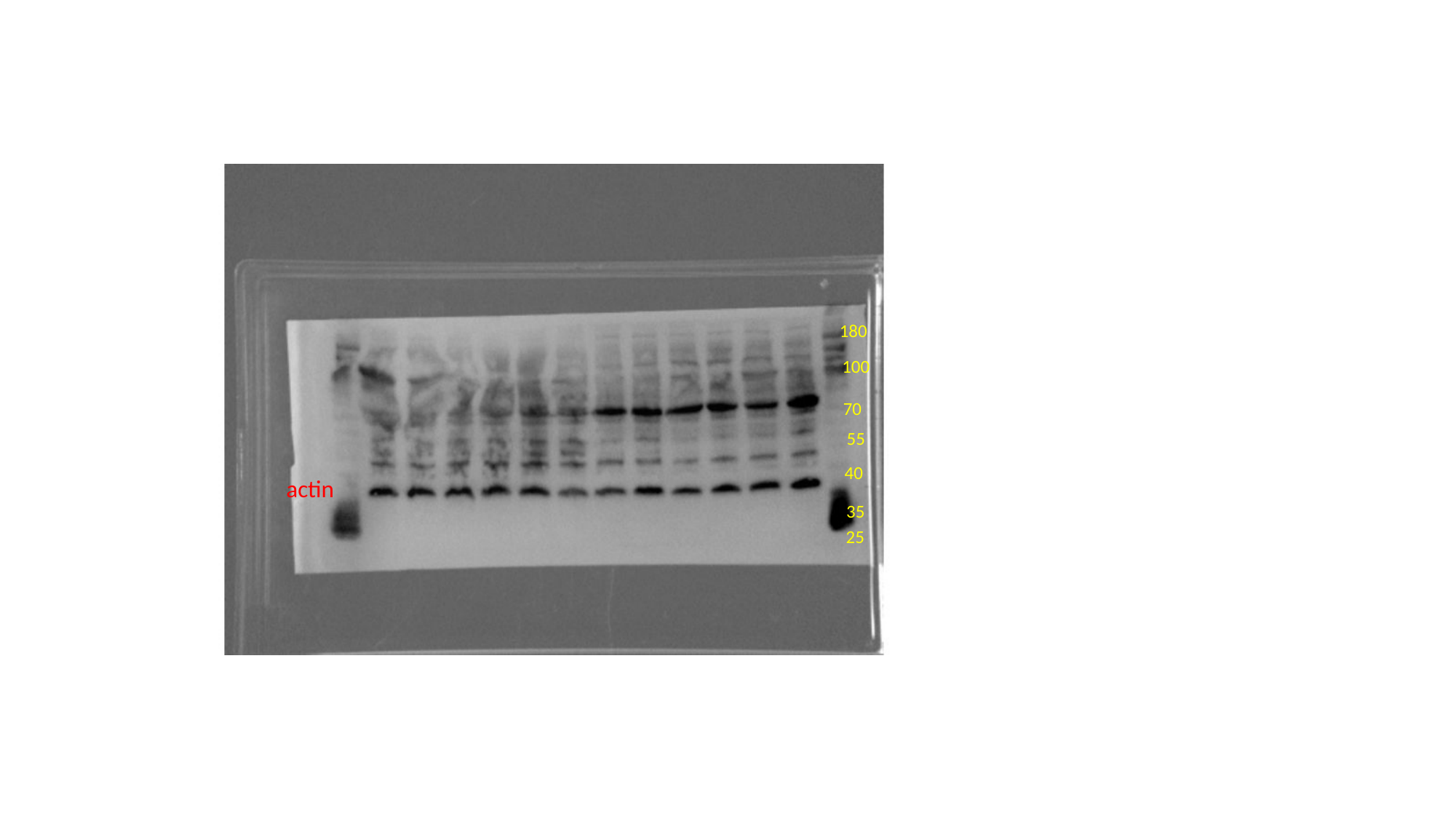

180
100
70
55
40
actin
35
25

## Slide 9
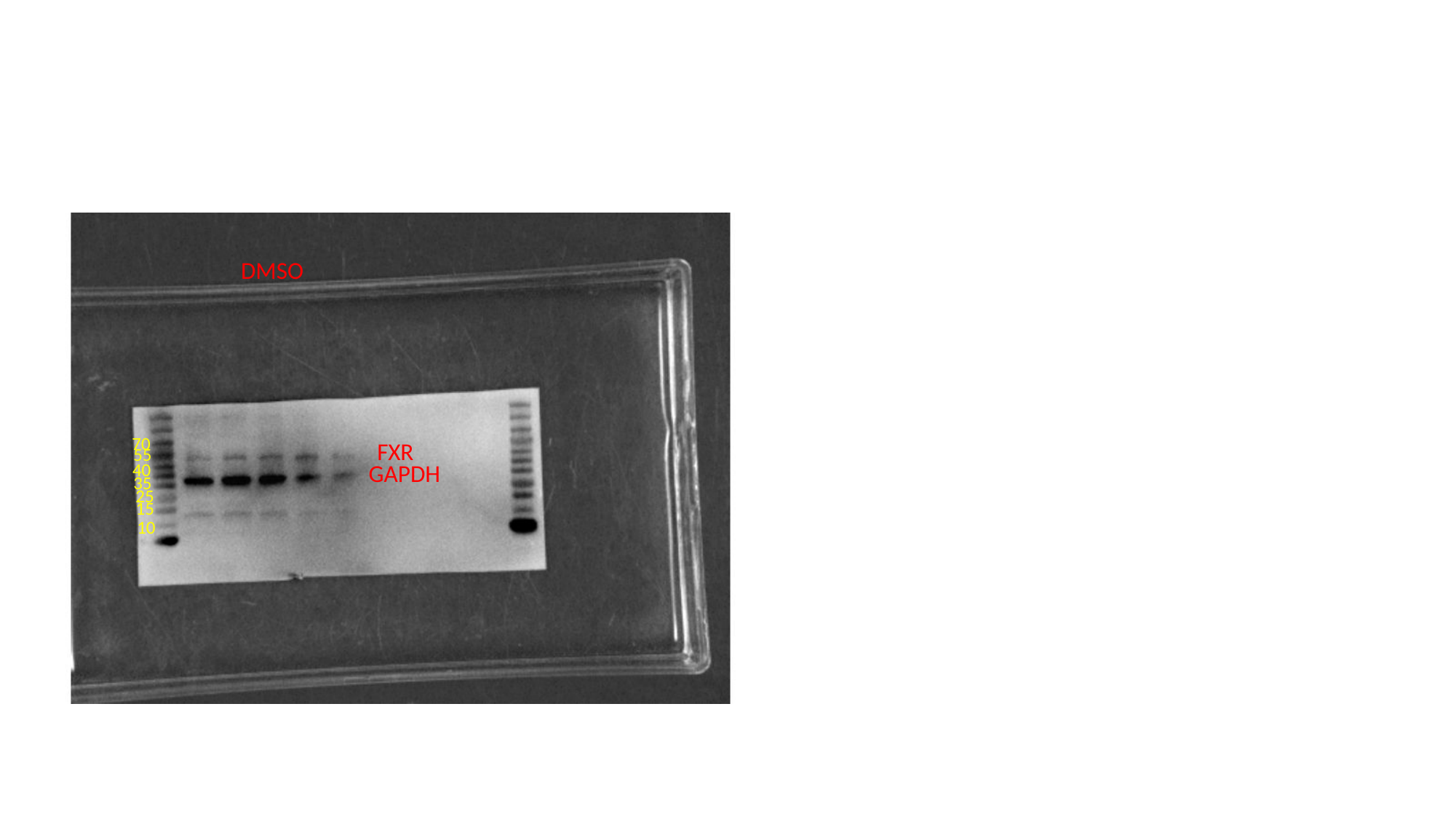

DMSO
70
FXR
55
40
GAPDH
35
25
15
10

## Slide 10
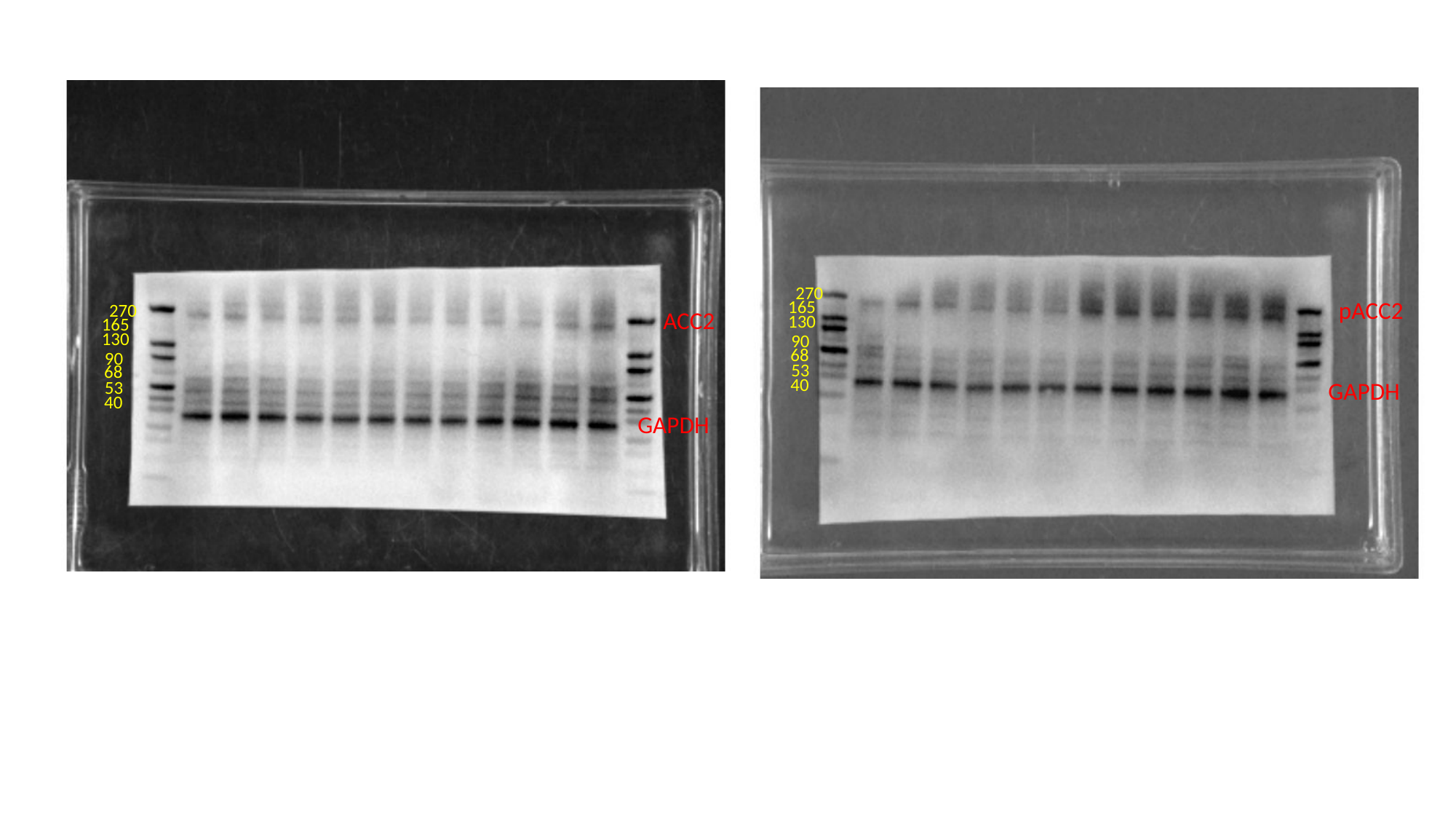

270
165
pACC2
270
ACC2
130
165
130
90
68
90
53
68
40
GAPDH
53
40
GAPDH

## Slide 11
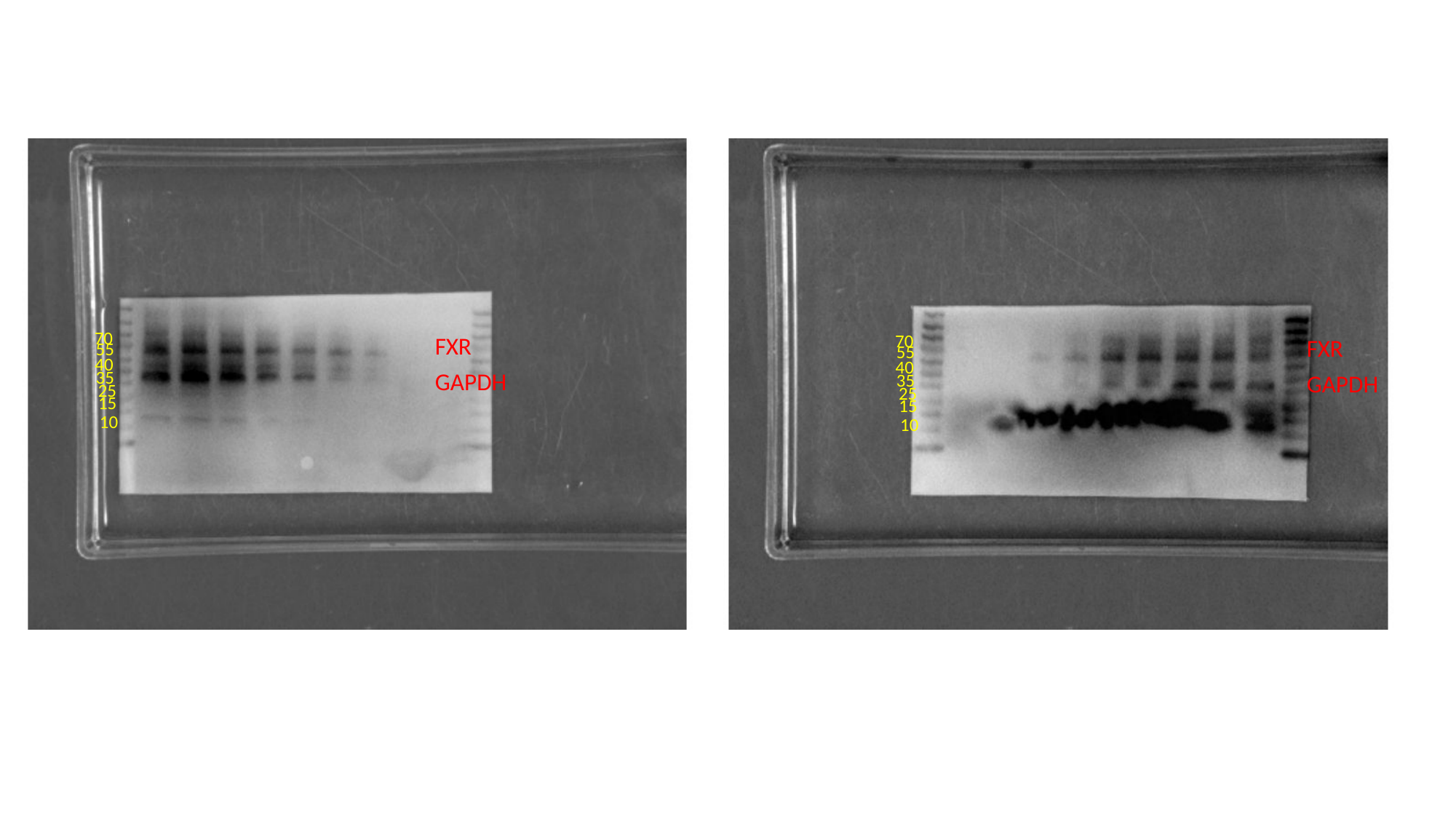

70
70
FXR
FXR
55
55
40
40
35
GAPDH
35
GAPDH
GAPDH
25
25
15
15
10
10

## Slide 12
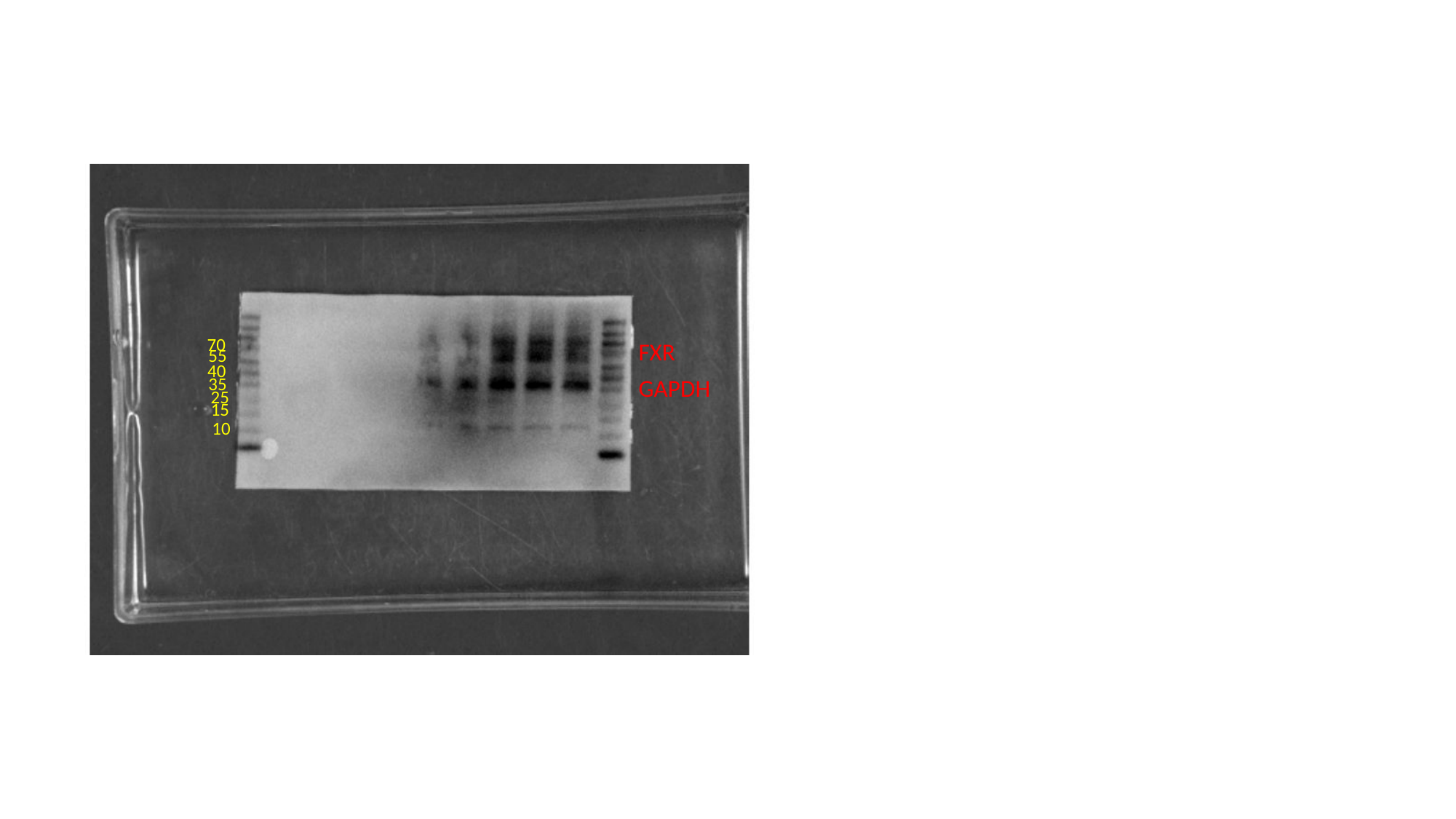

70
FXR
55
40
35
GAPDH
25
15
10

## Slide 13
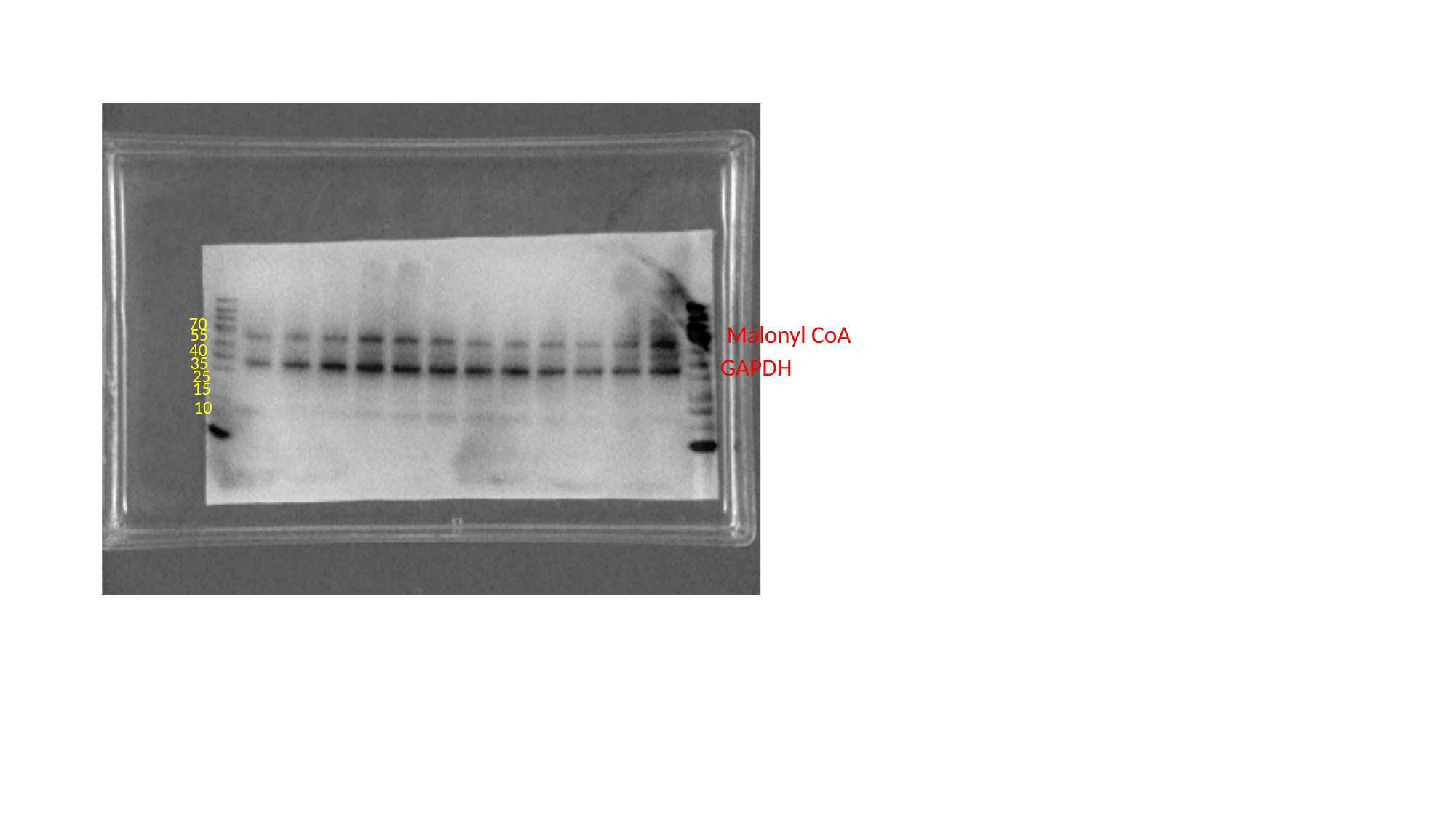

70
Malonyl CoA
55
40
35
GAPDH
25
15
10

## Slide 14
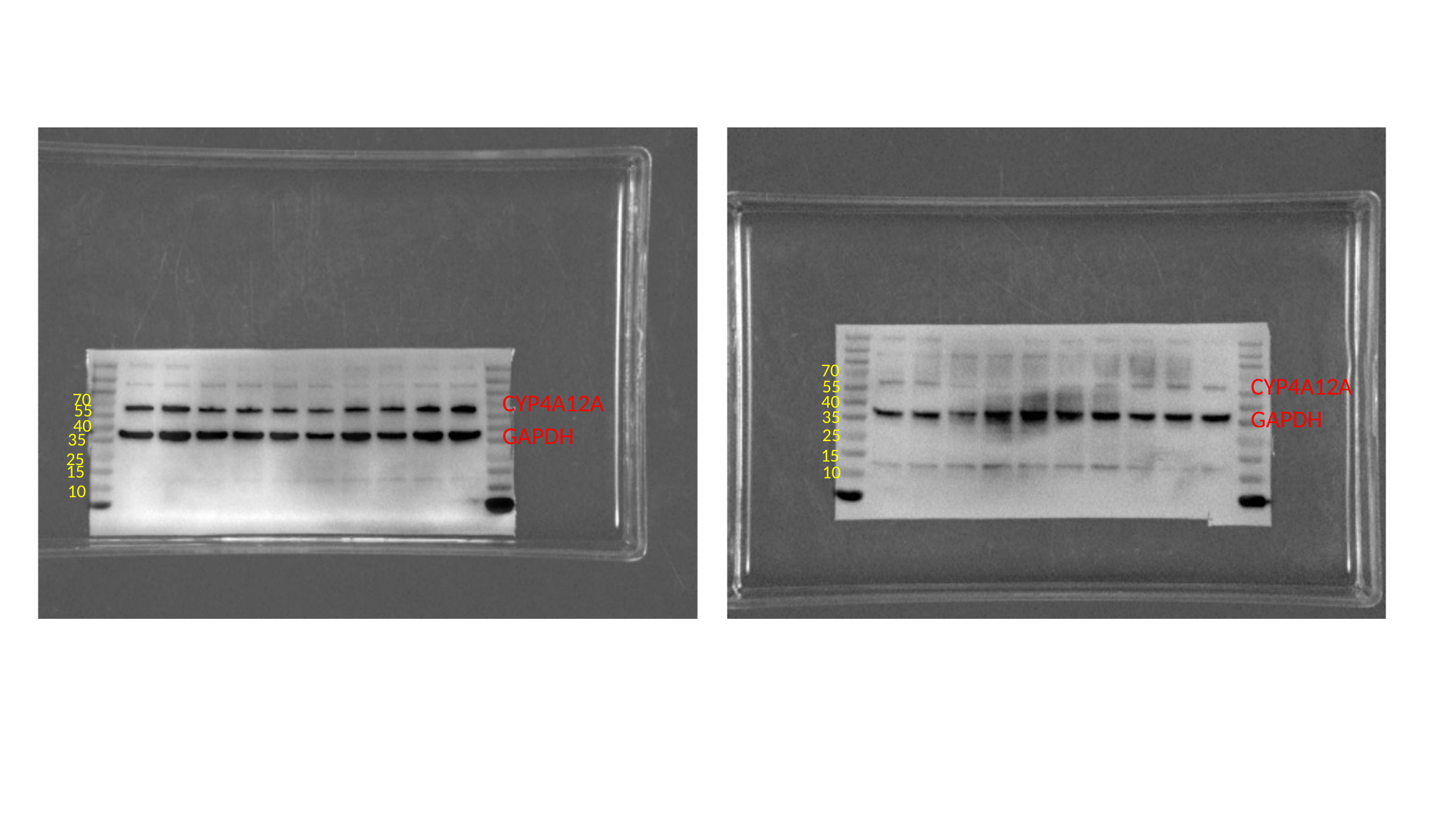

70
CYP4A12A
55
70
CYP4A12A
40
55
GAPDH
35
40
GAPDH
25
35
15
25
15
10
10

## Slide 15
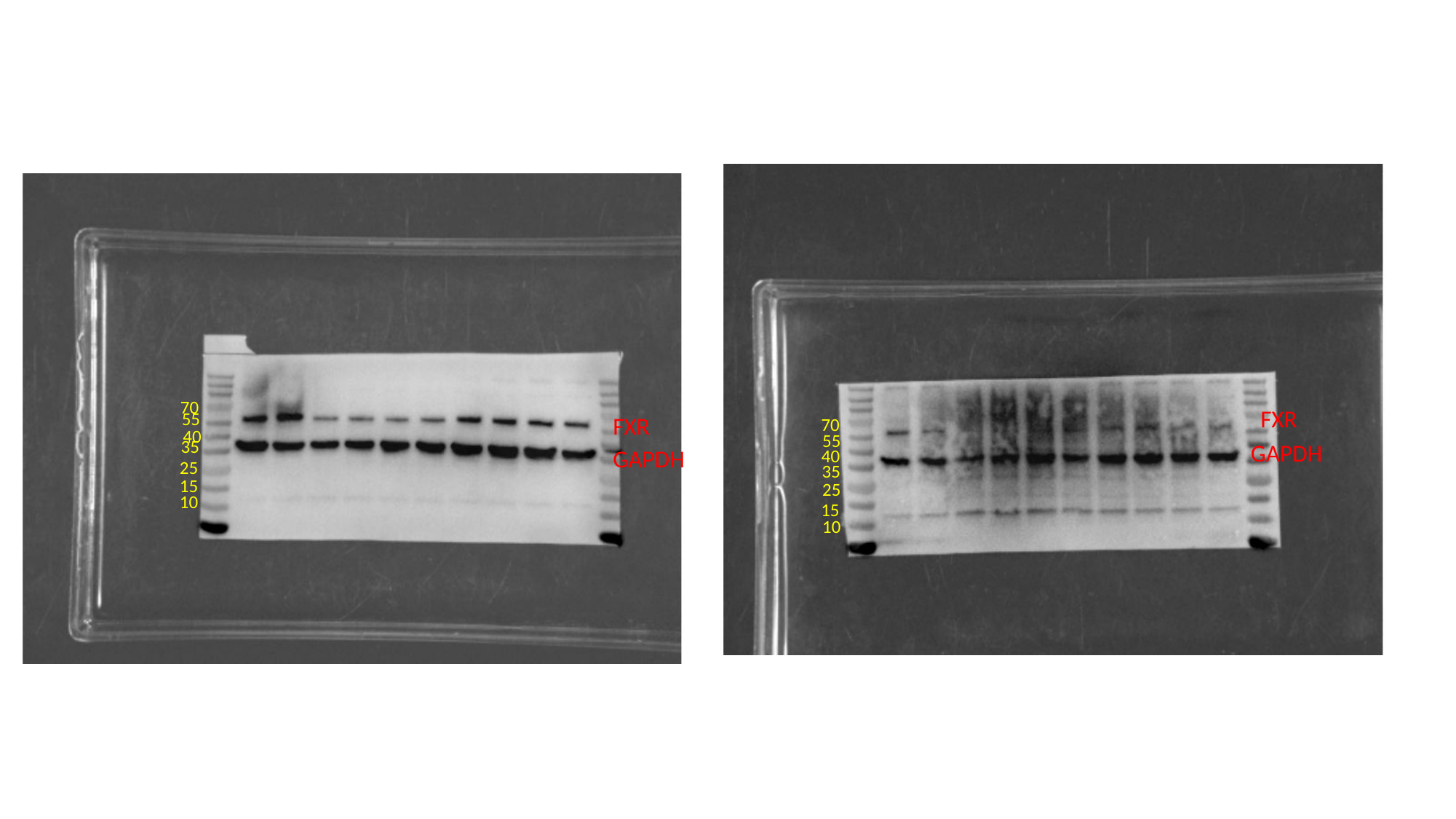

70
FXR
55
FXR
70
40
55
35
GAPDH
GAPDH
40
25
35
15
25
10
15
10

## Slide 16
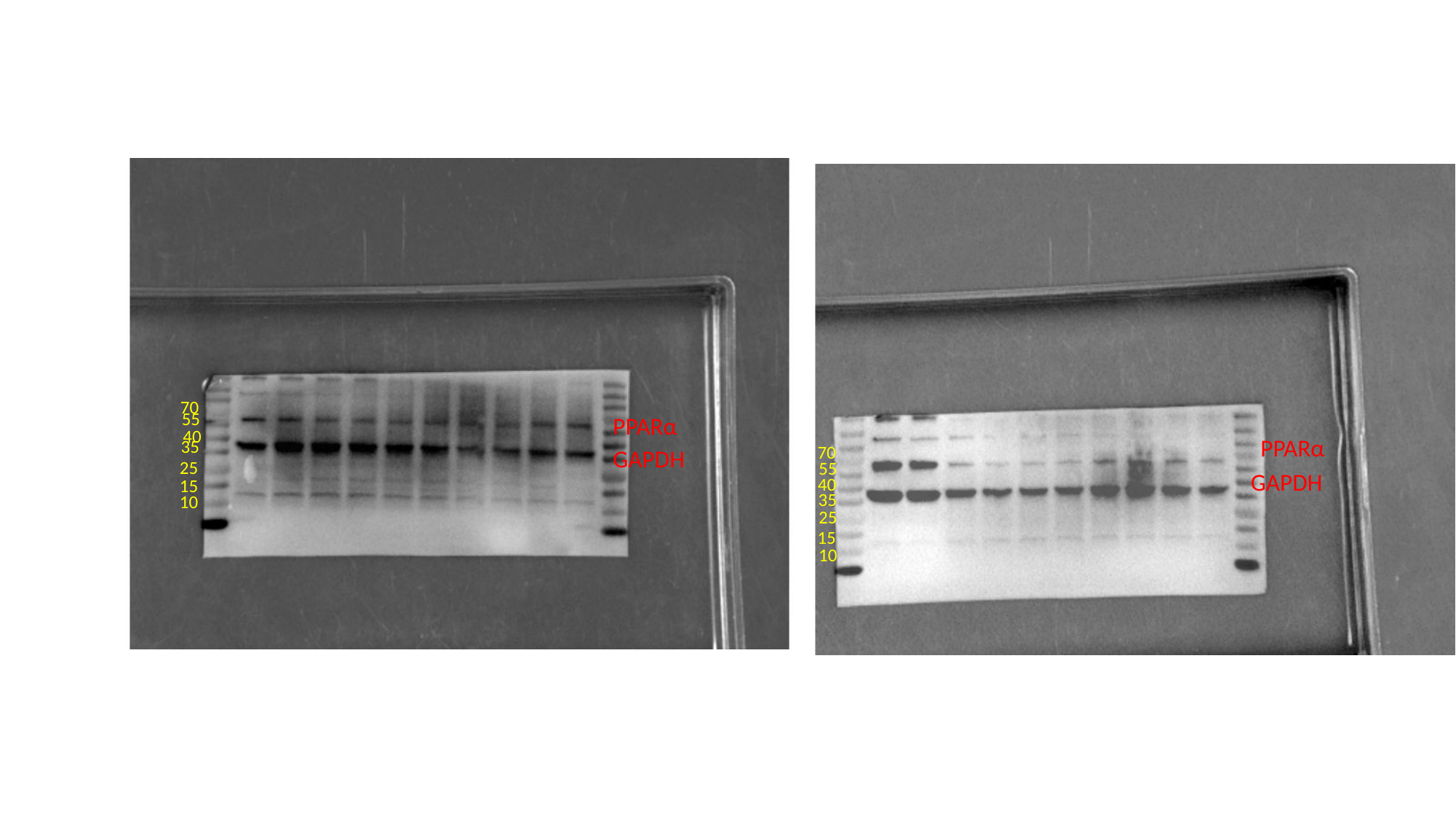

70
55
PPARα
40
PPARα
35
70
GAPDH
25
55
GAPDH
40
15
35
10
25
15
10

## Slide 17
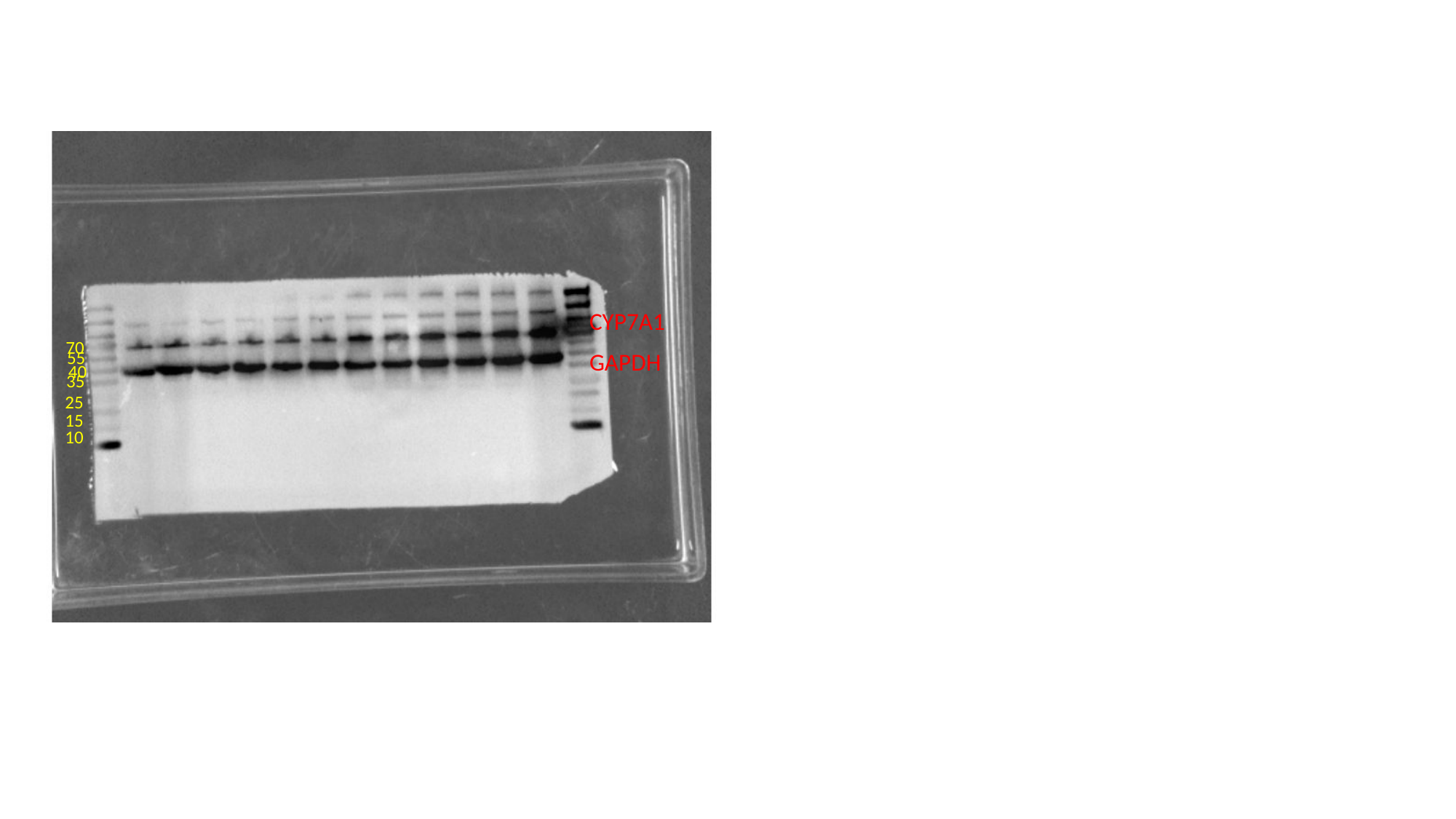

CYP7A1
70
55
GAPDH
40
35
25
15
10

## Slide 18
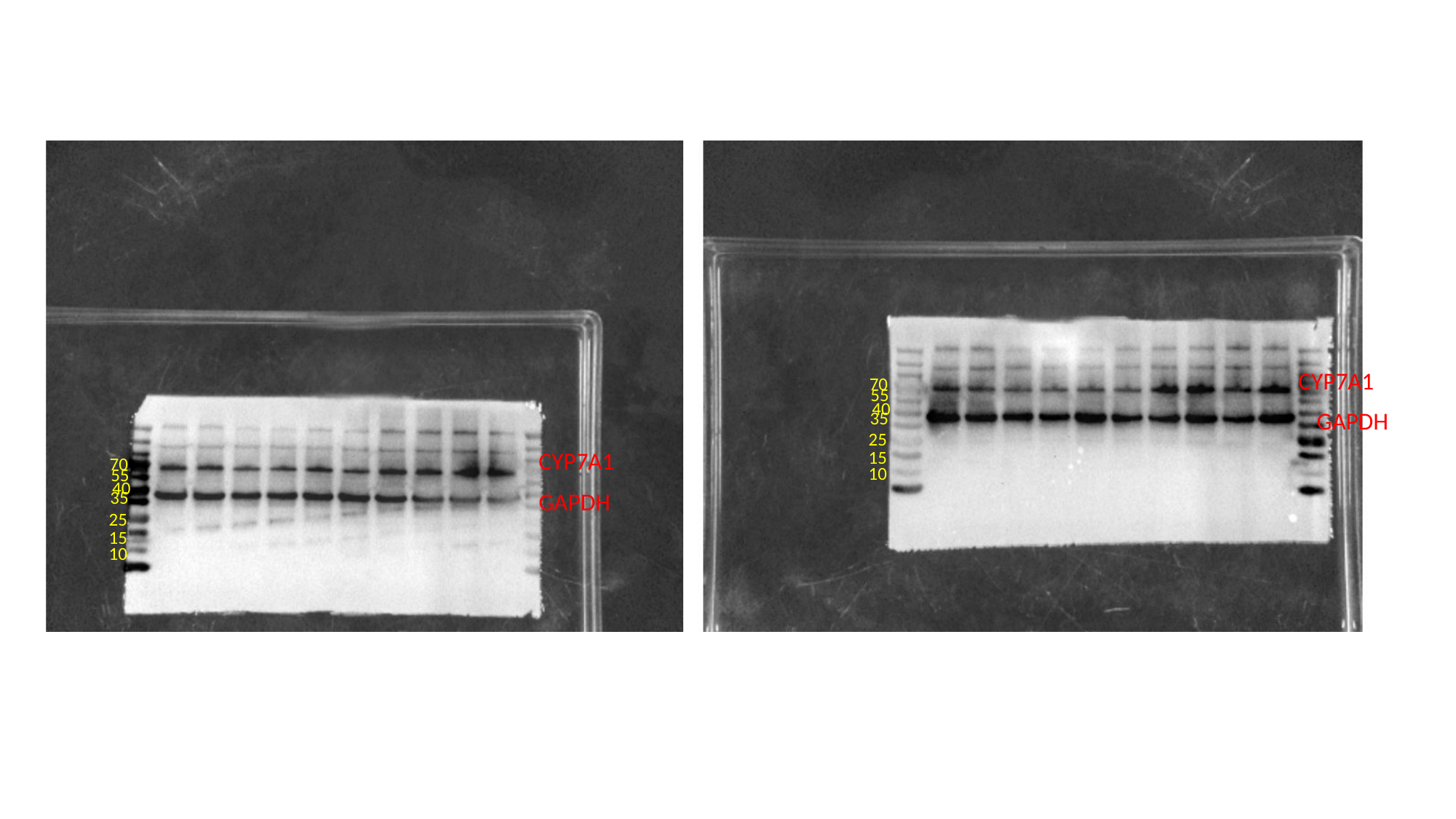

CYP7A1
70
55
40
GAPDH
35
25
CYP7A1
15
70
10
55
40
35
GAPDH
25
15
10

## Slide 19
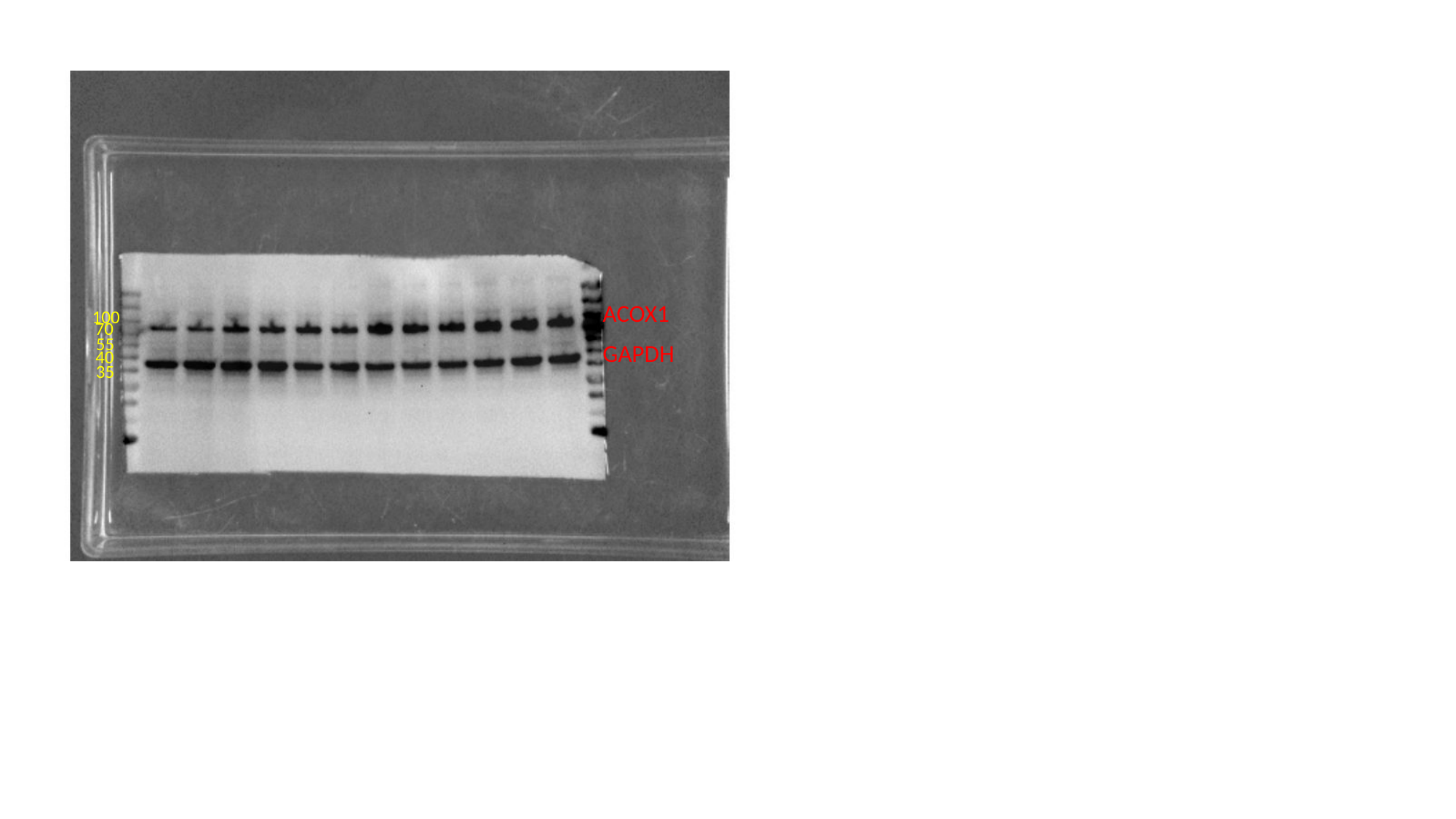

ACOX1
100
70
55
GAPDH
40
35

## Slide 20
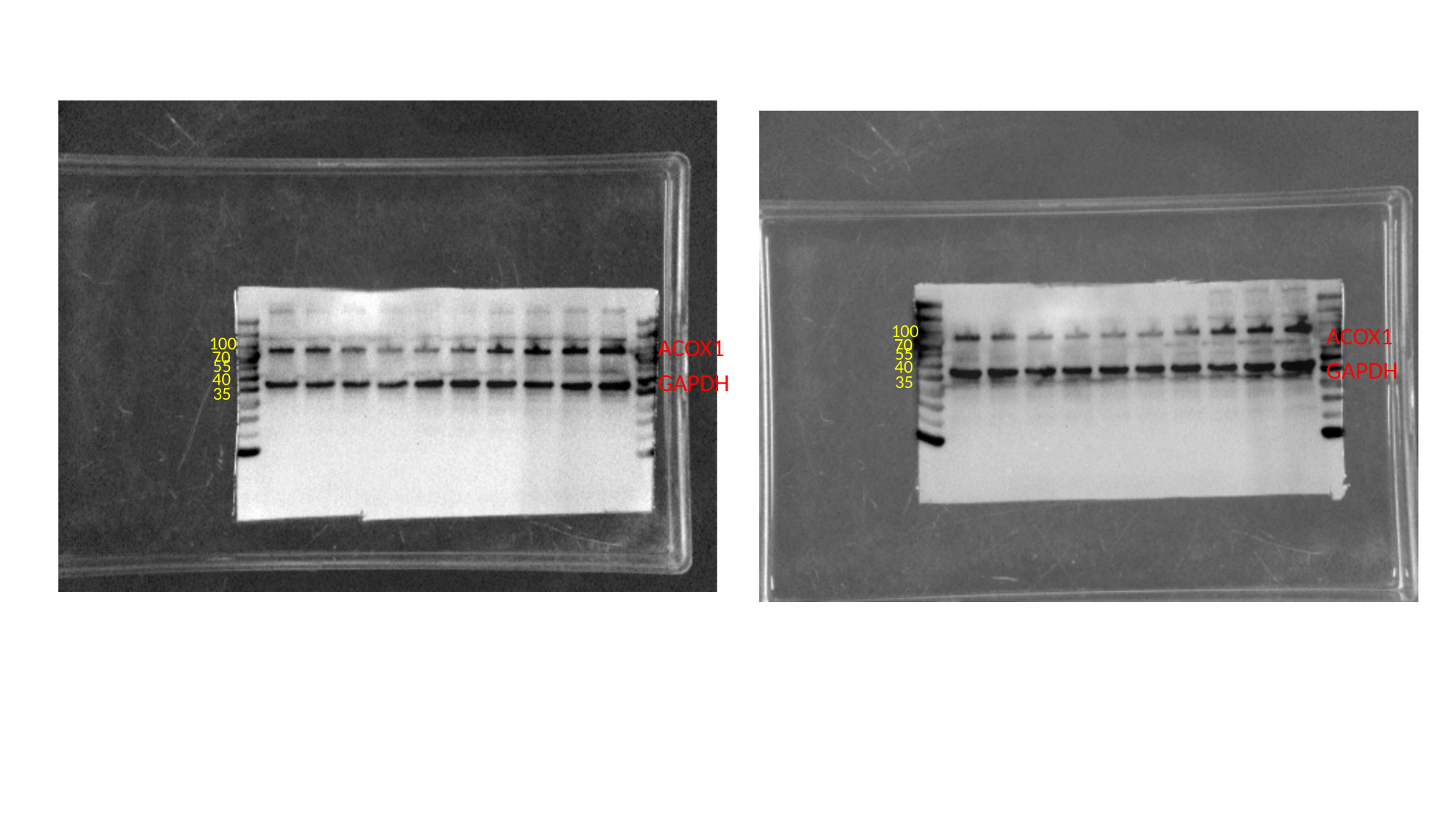

100
ACOX1
100
ACOX1
70
55
70
55
40
GAPDH
40
GAPDH
35
35

## Slide 21
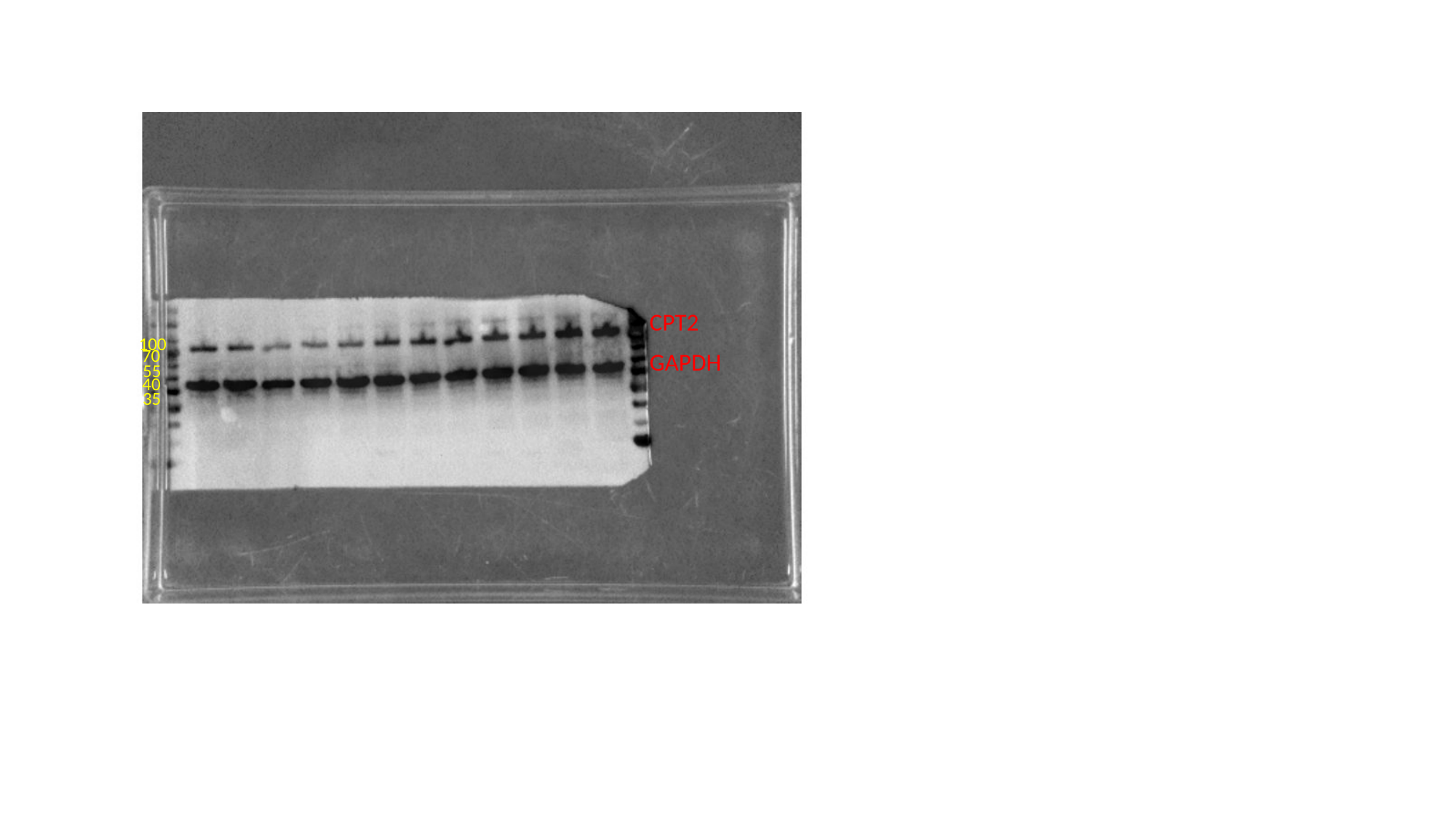

CPT2
100
70
GAPDH
55
40
35

## Slide 22
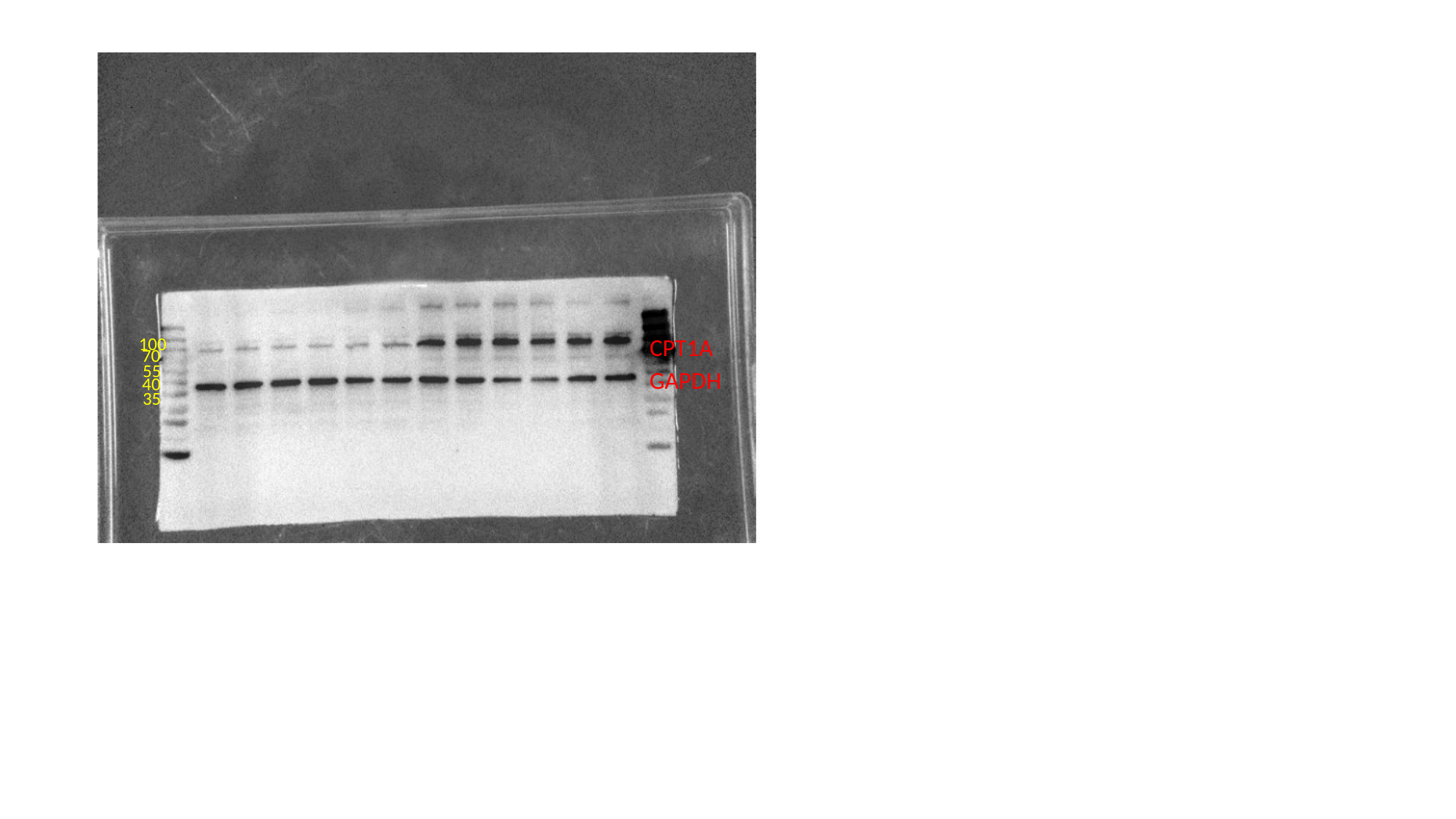

CPT1A
100
70
55
GAPDH
40
35

## Slide 23
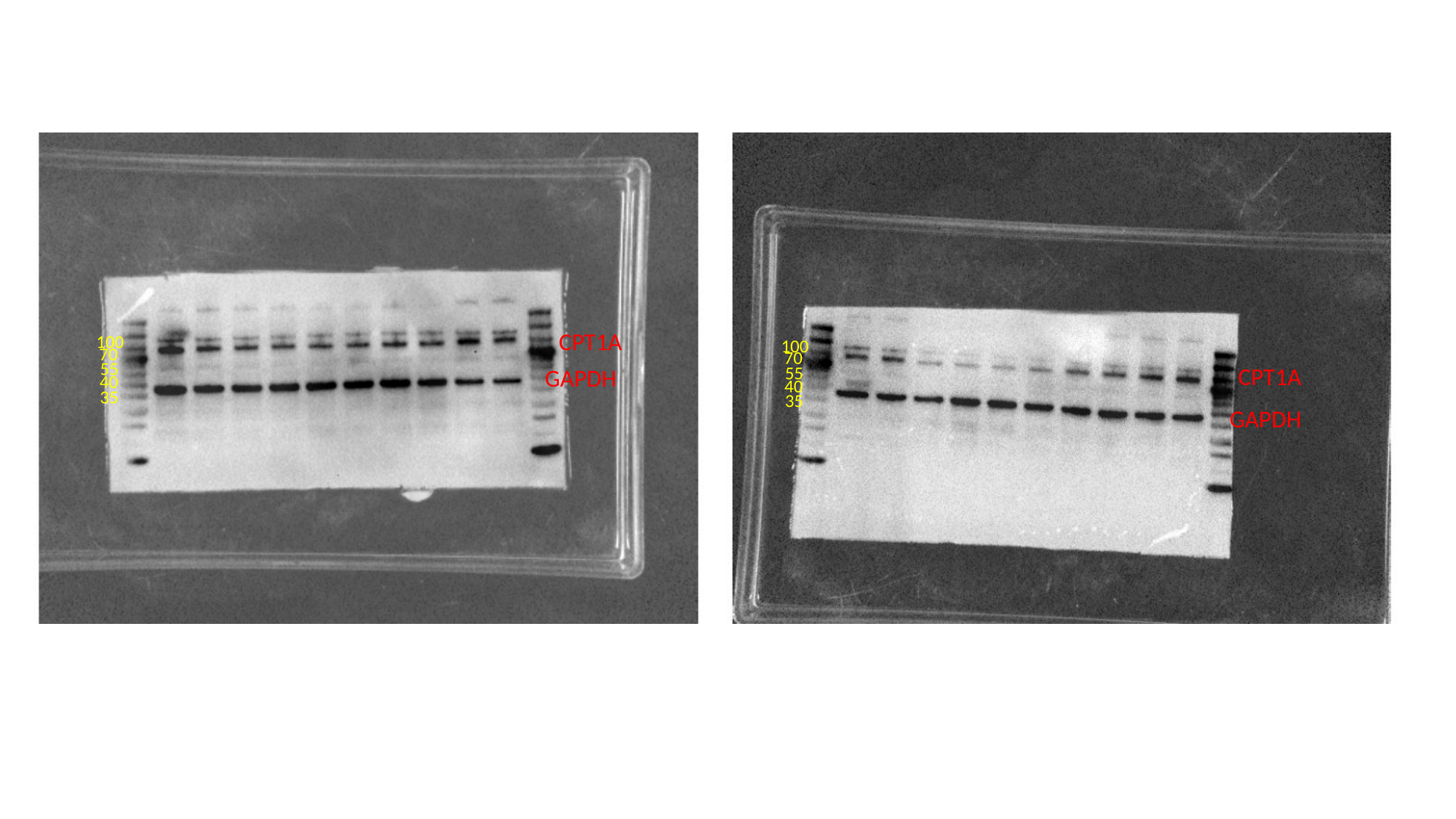

CPT1A
100
100
70
70
55
55
CPT1A
GAPDH
40
40
35
35
GAPDH

## Slide 24
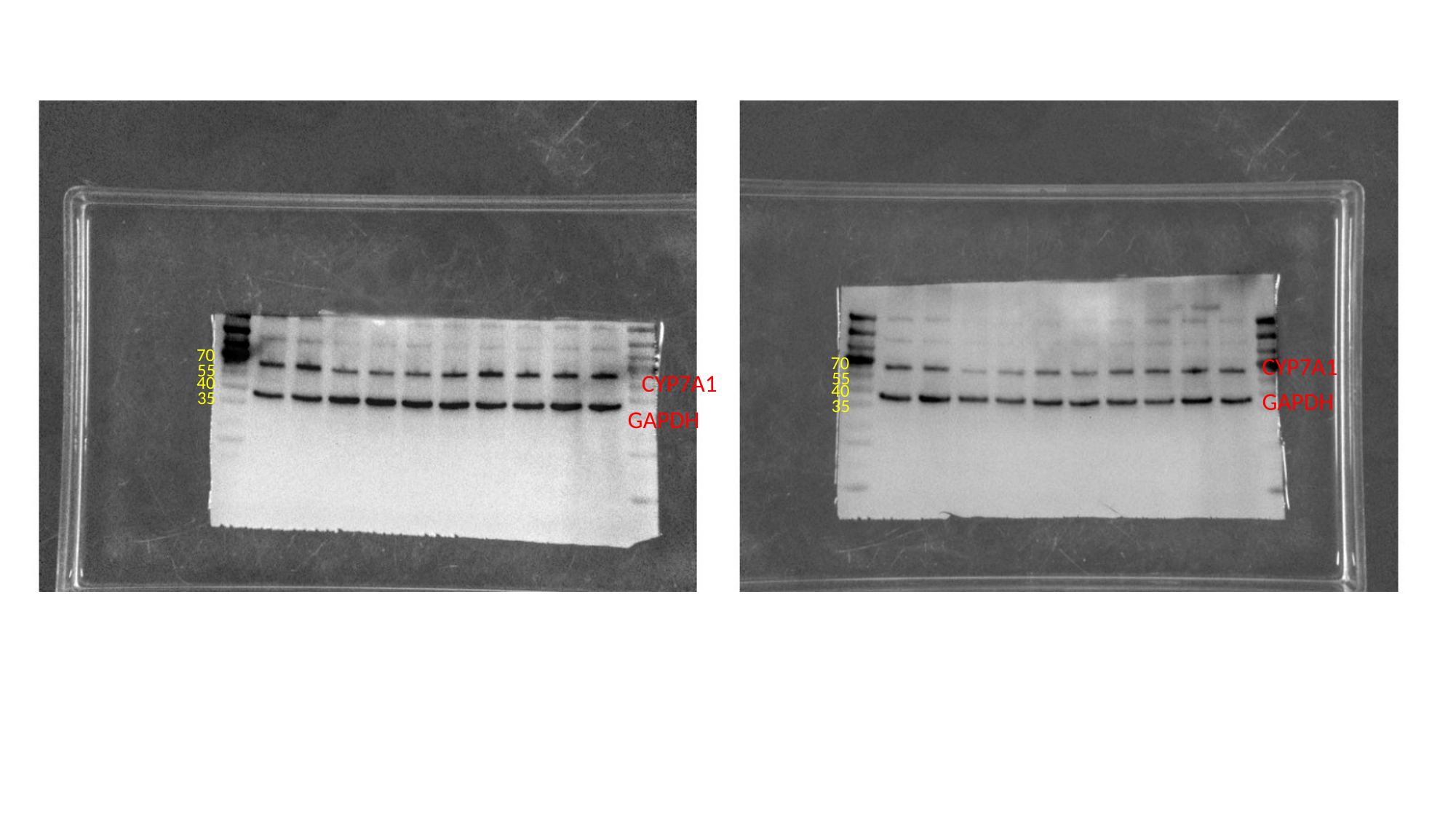

70
70
CYP7A1
55
55
CYP7A1
40
40
35
GAPDH
35
GAPDH

## Slide 25
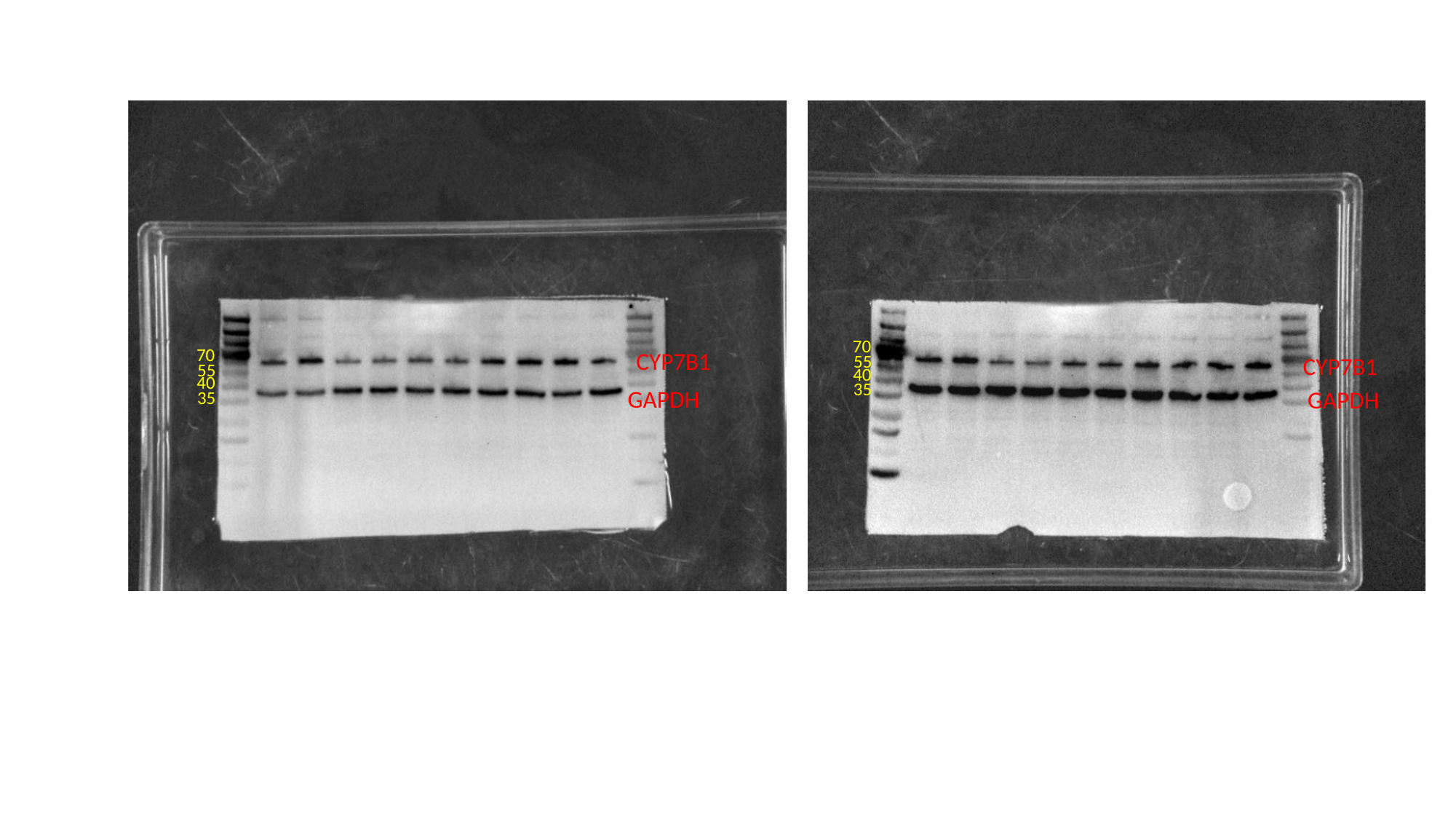

70
70
CYP7B1
55
CYP7B1
55
40
40
35
GAPDH
GAPDH
35

## Slide 26
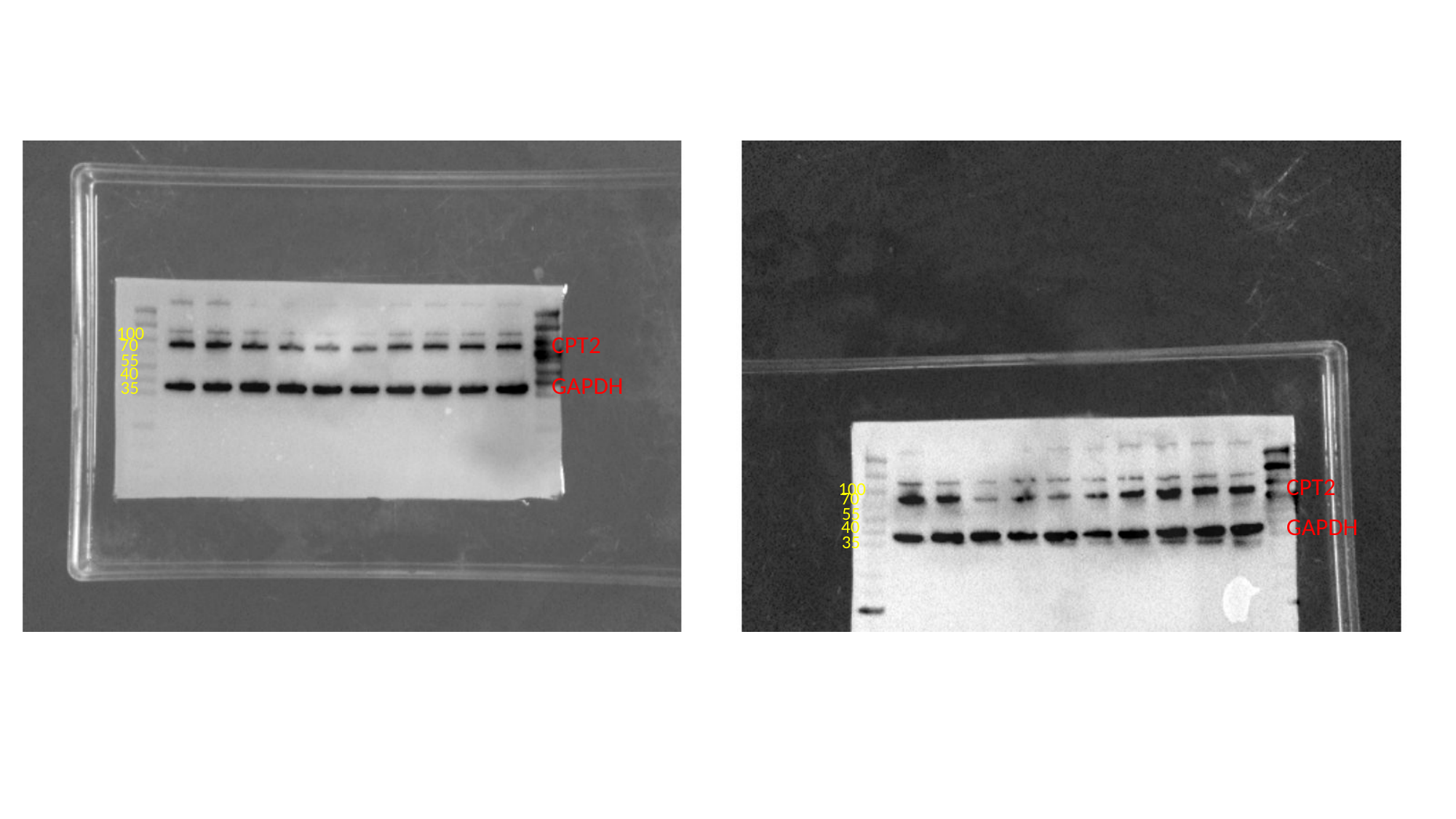

100
CPT2
70
55
40
GAPDH
35
CPT2
100
70
55
GAPDH
40
35
